# Analgesic Differences in Males and Females After Third Molar Surgery: A Subgroup Analysis of the OARS Randomized Clinical Trial

**DOI:** 10.1001/jamanetworkopen.2025.42467

**Published:** 2025-11-06

**Authors:** Janine Fredericks-Younger, Tracy Andrews, Shou-En Lu, Pamela B. Matheson, Patricia Greenberg, Vincent B. Ziccardi, Brent B. Ward, Gary Warburton, Michael Miloro, Hans Malmstrom, Paul J. Desjardins, Cecile A. Feldman

**Affiliations:** 1 Rutgers School of Dental Medicine, Newark, New Jersey; 2 School of Public Health, Rutgers University, Newark, New Jersey; 3 Amherst College, Amherst, Massachusetts; 4 Department of Oral and Maxillofacial Surgery, Rutgers School of Dental Medicine, Newark, New Jersey; 5 Department of Oral and Maxillofacial Surgery, School of Dentistry, University of Michigan, Ann Arbor; 6 Department of Oral and Maxillofacial Surgery, School of Dentistry, University of Maryland, Baltimore; 7 Department of Oral and Maxillofacial Surgery, College of Dentistry, University of Illinois Chicago; 8 Department of General Dentistry, Eastman Institute for Oral Health, University of Rochester, Rochester, New York

## Abstract

This prespecified subgroup analysis of a randomized clinical trial investigates noninferiority for pain relief, treatment effects, and patient satisfaction for nonopioids vs opioids in male and female patients after impacted mandibular third molar extraction.

## Introduction

Sex differences in pain are shaped by a range of biological, psychological, and social factors,^1^ with evidence that women often report greater postsurgical pain^2,3^ and lower pain thresholds and tolerance.^4^ To explore potential variation in pain experience and treatment response, the Opioid Analgesic Reduction Study (OARS)^5,6^ incorporated sex stratification. A prespecified subgroup analysis was conducted to evaluate noninferiority of nonopioids for pain and examined treatment effects on satisfaction and other secondary outcomes between nonopioid and opioid groups within male and female participants separately.

## Methods

Rutgers University Institutional Review Board approved the OARS randomized clinical trial, which includes this subgroup analysis, which is reported following the CONSORT reporting guideline. Participants provided written informed consent. OARS, a multisite, noninferiority trial, compared analgesic effectiveness of a nonopioid combination (ibuprofen 400 mg and acetaminophen 500 mg) with an opioid (hydrocodone 5 mg and acetaminophen 300 mg) for postoperative pain management after impacted mandibular third molar extraction (eMethods in Supplement 1; study protocol in Supplement 2).^5^ Healthy adult participants were randomized by sex and site to receive blinded study analgesic and completed twice-daily electronic diaries postoperatively (eFigure in Supplement 1). A follow-up visit included a clinical exam and survey. Primary outcomes were pain experience and medication satisfaction. Secondary outcomes included sleep quality, pain interference, rescue medication, and adverse events. The noninferiority margin for pain experience was 1.0 points in an 11-point numeric rating scale. Superiority was assessed after establishing noninferiority. Conventional (not noninferiority) testing was used for all other outcomes. Analyses were performed using SAS version 9.4 (SAS Institute). For each outcome, statistical significance was defined by α = .025 (2-sided) for each sex after Bonferroni adjustment to control the overall α of .05, with further adjustments to control for multiple comparisons as appropriate.

## Results

Among 1815 participants (mean [SD] age, 25.7 [6.2] years; 50.1% female; 15.0% Asian, 28.2% Black, 30.9% Hispanic, and 19.7% White), 909 received a nonopioid combination and 906 received an opioid. Sex distribution and baseline and surgical characteristics were comparable across groups (Table 1).

**Table 1.** Baseline and Surgical Characteristics^a^

Characteristic	Patients, No. (%) (N = 1815)			
	Female		Male	
	Nonopioid (n = 457/909 [50.3%])	Opioid (n = 453/906 [49.7%)	Nonopioid (n = 452/909 [50.0%])	Opioid (n = 453/906 [50.0%])
**Demographics**				
Current age, mean (SD), y	25.45 (5.81)	25.70 (6.19)	25.71 (6.13)	25.93 (6.81)
Race and ethnicity^b^				
Hispanic	153 (33.5)	132 (29.1)	102 (22.6)	125 (27.6)
Non-Hispanic Asian	75 (16.4)	89 (19.6)	104 (23.0)	89 (19.6)
Non-Hispanic Black	64 (14.0)	58 (12.8)	77 (17.0)	73 (16.1)
Non-Hispanic White	138 (30.2)	149 (32.9)	141 (31.2)	133 (29.4)
Other	15 (3.3)	12 (2.6)	17 (3.8)	18 (4.0)
Do not want to report	12 (2.6)	13 (2.9)	11 (2.4)	15 (3.3)
Education				
Some high school	19 (4.2)	26 (5.7)	23 (5.1)	31 (6.8)
High school graduate	245 (53.6)	254 (56.1)	241 (53.3)	252 (55.6)
Associate’s degree	61 (13.3)	55 (12.1)	43 (9.5)	34 (7.5)
College graduate	95 (20.8)	83 (18.3)	90 (19.9)	89 (19.6)
Master’s degree	23 (5.0)	20 (4.4)	34 (7.5)	27 (6.0)
Doctoral degree	6 (1.3)	8 (1.8)	15 (3.3)	15 (3.3)
Do not want to report	8 (1.8)	7 (1.5)	6 (1.3)	5 (1.1)
Smoking				
Do not smoke	408 (89.3)	402 (88.7)	390 (86.3)	396 (87.4)
Smoke <1 pack/d	46 (10.1)	48 (10.6)	50 (11.1)	50 (11.0)
Smoke 1 pack/d	3 (0.7)	3 (0.7)	10 (2.2)	5 (1.1)
Smoke >1 pack/d	0 (0)	0 (0)	2 (0.4)	2 (0.4)
Preoperative pain levels				
Pain rating, mean (SD)				
Composite pain experience^c^	1.22 (2.18)	0.88 (1.74)	1.21 (2.14)	1.08 (2.17)
Worst pain^d^	1.77 (2.92)	1.35 (2.56)	1.70 (2.85)	1.54 (2.85)
Average pain^d^	1.32 (2.41)	0.93 (1.85)	1.32 (2.38)	1.12 (2.30)
Least pain^d^	0.79 (1.83)	0.49 (1.26)	0.82 (1.91)	0.75 (1.90)
Pain now^d^	0.99 (2.17)	0.75 (1.83)	0.98 (2.09)	0.89 (2.08)
Pain tolerance^e^	5.56 (2.43)	6.29 (2.04)	5.69 (2.29)	6.04 (2.27)
Preoperative swelling				
None	376 (82.3)	356 (78.6)	399 (88.3)	389 (85.9)
Mild	63 (13.8)	78 (17.2)	40 (8.8)	47 (10.4)
Moderate	14 (3.1)	15 (3.3)	11 (2.4)	11 (2.4)
Severe	4 (0.9)	4 (0.9)	2 (0.4)	6 (1.3)
**Surgical parameters**				
Surgical treatment duration				
Time, mean (SD), min	36.98 (19.21)	40.23 (19.99)	37.35 (19.85)	41.31 (20.27)
Teeth extracted, mean (SD), No.				
Maxillary third molars	1.09 (0.90)	1.09 (0.90)	1.13 (0.91)	1.09 (0.90)
Mandibular third molars	1.67 (0.47)	1.66 (0.48)	1.64 (0.48)	1.65 (0.48)
Total third molars	2.76 (1.17)	2.74 (1.18)	2.77 (1.21)	2.74 (1.21)
Full bony impacted third molars	0.95 (1.26)	0.97 (1.20)	1.00 (1.25)	0.95 (1.19)
Full bony impacted mandibular third molars	0.66 (0.85)	0.71 (0.85)	0.72 (0.84)	0.70 (0.84)
Anesthesia or analgesia used during surgery				
Local only	196 (42.89)	199 (43.93)	239 (52.88)	243 (53.64)
Other (oral, conscious sedation, or nitrous oxide)	75 (16.41)	77 (17.00)	60 (13.27)	58 (12.80)
General anesthesia	186 (40.70)	177 (39.07)	153 (33.85)	152 (33.55)
Other pharmaceutical used				
Antibiotic	84 (18.4)	93 (20.5)	67 (14.8)	66 (14.6)
Anti-inflammatory agent	154 (33.7)	139 (30.7)	129 (28.5)	132 (29.1)
Marcaine	42 (9.2)	38 (8.4)	31 (6.9)	25 (5.5)
Most difficult surgical technique used				
Forceps only	30 (6.6)	32 (7.1)	28 (6.2)	14 (3.1)
Osteotomy	86 (18.8)	87 (19.2)	69 (15.3)	60 (13.2)
Osteotomy with sectioning	341 (74.6)	334 (73.7)	355 (78.5)	379 (83.7)

^a^ The difference in the most difficult surgical technique used in males has *P* = .048 and was therefore included as a covariate in statistical regression analyses. No other differences were found (*P* > .05).

^b^ Race and ethnicity were self-reported by participants. Hispanic was a separate question, and participants who selected Hispanic were categorized as such, regardless of which race they selected. Other race includes Native American, Native Hawaiian, and Other Pacific Islander, or 2 or more races.

^c^ Mean of participant worst, average, least, and pain now ratings.

^d^ On an 11-point numeric rating scale (0 = no pain to 10 = pain as worst as you can imagine).

^e^ On an 11-point numeric rating scale (0 = not tolerant at all to 10 = extremely tolerant).

Nonopioids provided superior pain relief on day 1 for females (mean difference, −0.63; 99.375% CI, −1.04 to −0.21) and males (mean difference, −0.75; 99.375% CI, −1.17 to −0.33). On days 2 and 3 and over the postoperative period, nonopioids were noninferior, with all CIs within the noninferiority margin (d = 1). Patient satisfaction was higher in the nonopioid group; 82.8% of females and 87.8% of males reported being extremely satisfied or satisfied compared with 76.2% and 81.6%, respectively, in the opioid group (females: odds ratio [OR], 1.51; 97.5% CI, 1.04 to 2.20; males: OR, 1.59; 97.5% CI, 1.04 to 2.44) (Table 2).

**Table 2.** Analgesia Differences by Sex in Primary and Secondary Outcomes^a^

Outcome	Female				Male			
	Nonopioid	Opioid	Nonopioid vs opioid comparison		Nonopioid	Opioid	Nonopioid vs opioid comparison	
			Mean difference (99.375% CI^b^)	*P* value^c^			Mean difference (99.375% CI^b^)	*P* value^c^
Pain ratings, mean (95% CI)								
Composite								
First day and night (day of surgery)	3.84 (3.56 to 4.12)	4.46 (4.18 to 4.74)	−0.63 (−1.04 to −0.21)	<.001	3.24 (2.96 to 3.52)	3.99 (3.70 to 4.28)	−0.75 (−1.17 to −0.33)	<.001
Second day and night	3.32 (3.04 to 3.60)	3.60 (3.32 to 3.88)	−0.28 (−0.70 to 0.14)	<.001	2.63 (2.35 to 2.92)	2.89 (2.60 to 3.18)	−0.26 (−0.68 to 0.17)	<.001
Third day and night	3.10 (2.83 to 3.38)	3.18 (2.90 to 3.46)	−0.08 (−0.49 to 0.34)	<.001	2.39 (2.11 to 2.67)	2.47 (2.18 to 2.76)	−0.08 (−0.51 to 0.34)	<.001
Entire postoperative period	2.83 (2.55 to 3.10)	2.98 (2.70 to 3.26)	−0.15 (−0.52 to 0.21)	<.001	2.24 (1.96 to 2.52)	2.37 (2.09 to 2.66)	−0.13 (−0.50 to 0.24)	<.001
Satisfaction, No. (%)								
Very satisfied and satisfied	375 (82.78)	339 (76.18)	OR (97.5% CI) = 1.51 (1.04 to 2.20)	.01	389 (87.81)	364 (81.61)	OR (97.5% CI) = 1.59 (1.04 to 2.44)	.01
Neither satisfied nor dissatisfied, dissatisfied, and very dissatisfied	82 (17.9)	113 (25.0)	NA	NA	64 (14.1)	89 (19.6)	NA	NA
Sleep interference, mean (95% CI)								
Overall sleep quality								
First day (day of surgery)	4.14 (3.79 to 4.49)	4.44 (4.10 to 4.79)	−0.30 (−0.82 to 0.21)	.34	3.88 (3.53 to 4.23)	4.19 (3.83 to 4.56)	−0.32 (−0.84 to 0.21)	.32
Second day	3.96 (3.62 to 4.31)	4.00 (3.65 to 4.35)	−0.03 (−0.55 to 0.48)	.99	3.69 (3.34 to 4.04)	3.68 (3.32 to 4.04)	0.01 (−0.51 to 0.53)	.99
Third day	3.85 (3.50 to 4.19)	3.61 (3.26 to 3.95)	0.24 (−0.28 to 0.76)	.70	3.14 (2.78 to 3.49)	3.43 (3.07 to 3.79)	−0.29 (−0.82 to 0.23)	.41
Entire postoperative period	3.59 (3.27 to 3.92)	3.58 (3.26 to 3.91)	0.01 (−0.39 to 0.41)	.99	3.16 (2.83 to 3.48)	3.26 (2.93 to 3.60)	−0.11 (−0.51 to 0.30)	.99
Pain interference ratings^d^								
Composite								
First day (day of surgery)	2.75 (2.58 to 2.93)	3.10 (2.92 to 3.27)	−0.34 (−0.56 to −0.13)	<.001	2.49 (2.31 to 2.66)	2.98 (2.80 to 3.16)	−0.50 (−0.72 to −0.28)	<.001
Second day	2.24 (2.06 to 2.41)	2.46 (2.28 to 2.63)	−0.22 (−0.44 to −0.00)	.01	1.97 (1.80 to 2.15)	2.22 (2.04 to 2.40)	−0.24 (−0.46 to −0.02)	.005
Third day	2.16 (1.99 to 2.34)	2.26 (2.08 to 2.43)	−0.09 (−0.31 to 0.12)	.85	1.88 (1.71 to 2.06)	2.04 (1.86 to 2.22)	−0.15 (−0.37 to 0.07)	.17
Entire postoperative period	2.07 (1.95 to 2.19)	2.19 (2.07 to 2.31)	−0.12 (−0.28 to 0.04)	.14	1.87 (1.75 to 2.00)	2.02 (1.89 to 2.14)	−0.14 (−0.31 to 0.02)	.05
Safety measures								
Postoperative rescue opioid prescribed, patients, No. (%)	16 (3.52)	29 (6.50)	OR (97.5% CI) = 0.50 (0.24 to 1.04)	.04	10 (2.25)	25 (5.63)	OR (97.5% CI) = 0.39 (0.16 to 0.94)	.02
No. of rescue pills prescribed, mean (95% CI)	9.75 (6.78 to12.72)	8.73 (5.84 to 11.63)	1.02 (−1.18 to 3.21)	.29	10.09 (7.09 to 13.10)	9.50 (6.70 to 12.30)	0.59 (−1.95 to 3.14)	.59
No. of nonopioid or opioid tablets returned, mean (95% CI)	7.24 (5.52 to 8.96)	8.91 (7.19 to 10.62)	−1.66 (−2.59 to −0.74)	<.001	6.80 (5.07 to 8.54)	7.93 (6.20 to 9.67)	−1.13 (−2.06 to −0.20)	.007
Patients with AEs requiring clinic visit, No. (%)	20 (4.38)	19 (4.19)	OR (97.5% CI) = 1.10 (0.52 to 2.31)	.77	18 (3.98)	19 (4.19)	OR (97.5% CI) = 0.98 (0.46 to 2.09)	.95
Severity of AEs, mean (95% CI)								
Requiring clinic visit	1.27 (1.23 to 1.32)	1.34 (1.30 to 1.39)	−0.07 (−0.12 to −0.02)	.002	1.22 (1.17 to 1.27)	1.27 (1.23 to 1.32)	−0.06 (−0.11 to −0.00)	.02
Reported in eDiary	1.78 (1.22 to 2.34)	1.87 (1.32 to 2.41)	−0.09 (−0.61 to 0.44)	.71	1.35 (0.83 to 1.88)	1.87 (1.28 to 2.45)	−0.51 (−1.05 to 0.03)	.03

Abbreviations: AE, adverse event; eDiary, electronic diary; NA, not applicable; OR, odds ratio.

^a^ Analyses were intent to treat, using generalized linear mixed models and adjusted for the baseline covariate, the most difficult surgical technique. For each outcome, statistical significance was defined by α = .025 (2-sided) for each sex after Bonferroni adjustment to control the overall α of .05, with further adjustments to control for multiple comparisons as appropriate.

^b^ The 2-sided 99.375% CI of μ_NonOpioid,_*_t_* − μ_Opioid,_*_t_* for pain ratings was constructed to test the noninferiority of the nonopioid at 4 times: *t* = first day and night, second day and night, third day and night, and the entire postoperative period after the Bonferroni adjustment to control the overall 2-sided α = .025 for each sex. Noninferiority was concluded if the entire CI lay below the noninferiority margin *d* = 1.0. Statistical superiority was concluded if the CI lay completely below 0.

^c^ *P* values reported for pain ratings were from testing: H_0_: (μ_Nonopioid,_*_t_* − μ_Opioid,_*_t_*) ≥ 1 vs H_1_: (μ_Nonopioid,_*_t_* − μ_Opioid,_*_t_*) < 1, for *t* = 4 times with Bonferroni adjustment to control the overall α = .0125 (1-sided) for each sex. *P* values for satisfaction and safety measures were not adjusted for time. *P* values reported for the mean differences of the other outcomes were from testing: H_0_: (μ_Nonopioid,_*_t_* − μ_Opioid,_*_t_*) = 0 vs H_1_: (μ_Nonopioid,_*_t_* − μ_Opioid,_*_t_*) not = 0, for *t* = 4 times with Bonferroni adjustment to control overall α = .025 (2-sided) for each sex.

There were no significant differences in sleep quality across groups. However, participants not receiving opioids experienced less pain interference on days 1 and 2. The need for rescue medication was lower in the nonopioid group for males but not significantly different for females. Adverse events requiring emergency visits were less severe (0-3 scale where 0 = none and 3 = severe) in the nonopioid group for both sexes, and electronic diary–reported adverse events were milder in males not receiving opioids (Table 2).

## Discussion

In the overall OARS cohort in this prespecified subgroup analysis of a randomized clinical trial, nonopioid therapy provided superior pain control compared with opioid therapy during the first and second day-night periods after surgery. Thereafter, noninferiority was observed during the third day-night period and across the full 7-day postoperative interval. In subgroup analysis by sex, superiority was retained for the first day-night, while noninferiority was consistently observed for the second and third day-night periods and the entire 7-day follow-up.

These results support the overall OARS conclusion that nonopioid therapy was at least as effective as and often superior to opioid therapy for postoperative pain management. The consistency of treatment effects across male and female participants supports the robustness of the trial’s findings, underscoring the clinical utility of nonopioids as first-line therapy.

A key limitation of this analysis is that it focused on treatment effects within sex subgroups without examining sex-based differences between treatment groups. Nonetheless, the alignment of subgroup and overall results provides strong confirmatory evidence for the main trial conclusions.

## Notes

Supplement 1.
eMethods.

eReferences.
**eFigure.** Participant Flow in the OARS Trial

Supplement 2.
Trial Protocol and SAP

Supplement 3.
Data Sharing Statement
