# Supplementary material for: Analgesic Differences in Males and Females After Third Molar Surgery: A Subgroup Analysis of the OARS Randomized Clinical Trial
Source: JAMA Netw Open. 2025 Nov 6;8(11):e2542467. doi: 10.1001/jamanetworkopen.2025.42467 (PMC12593115; doi:10.1001/jamanetworkopen.2025.42467)
Supplement: Supplement 1. — eMethods. eReferences. eFigure. Participant Flow in the OARS Trial [file jamanetwopen-e2542467-s001.pdf]

Supplemental Online Content

Fredericks-Younger J, Andrews T, Lu S, et al. Analgesic differences in males and females after third molar surgery. *JAMA Netw Open*. 2025;8(11):e2542467. doi:10.1001/jamanetworkopen.2025.42467

eMethods.
eReferences.
eFigure. Participant Flow in the OARS Trial

This supplemental material has been provided by the authors to give readers additional information about their work.

© 2025 Fredericks-Younger J et al. *JAMA Network Open*.

eMethods

Trial Oversight

Rutgers University Institutional Review Board (IRB) served as the sIRB of record (Pro2020002299). All participants provided written informed consent. The National Institute of Health appointed a Data and Safety Monitoring Board and contracted a clinical monitoring agency for trial oversight. The OARS Trial was registered on ClinicalTrials.gov Identifier: NCT04452344 on 4/07/2020.

Study Design

The Opioid Analgesic Study (OARS) was a randomized, multi-site, non-inferiority clinical trial that compared analgesic effectiveness of a combination of non-opioids (ibuprofen 400mg and acetaminophen 500mg) versus an opioid (hydrocodone 5mg/ acetaminophen 300mg) in the management of acute post-operative pain.[27] Using the Dental Impaction Pain Model (DIPM), participants underwent planned surgery to extract one or more impacted mandibular third molars, took assigned study analgesic as needed for pain, and electronically recorded pain related outcomes over the duration of the post-operative period. This trial was conducted between 1/7/21 and 6/30/23.

Participants

Study participants consisted of interested adult patients (aged 18 years or older) who presented to one of five academic dental centers (University of Illinois at Chicago, University of Maryland, University of Michigan, University of Rochester, or Rutgers University) for planned extraction of at least one impacted mandibular third molar. All participants were required to understand and provide informed consent, be willing to comply with study procedures, and be available for the duration of the study. They must have been able to safely take ibuprofen, acetaminophen, and hydrocodone. Female participants were required to have a negative pregnancy test and agree to use alternative contraception during the study. Exclusion criteria focused on medical contraindications associated with the study analgesics (ibuprofen, acetaminophen, hydrocodone) and positive social history of drugs and alcohol for the patients and their first-degree relatives. Subjects were excluded if they self-reported a history of gastrointestinal bleeding, peptic ulcer, renal disease (excluding kidney stones), hepatic disease, bleeding disorders, or respiratory depression, including any prior postoperative respiratory support due to opioids or anesthetics. Additional exclusions included active or untreated asthma, known allergies to ibuprofen, acetaminophen, hydrocodone, or anesthesia, current use of CYP3A4 inhibitors, azole antifungals, protease inhibitors, or CNS

© 2025 Fredericks-Younger J et al. *JAMA Network Open*.

depressants, and a history of drug or alcohol abuse, including daily consumption of three or more alcoholic drinks or a first-degree relative with substance abuse history. Participants were also excluded if they had filled an opioid prescription within the past six months, were pregnant or lactating, or had previously participated in the study.

Randomization and treatment

Following a randomization sequence defined by the lead statistician, participants were randomized, stratified by sex and within study site, in a 1:1 ratio to one of two analgesic treatment groups: non-opioid (ibuprofen 400mg + acetaminophen 500mg) or opioid (hydrocodone 5mg/acetaminophen 300mg + placebo). The medication was over-encapsulated to maintain masking of the treatment assignment for the patient and the study site team. Participants received 20 doses of blinded study analgesic in two electronic medication bottles and were instructed to take one dose of study analgesic every four to six hours as needed for pain.

Study Procedures and Outcomes

Interested patients who presented to the dental centers for planned extraction of at least one impacted mandibular third molar were consented for participation. Eligibility was determined (Visit 0) based on inclusion/exclusion criteria. Participants self-reported their race, ethnicity, medical history, social history, and biologic sex (defined as male or female). Individuals participating in the study self-reported their race as African American, Caucasian, Native American, Native Hawaiian or Other Pacific Islander, Asian, Two or more races, or Do not want to report. Participants self-reported their ethnicity as Hispanic, Non-Hispanic, or Do not want to report. Reporting race and ethnicity in this study was mandated by the US National Institutes of Health (NIH), consistent with the Inclusion of Women and Minorities policy and categories were based on NIH requirements at the time of application and award.

On the day of surgery (Visit 1), eligibility was reconfirmed through a prescription drug monitoring program (PDMP) check and a negative pregnancy test (for females). Baseline characteristics related to pain were collected and participants were randomized to one of the two analgesic treatment groups, non-opioid or opioid, and received their assigned, blinded study medication as part of their post-randomization subject kit. Surgery was performed and a surgical case report completed. Escorted participants took their first analgesic dose in the office immediately after surgery, while unescorted participants took their first dose upon arrival home. Over the postoperative period (9 days

© 2025 Fredericks-Younger J et al. *JAMA Network Open*.

post-surgery +/- 5 days), participants were instructed to manage pain with study analgesic every 4-6 hours as needed for pain. Participants could substitute study analgesic with 400mg ibuprofen. Participants completed twice-daily electronic diaries, which documented pain, pain interference, sleep quality, adverse events/side effects, and the need for rescue analgesic. At the post-operative visit (Visit 2), participants received a clinical exam and completed a study follow-up questionnaire aimed at understanding pain, pain interference, sleep quality, adverse events, and satisfaction with the medication.

The OARS trial focused on two primary outcomes: pain experience and overall satisfaction with pain medication. Pain experience was assessed using the composite pain experience rating, which was an average of the four pain items on the Brief Pain Inventory (average pain, worst pain, least pain, and pain right now) for a given time period: 1st day/night, 2nd day/night, 3rd day/night & entire post-operative period. Each item assessed pain using a NRS scale which ranged from 0="no pain" to 10="worst pain imaginable". Overall satisfaction with pain medication was assessed at the post-operative visit using a 5-point Likert scale with 1="very satisfied" to 5="very dissatisfied". Overall satisfaction data was reported dichotomously in terms of very satisfied/satisfied versus neither satisfied nor dissatisfied/dissatisfied/very dissatisfied.

Several secondary patient-centered outcomes were also evaluated, including overall sleep quality [NRS scale (0="excellent" to 10="very poor" sleep], composite pain interference (average of 6 questions derived from a modified Patient Reported Outcomes Measurement Information System- Pain Interference Short Form 6b using a 5-point Likert scale (1="not at all" to 5="very much"), need for rescue medication, and adverse events (severity and frequency).

Statistical Analysis

All statistical analyses were performed on an intent-to-treat (ITT) basis. To test whether non-opioid medication was non-inferior to opioids for pain in each sex subgroup (women and men), we used mixed model analysis with pain modeled as a function of the main effects as well as the two-way and three-way interactions of treatment (NON-OPIOID vs. OPIOID), day, and sex as the fixed effect independent variables; participants and clinical sites were nested random effects to account for the intra-participant and intra-cluster correlations. To test whether non-opioid

© 2025 Fredericks-Younger J et al. *JAMA Network Open*.

medication was non-inferior to opioids for pain, we determined the non-inferiority margin (d) as 1.0 on the 11-point NRS (from 0-10).[1-3] Mean differences in pain between the treatment groups (analgesia effect) for females and males were estimated using the linear contrasts. In this prespecifed subgroup analysis, the non-inferiority of non-opioids was tested for each sex in 4 time comparisons using the 2-sided 99.375% confidence interval (CI) of $\mu_{NonOpioid,t}$ - $\mu_{Opioid,t}$, for t= 1, 2, 3, and 4 for the 1st day/night, 2nd day/night, 3rd day/night & the entire post-operative period, respectively, after the Bonferroni adjustment to control the overall 2-sided alpha at 2.5% for each sex subgroup. If the entire CI was completely below d=1.0, we concluded the non-inferiority of the non-opioid. If non-inferiority was established, we assessed and concluded (statistical) superiority by determining whether the CI completely laid below 0. In addition, we also reported the p-values from testing the non-inferiority of non-opioid using the 1-sided test: H_0: $(\mu_{NONOPIOID,t} - \mu_{OPIOID,t}) \geq d$ vs H_1: $(\mu_{NONOPIOID,t} - \mu_{OPIOID,t}) < d$, for t= first day/night, 2nd day/night, 3rd day/night,, and the entire postoperative period, with the Bonferroni adjustment to control the overall alpha at 1.25% (1-sided) for each sex.

Satisfaction with pain medication at the post-operative visit was treated as a categorical variable and compared between non-opioid vs opioid groups using the random effect logistic regression model with treatment, sex and treatment by sex interactions as the fixed effect independent variables and clinical site as a random effect. Comparisons between non-opioid vs opioid in each sex subgroup were tested using linear contrasts.

Secondary outcomes including pain interference and sleep quality were compared using the mixed model analysis similar to that described for pain. The need for rescue medication and occurrence of adverse events were compared using the random effect logistic regression analysis similar to that described for satisfaction with pain medication. Except for pain, all the other outcomes were tested using the conventional tests, not as a non-inferiority test.

All regression analyses were adjusted for most difficult surgical technique utilized due to imbalanced distribution between non-opioid and opioid in males. For each outcome, statistical significance was defined by alpha=0.05, after the Bonferroni adjustment to control the overall two-sided alpha of 0.025 for each sex subgroup. Bonferroni multiple testing adjustment was further applied to all analyses that included the four time point comparisons. All statistical analyses were performed using SAS v.9.4.

© 2025 Fredericks-Younger J et al. *JAMA Network Open*.

Power and Sample Size considerations

To test non-inferiority of non-opioids for pain, we determined the sample size on the basis of data from Chang et al[4]. (SD, 3.6), the non-inferiority margin d of 1.0 established before the start of the trial, and applied the Bonferroni adjustment for 4 times to control the overall alpha at 1-sided 2.5% (2-sided 5% equivalent) with 90% power in the full sample analysis and greater than 80% power in the subgroup analysis according to sex. To account for 15% through 20% loss of follow-up and missing data, and other factors not included in the sample size estimation, we planned to recruit 1,800 participants, with 450 participants in each group (2 analgesic groups x 2 sex subgroups (female and male participants)). Specifically, for the subgroup analysis by sex, with n=370/analgesic group (after attrition/missing data, etc.), non-inferiority margin d=1.0 and alpha=0.3125% (one-sided), we have 85% power to test non-inferiority of non-opioid analgesics in female and male participants separately. For patient satisfaction with medication, we assumed the proportion of positive rating (very satisfied and satisfied) for the non-opioid in our study to be 82%, similar to Daniels et al.,[5] our study has 85% power to test a minimal difference of 11% (82% vs. 71%) for n=370/analgesic group (subgroup analysis, 2-sided alpha=2.5%), in comparing proportion of positive rating of patient satisfaction.

Halting Rules

The OARS study would be suspended for safety review if there is a fatality due to the study analgesic, or hospitalization of two or participants due to the same serious adverse event. There were no predefined criteria for participant withdrawal. No participants were withdrawn by the investigators except in cases involving a serious adverse event. These individuals were excluded from the data analysis.

Methods eReferences

1. Feldman CA, Fredericks-Younger J, Desjardins PJ, et al. Nonopioid vs opioid analgesics after impacted third-molar extractions: The Opioid Analgesic Reduction Study randomized clinical trial. J Am Dent Assoc. 2025;156(2):110-123.e9. doi:10.1016/j.adaj.2024.10.014

© 2025 Fredericks-Younger J et al. *JAMA Network Open*.

2. Todd KH, Funk KG, Funk JP, Bonacci R. Clinical significance of reported changes in pain severity. Ann Emerg Med. 1996;27(4):485-489. doi:10.1016/s0196-0644(96)70238-x

3. Gallagher EJ, Liebman M, Bijur PE. Prospective validation of clinically important changes in pain severity measured on a visual analog scale. Ann Emerg Med. 2001;38(6):633-638. doi:10.1067/mem.2001.118863

4. Chang AK, Bijur PE, Esses D, Barnaby DP, Baer J. Effect of a Single Dose of Oral Opioid and Nonopioid Analgesics on Acute Extremity Pain in the Emergency Department: A Randomized Clinical Trial. JAMA. 2017;318(17):1661-1667. doi:10.1001/jama.2017.16190

5. Daniels SE, Goulder MA, Aspley S, Reader S. A randomised, five-parallel-group, placebo-controlled trial comparing the efficacy and tolerability of analgesic combinations including a novel single-tablet combination of ibuprofen/paracetamol for postoperative dental pain. Pain. 2011;152(3):632-642. doi:10.1016/j.pain.2010.12.012

© 2025 Fredericks-Younger J et al. *JAMA Network Open*.

eFigure

eFigure 1. Participant Flow in the OARS Trial

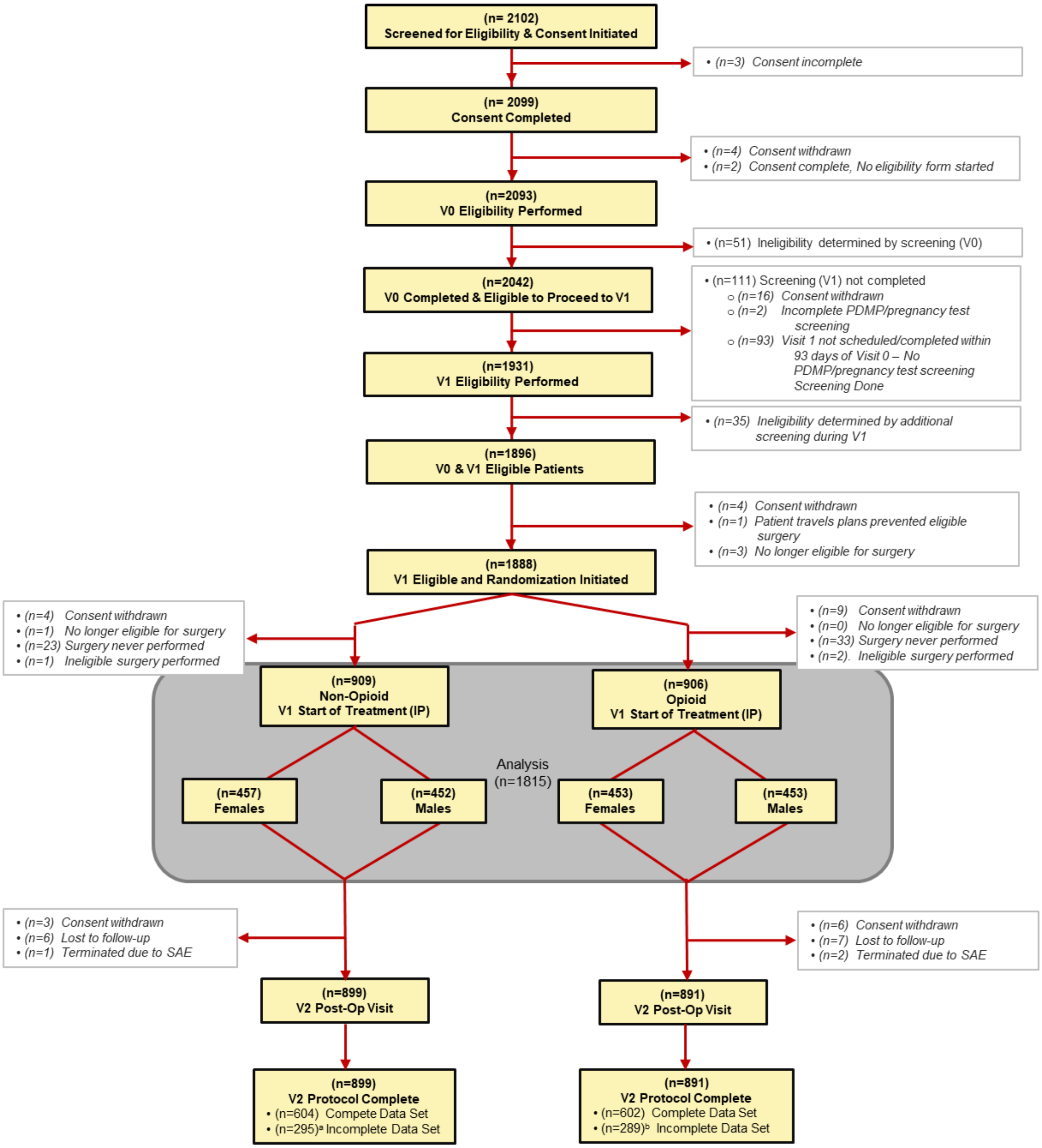

© 2025 Fredericks-Younger J et al. *JAMA Network Open*.
