# Supplementary material for: Analgesic Differences in Males and Females After Third Molar Surgery: A Subgroup Analysis of the OARS Randomized Clinical Trial
Source: JAMA Netw Open. 2025 Nov 6;8(11):e2542467. doi: 10.1001/jamanetworkopen.2025.42467 (PMC12593115; doi:10.1001/jamanetworkopen.2025.42467)
Supplement: Supplement 2. — Trial Protocol and SAP [file jamanetwopen-e2542467-s002.pdf]

National Institute of Dental and Craniofacial Research

Tool Summary Sheet:

NIDCR Interventional Protocol Template

Revision History:

Version Number	Date	Summary of Revisions Made:
0.12	22Sept2019	Contains recommended NIDCR changes section 2.
0.14	31Oct2019	Contains recommended NIDCR changes sections ….
0.18	24Nov2019	Contains additions by Shou-en Lu: randomization procedures (5.4.1), interim analysis (12.3 and 12.3.2), hypotheses (12.1), power and sample size (12.2) and final data analysis (12.4)
0.24	15Jan2020	Contains recommended NIDCR changes: AE/UPs (9.1, 9.1.2, 9.1.3, 9.2) and recommendations from Paul: abbreviations, protocol violations and deviations (16.6)
0.25	17Jan2020	Contains revised ibuprofen dosing: 400mg capsule and addition of parental permissions form
0.31adults.tracked	2March2020	Protocol change to reflect adult subjects only
0.32	19Mar2020	Contains recommendations from Paul Desjardins
0.33	22Mar2020	Contains Shou-En's edits to Aim 3 analysis
0.35	26Mar2020	Contains recommendations from FDA (6.3, 9.5)
0.36	2April2020	References updated and updated consent (FDA recs.) included as Appendix C
0.37	16April2020	Contains updates to FDA reporting requirements (9.4.3) and rectified Table of Contents
0.38	16June2020	Contains recommended NIDCR changes<br>• Clarified language regarding rescue medication and when a subject is discontinued from the study yet data collection continues until the end<br>• Clarified reporting
0.41	28August2020	All track changes approved
0.42	4September2020	Accepted Statistical language updates by Shou-En Lu
1.0	14September2020	sIRB approved
1.01	30October2020	Updated study product tracking system to REDCap(6.4, 14, 14.1, 14.4, 16.2)<br>Contains recommendations from DSMB related to exclusion criteria (5.2)<br>Comprehensive review
2.0	10March2021	Updated protocol deviation reporting (16.5)<br>Updated rescue medication regimen (6.3, 6.6, 7.6)<br>Added protocol in the event participant uses all study product and still requires pain intervention (6.3)
3.0	6April2021	Updated protocol to address incomplete Visit 0 (7.1)<br>Updated protocol to replace manual reminders with automated reminders for eDiary completion (5.3, 7.3, 14.2)
3.01	16June2021	Updated protocol in the event participant uses all study product and still requires pain intervention (6.3)<br>Updated pd/pv reporting to align with NIDCR and MOP (9.1)<br>Updated reporting language of AEs to align with Section 9.4.2 (9.5)

RUTGERS UNIVERSITY
Office for Research
APPROVED
IRB ID: Pro2020002299
APPROVAL: 1/8/2025
EXPIRATION: 1/7/2026

**Number**	**Date**	**Summary of Revisions Made:**
3.02	08July2021	Updated Section 15.3 to allow for electronic consent copy to be given to subject
4.0	08July2021	Published for IRB modification request submission
4.01	15July2021	Updated rescue medication regimen (6.3, 6.6)<br>Updated protocol deviations workflow related to use of exparel or other long lasting local anesthetics (6.10, 6.11, 8.1, 16.5)<br>Corrected Table to align with PDMP query 186 days =/- 14 days from Visit 1 (7.7)<br>Updated SAE reporting to FDA (9.1, 9.4.3)
5.0	30July2021	Published for IRB modification request submission
5.01	25August2021	Updated protocol to address incomplete Visit 1 forms (7.1)
6.0	25August2021	Published for IRB modification request submission
6.01	12October2021	Added additional NDC option for Acetaminophen (Appendix B)
6.02	13October2021	Up to 2400 subjects will be consented to reach the target of 1800 participants receiving IP
7.0	15October2021	Published for IRB modification request submission
7.01	15February2021	QM Process- Annual Protocol Review<br>Clarified how outcomes data was collected (3.1)<br>Clarified electronic or phone messages for intermediate contacts (7.3)<br>Clarified NOMI data storage in OARS EDC project in REDCap (13)<br>Extended subject completion review to every 14 days (14.3)<br>Removed inaccurate storage locations for QM documents (14.5)<br>Removed reference to Spanish translated documents (15.3)
8.0	15March2022	Published for IRB modification request submission
9.0	29June 2023	Updated protocol to address shipment of remaining used and unused study IP (6.4)
10.0	01March 2024	Corrected spelling<br>Updated number of required campaigns from three to four (6.4)<br>Updated statistical analysis plan<br>Updated references

RUTGERS UNIVERSITY
Office for Research

APPROVED
IRB ID: Pro2020002299
APPROVAL: 1/8/2025
EXPIRATION: 1/7/2026

Opioid Analgesia Reduction Study (OARS)

NIDCR Protocol Number: 19-037-E

NIDCR Grant Number: 3DE028860

Principal Investigator: Cecile A. Feldman, DMD, MBA

Grantee Institution: Rutgers University, School of Dental Medicine

NIDCR Program Official: Dena Fischer, DDS, MSD, MS

NIDCR Medical Monitor: Kevin McBryde, MD

FDA Approval: IND 148217

Draft or Version Number: Vr. 10.0

March 1, 2024

RUTGERS UNIVERSITY
Office for Research
APPROVED
IRB ID: Pro2020002299
APPROVAL: 1/8/2025
EXPIRATION: 1/7/2026

STATEMENT OF COMPLIANCE

The study will be conducted in accordance with the International Council for Harmonization guidelines for Good Clinical Practice (GCP) (ICH E6) and the Code of Federal Regulations on the Protection of Human Subjects (45 CFR Part 46). National Institutes of Health (NIH)-funded investigators and clinical trial site staff who are responsible for the conduct, management, or oversight of NIH-funded clinical trials have completed Human Subjects Protection and ICH GCP Training.

RUTGERS UNIVERSITY
Office for Research
APPROVED
IRB ID: Pro2020002299
APPROVAL: 1/8/2025
EXPIRATION: 1/7/2026

TABLE OF CONTENTS

PAGE

STATEMENT OF COMPLIANCE I
SIGNATURE PAGE II
TABLE OF CONTENTS III
LIST OF ABBREVIATIONS VI
PROTOCOL SUMMARY VIII
1 KEY ROLES AND CONTACT INFORMATION 11
2 INTRODUCTION: BACKGROUND INFORMATION AND SCIENTIFIC RATIONALE 13
2.1 Background Information 13
2.2 Rationale 14
2.3 Potential Risks and Benefits 15
2.3.1 Potential Risks 15
2.3.2 Potential Benefits 16
3 OBJECTIVES AND OUTCOME MEASURES 17
3.1 Primary 17
3.2 Secondary 18
3.3 Tertiary/Exploratory 19
4 STUDY DESIGN 20
5 STUDY POPULATION 23
5.1 Participant Inclusion Criteria 23
5.2 Participant Exclusion Criteria 23
5.3 Strategies for Recruitment and Retention 23
5.4 Treatment Assignment Procedures 25
5.4.1 Randomization Procedures (if applicable) 25
5.4.2 Masking Procedures (if applicable) 26
5.5 Participant Withdrawal or Discontinuation from Study Procedures/Intervention 26
5.5.1 Reasons for Participant Withdrawal or Discontinuation from Study Procedures/Intervention 26
5.5.2 Handling of Participant Withdrawals from Study or Participant Discontinuation of Study Intervention 26
5.6 Premature Termination or Suspension of Study 27
6 STUDY INTERVENTION 28
6.1 Study Product Description 28
6.1.1 Acquisition 28
6.1.2 Formulation, Packaging, and Labeling 28
6.1.3 Product Storage and Stability 29
6.2 Dosage, Preparation and Administration of Study Product 29
6.3 Modification of Study Product Administration for a Participant 30
6.4 Accountability Procedures for the Study Product 30
6.5 Assessment of Participant Compliance with Study Product Administration 31
6.6 Concomitant Medications/Treatments 32
6.7 Administration of Intervention 32
6.8 Procedures for Training Interventionists and Monitoring Intervention Fidelity 32

RUTGERS UNIVERSITY
Office for Research
APPROVED
IRB ID: Pro2020002299
APPROVAL: 1/8/2025
EXPIRATION: 1/7/2026

6.9 Assessment of Participant Compliance with Study Intervention 32
6.10 Procedures for Training of Clinicians on Procedural Intervention 32
6.11 Assessment of Clinician and/or Participant Compliance with Study Procedural Intervention 33
7 STUDY SCHEDULE 34
7.1 Screening 34
7.2 Enrollment/Baseline 34
7.3 Intermediate Visits 36
7.4 Final Study Visit 37
7.5 Withdrawal Visit 38
7.6 Unscheduled Visit 38
8 STUDY PROCEDURES/EVALUATIONS 40
8.1 Study Procedures/Evaluations 40
9 ASSESSMENT OF SAFETY 42
9.1 Specification of Safety Parameters 42
9.1.1 Unanticipated Problems 43
9.1.2 Adverse Events 44
9.1.3 Serious Adverse Events 44
9.2 Time Period and Frequency for Event Assessment and Follow-Up 44
9.3 Characteristics of an Adverse Event 45
9.3.1 Relationship to Study Intervention 45
9.3.2 Expectedness 46
9.3.3 Severity of Event 46
9.4 Reporting Procedures 46
9.4.1 Unanticipated Problem Reporting 46
9.4.2 Serious Adverse Event Reporting 47
9.4.3 Reporting of Safety Events to FDA 47
9.4.4 Reporting of Pregnancy 48
9.5 Halting Rules 48
10 STUDY OVERSIGHT 50
11 CLINICAL SITE MONITORING 51
12 STATISTICAL CONSIDERATIONS 52
12.1 Study Hypotheses 52
12.2 Sample Size Considerations 52
12.3 Planned Interim Analyses (if applicable) 53
12.3.1 Safety Review 53
12.3.2 Efficacy Review 53
12.4 Final Analysis Plan 53
13 SOURCE DOCUMENTS AND ACCESS TO SOURCE DATA/DOCUMENTS 58
14 QUALITY CONTROL AND QUALITY ASSURANCE 59
15 ETHICS/PROTECTION OF HUMAN SUBJECTS 62
15.1 Ethical Standard 62
15.2 Institutional Review Board 62
15.3 Informed Consent Process 62

RUTGERS UNIVERSITY
Office for Research
APPROVED
IRB ID: Pro2020002299
APPROVAL: 1/8/2025
EXPIRATION: 1/7/2026

15.4 Exclusion of Women, Minorities, and Children (Special Populations) 62
15.5 Subject Confidentiality 62
15.6 Future Use of Stored Specimens and Other Identifiable Data 63
16 DATA HANDLING AND RECORD KEEPING 64
16.1 Data Management Responsibilities 64
16.2 Data Capture Methods 64
16.3 Schedule and Content of Reports 64
16.4 Study Records Retention 65
16.5 Protocol Deviations 66
17 PUBLICATION/DATA SHARING 68
18 LITERATURE REFERENCES 70
APPENDICES 77
APPENDIX A: SCHEDULE OF EVENTS 78
APPENDIX B: MEDICATION INSERTS 79

RUTGERS UNIVERSITY
Office for Research
APPROVED
IRB ID: Pro2020002299
APPROVAL: 1/8/2025
EXPIRATION: 1/7/2026

LIST OF ABBREVIATIONS

AADR	American Association of Dental Research
ADA	American Dental Association
AE	Adverse Event/ Adverse Experience
BPI	Brief Pain Inventory
BSE	Bovine Spongiform Encephalopathy
CFR	Code of Federal Regulations
CSI	Clinical Site Investigator
CIOMS	Council for International Organizations of Medical Sciences
CNS	Central Nervous System
CONSORT	Consolidated Standards of Reporting Trials
CRF	Case Report Form
CRO	Contract Research Organization
DCC	Data Coordinating Center
DHHS	Department of Health and Human Services
DSMB	Data and Safety Monitoring Board
eCRF	Electronic Case Report Form
FDA	Food and Drug Administration
FFR	Federal Financial Report
FWA	Federalwide Assurance
GCP	Good Clinical Practice
HIPAA	Health Insurance Portability and Accountability Act
IADR	International Association of Dental Research
IASP	International Association for the Study of Pain
IB	Investigator's Brochure
ICF	Informed Consent Form
ICH	International Council for Harmonisation
ICMJE	International Committee of Medical Journal Editors
IDE	Investigational Device Exemption
IND	Investigational New Drug Application
IRB	Institutional Review Board
ISM	Independent Safety Monitor
MOP	Manual of Procedures
N	Number (typically refers to participants)
nMAR	Not Missing at Random

RUTGERS UNIVERSITY
Office for Research
APPROVED
IRB ID: Pro2020002299
APPROVAL: 1/8/2025
EXPIRATION: 1/7/2026

NDA	New Drug Application
NDC	National Drug Code
NIDCR	National Institute of Dental and Craniofacial Research, NIH, DHHS
NIH	National Institutes of Health
NRS	Numeric Rating Scale
OARS	Opioid Analgesic Reduction Study
OCTOM	Office of Clinical Trials Operations and Management, NIDCR, NIH
OHRP	Office for Human Research Protections
OMFS	Oral and Maxillofacial Surgery
PDMP	Prescription Drug Monitoring Program
PHI	Protected Health Information
PI	Principal Investigator
PID	Packet Identification Number
PO	Program Official, NIDCR, NIH
PS	Project Scientist, NIDCR, NIH
PSQ	Pain and Sleep three item index
PTSS	Pain Treatment Satisfaction Scale
QA	Quality Assurance
QC	Quality Control
SAE	Serious Adverse Event/Serious Adverse Experience
SDMC	Statistics and Data Management Core
SID	Subject Identification Number
SOP	Standard Operating Procedure
TSE	Transmissible Spongiform Encephalopathies
UP	Unanticipated Problem
US	United States
VAS	Visual Analog Scale
WHO	World Health Organization

RUTGERS UNIVERSITY
Office for Research
APPROVED
IRB ID: Pro2020002299
APPROVAL: 1/8/2025
EXPIRATION: 1/7/2026

PROTOCOL SUMMARY

**Title:**	Opioid Analgesic Reduction Study (OARS)
**Précis:**	To provide health care professionals, including dentists, with the best possible evidence for clinical decision making when deciding upon analgesics for acute post-surgical pain management; a double-blind, stratified randomized clinical trial will be conducted to test the hypothesis that a combination of over-the-counter non-opioid containing analgesics is at least as, if not more, effective (non-inferior) than the most commonly prescribed opioid analgesic. The impacted 3rd molar extraction model will be used due to the predictable severity of the post-operative pain and generalizability of results, as well as the fact that dentists write about one third of opioid prescriptions for adolescents.<br><br>We will conduct a double blind, prospective, stratified, randomized quasi- pragmatic clinical trial using the impacted 3rd molar extraction pain model.
**Objectives and Outcomes:**	**Primary:** The primary objective of this study is to determine whether a ***NON-OPIOID*** combination of over-the-counter analgesics (ibuprofen/acetaminophen) is non-inferior to the most commonly prescribed ***OPIOID*** (hydrocodone/ acetaminophen) combination product, with respect to patient-perceived pain levels and patient satisfaction observed for seven days following 3rd molar extraction, for the relief of acute post-surgical pain.<br><br>**Secondary:** Secondary objectives include (1) determining frequency and magnitude of adverse events, (2) assessing ability to sleep and perform normal daily activities, (3) determining potential diversion by tracking tablets remaining in households and returned at the follow-up visit, and (4) determining whether NON-OPIOID participants receive fewer opioid prescriptions within 6 months after analgesic use.<br><br>**Outcomes**: Primary - Pain level and patient satisfaction; secondary – adverse events, sleep ability, pain interference (daily function), future opioid seeking behavior, potential diversion
**Population:**	Up to two thousand four hundred subjects (2400) will be consented with the intent to provide randomly assigned IP to eighteen hundred (1800) subjects, 900 males and 900 females age 18 years and older, seeking impacted third molar extraction. Fives centers located in Newark, NJ; Chicago, IL; Baltimore, MD; Rochester, NY: and Ann Arbor, MI will enable a racially and ethnically diverse, opioid naive young adult and adult study population.
**Phase or Stage:**	Phase 3
**Number of Sites:**	Five (5)

RUTGERS UNIVERSITY
Office for Research
APPROVED
IRB ID: Pro2020002299
APPROVAL: 1/8/2025
EXPIRATION: 1/7/2026

**Description of Intervention:**	Two orally administered analgesics are being compared: (1) hydrocodone 5 mg/acetaminophen 300 mg and (2) ibuprofen 400 mg/acetaminophen 500 mg over a post-operative period. Participants will be directed to take 1 dose immediately after surgery and then 1 dose every 4-6 hours as needed for pain. Twenty doses will be dispensed. Equal numbers of male and female participants will be enrolled into the two intervention groups.
**Study Duration:**	Forty eight (48) months.
**Subject Participation Duration:**	Subjects will participate in the study during the nine (9 day +/-5 days) post surgical period. At ~6 months following the surgery, the Prescription Drug Monitoring Program (PDMP) will be accessed (if permissible by state PDMP) to ascertain receipt of opioid prescriptions during this period.
**Estimated Time to Complete Enrollment:**	Thirty six (36) months.

RUTGERS UNIVERSITY
Office for Research
APPROVED
IRB ID: Pro2020002299
APPROVAL: 1/8/2025
EXPIRATION: 1/7/2026

Schematic of Study Design:

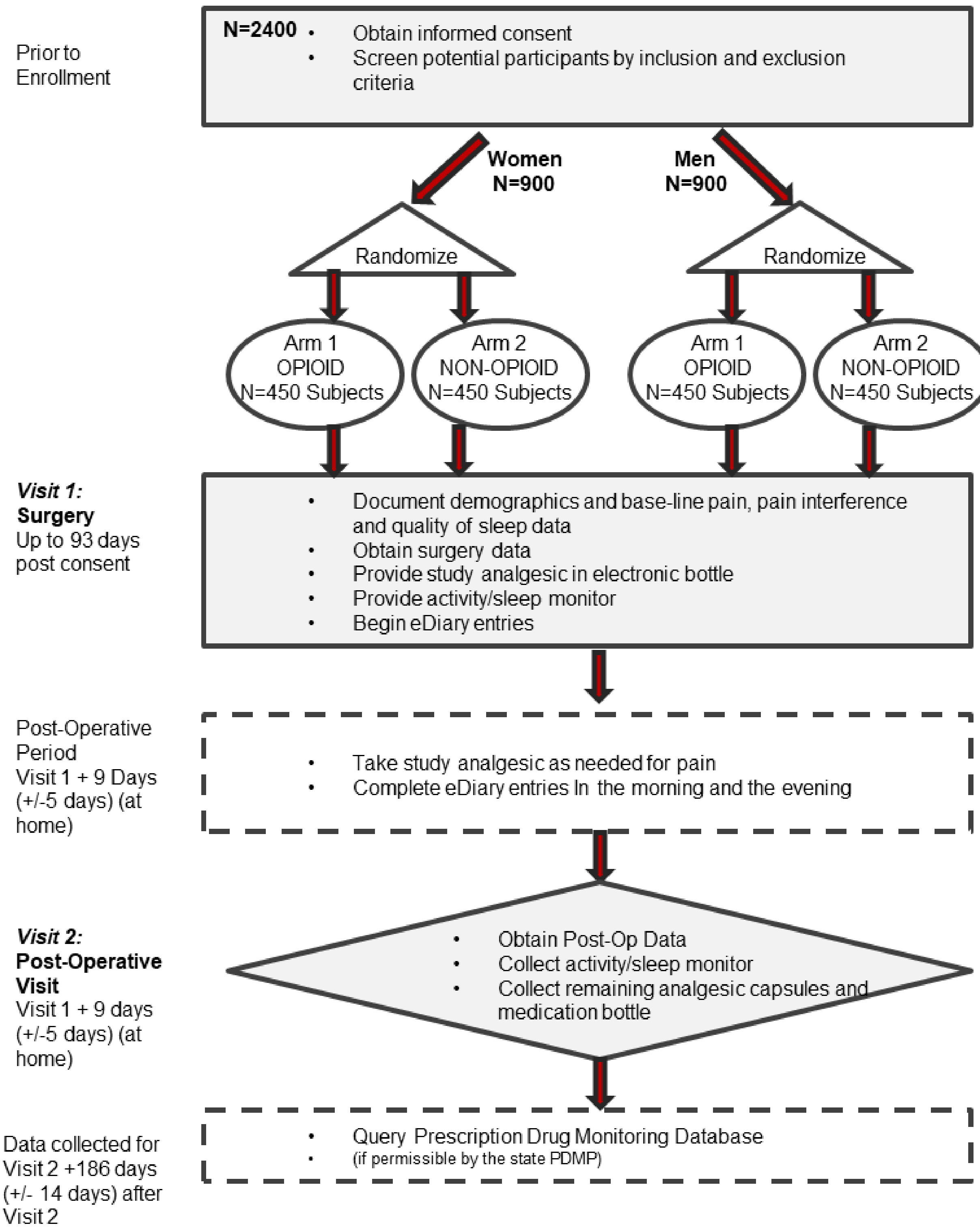

RUTGERS UNIVERSITY
Office for Research
APPROVED
IRB ID: Pro2020002299
APPROVAL: 1/8/2025
EXPIRATION: 1/7/2026

NIH National Institute of Dental and Craniofacial Research

1 KEY ROLES AND CONTACT INFORMATION

Principal Investigator: Cecile A. Feldman, DMD, MBA
Dean and Professor
Rutgers University, School of Dental Medicine
110 Bergen Street
Newark, NJ 07103
Phone: (973) 972-4634
Fax: (973) 972-3689
feldman@rutgers.edu

Medical Monitor: Kevin McBryde, MD
NIH/NIDCR/DER
6701 Democracy Boulevard, Room 638
Bethesda, MD 20892-4878
Phone: 301 594 0170
mcbrydekd@mail.nih.gov

NIDCR Scientist: Dena Fischer, DDS, MSD, MS
National Institute of Dental & Craniofacial Research
6701 Democracy Boulevard, MSC 4878
Bethesda, MD 20892-4878
Phone: (301) 594-4876
dena.fischer@nih.gov

NIDCR Program Official: Darien Weatherspoon, DDS, MPH
National Institute of Dental & Craniofacial Research
6701 Democracy Boulevard, MSC 4878
Bethesda, MD 20892-4878
Phone: (301) 594-5394
darien.weatherspoon@nih.gov

Clinical Site Investigators (Site Directors):

Dr. Hans Malmstrom
Eastman Institute for Oral Health
625 Elmwood Ave.
Rochester, NY 14620
Phone: (585) 273-5087
Fax: (585) 273-1235
hans_malmstrom@urmc.rochester.edu

Dr. Michael Miloro
University of Illinois, College of Dentistry
801 S Paulina St, Room 110 (MC 835)
Chicago, IL 60612
Phone: (312) 996-1052
Fax: (312) 996-5987
mmiloro@uic.edu

Dr. Gary Warburton

RUTGERS UNIVERSITY Office for Research
APPROVED
IRB ID: Pro2020002299
APPROVAL: 1/8/2025
EXPIRATION: 1/7/2026

University of Maryland, School of Dentistry
650 W Baltimore St, Room 1209
Baltimore, MD 21201
Phone: (410) 706-7060
Fax: (410) 706-0891
gwarburton@umaryland.edu

Dr. Brent Ward
University of Michigan, Department of Surgery
1515 E. Hospital Drive
Ann Arbor, MI 48109
Phone: (734) 936-5950
Fax: (734) 936-5941
bward@med.umich.edu

Vincent Ziccardi, DMD, MD
Rutgers University, School of Dental Medicine
110 Bergen St., Room B854
Newark, NJ 07101
Phone: (973) 972-7462
Fax: (973) 972-7322
ziccarvb@sdm.rutgers.edu

Other Key Personnel:

Chief Clinical Officer:

Janine Fredericks-Younger, DMD
Rutgers University, School of Dental Medicine
110 Bergen Street, Room D813
Newark, NJ 07101
Phone: (973) 972-1679
Fax: (973) 972-5040
frederja@sdm.rutgers.edu

Chief* Pharmacology *Officer:

Paul Desjardins, PhD, DMD
Rutgers University, School of Dental Medicine
110 Bergen Street, Room B815
Newark, NJ 07101
Phone: (973) 762-4430
Fax: (908) 477-0056
paul.j.desjardins@gmail.com

Chief Statistical Officer:

Shou-En Lu, PhD
Rutgers University, School of Public Health
683 Hoes Lane
Piscataway, NJ 08854
Phone: (732) 235-5764
Fax: (732) 235-5464
sl1020@sph.rutgers.edu

RUTGERS UNIVERSITY
Office for Research
APPROVED
IRB ID: Pro2020002299
APPROVAL: 1/8/2025
EXPIRATION: 1/7/2026

2 INTRODUCTION: BACKGROUND INFORMATION AND SCIENTIFIC RATIONALE

2.1 Background Information

Description of Health Problem: Dentists are among the leading prescribers of opioid analgesics,[(1)] writing up to 12% of all prescriptions for immediate-release opioids.[(2)] They rank fourth among medical specialties for their opioid prescribing rates, writing 18.5 million opioid prescriptions per year [(3)]. Opioid seeking behaviors have been linked to receipt of initial opioid prescriptions following the common dental procedure of third molar extraction, which 3.5 million young adults undergo annually. Dentists write about 31% of opioid prescriptions for young patients aged 10 to 19[(4)] with about 61% of 14- to 17-year-olds receiving opioid prescriptions following third-molar extractions.[(5)] Dental providers are therefore uniquely positioned to take a leadership role in mitigating the national opioid crisis by reducing the number of opioids prescribed to manage acute pain following common dental procedures.[(6)]

Data from South Carolina's Prescription Drug Monitoring Program (PDMP) for patients prescribed at least one opioid by a dentist (2012-2013) shows that practically all dispensed opioid prescriptions (99.9%; n = 653,650) were for immediate-release opioids and were filled as initial prescriptions (96.2%). Hydrocodone (76.1%) combination products were the opioids most frequently prescribed by dentists. Adolescents and young adults received more than 11% of dentist-prescribed opioids during the same period.[(7)] This finding of opioid-prescribing prevalence for adolescents is consistent with other studies and the assessment of acute opioid prescriptions for youth using PDMP data.[(1, 8)]

We will use the impacted 3rd molar extraction model due to the predictable severity of the post-operative pain and generalizability of results.[(9-13)]

Important Research and Scientific Justification: The third molar extraction model has been widely used to assess interventions to treat acute pain due to its reproducibility and its sensitivity.

> ***Systematic Reviews, Single Dose Vs. Placebo:*** Initial studies evaluating the efficacy of acute pain interventions have compared a single dose of the analgesic to a placebo. In these studies, pain relief was assessed as subjects awaited the onset of pain and then were given a single dose of either the test analgesic (opioid or non-opioid combination) or a placebo. Our team has reviewed the systemic reviews and the clinical trials which were identified.[(14)] These reviews and trials demonstrated the efficacy of both opioid-containing and non-opioid-containing analgesics. They indicated the potential for the non-opioid to manage acute pain, but there were insufficient studies to conclude either superiority or non-inferiority of the non-opioid combination.
>
> ***Systematic Reviews, Acetaminophen and Ibuprofen Combination:*** Systematic review of clinical trials evaluating the acetaminophen and ibuprofen combination studies demonstrated that the combination of acetaminophen and ibuprofen is more effective than either alone.[(15-19, ,] However, these studies do not evaluate whether the non-opioid combination is superior or non-inferior to the typical opioids and opioid-containing combination drugs prescribed for acute post-surgical pain.
>
> ***Trials Comparing NON-OPIOID to OPIOIDS:*** Two completed trials[(20-21,)] and one ongoing trial [(22)] compare acetaminophen/ibuprofen combinations to opioid containing analgesics for acute pain. These are very limited given small sample sizes (n=55 to 175 per group), short duration (2-12 hours), need for significant pain onset before dosing with assigned analgesic, and high dosages of acetaminophen which, if taken over a 7 day period, could surpass the FDA recommended maximum daily dosage. The results of the completed studies suggest that an ibuprofen/acetaminophen combination would be as good if not better than codeine-containing analgesics with no difference between opioid analgesics containing hydrocodone and oxycodone. These studies suggest more

RUTGERS UNIVERSITY
Office for Research
APPROVED
IRB ID: Pro2020002299
APPROVAL: 1/8/2025
EXPIRATION: 1/7/2026

adverse events were experienced with opioid-containing analgesics and did not include any measures of impact on sleep or daily functioning.

Previous Study Weaknesses: Ibuprofen/acetaminophen and opioid/acetaminophen analgesic combinations are commonly prescribed after dental surgery.(19, 23-34) Both have been shown to be more effective than placebos and have been extensively tested as evidenced by several systematic reviews.(15-19, 24, 32-37, ,) Completed studies, however, are limited as they compare the analgesic against a placebo; frequently are single dosage missing the peak post-surgical pain period(38); wait for the onset of pain before taking the analgesic; do not consider gender differences(38-42); do not examine heterogeneity issues; have small sample sizes and do not test for non-inferiority (20-21); use dosages that are either not commercially available or enable patients to easily exceed the FDA recommended maximum dosage; and/or follow patients for just several hours.(35) The array of studies with limited relevance to typical clinical decision making, varying dosages (38), and varying lengths of follow-up makes decisions by both health care providers and patients difficult and subject to individual bias.

Importance of the Study and Any Relevant Treatment Issues or Controversies:
With each opioid prescription, a patient's risk for opioid misuse or abuse increases. Recent studies have shown that exposing young adults to an opioid analgesic increases their risk of future opioid use, with as much as 37% of nonmedical opioid use by high school seniors coming from leftover opioid prescriptions in their or their friend's households.(43) If an alternative analgesic is non-inferior to the most commonly prescribed opioid for impacted third molar extractions, the overall number of opioid prescriptions would be reduced. In turn, with decreased opioid substrate remaining in the community, the number of young adults at risk for opioid addiction via drug diversion has the potential to be significantly reduced.(44, 45)

This trial will address numerous limitations of previous studies and will provide health care professionals, including dentists, with the best possible evidence for clinical-based decision making when deciding upon analgesics for acute post-surgical pain management.

Study Intervention/Study Products:

OPIOID: Hydrocodone represents over 60% of opioid prescriptions for dentistry with codeine being the second most commonly prescribed opioid.(21) Codeine with a morphine milligram equivalent (MME) dose of .15 is less effective than hydrocodone, which has an MME of 1 (i.e., hydrocodone is significantly more powerful than codeine)(46). This trial will utilize hydrocodone 5mg plus acetaminophen 300 mg.

NON-OPIOID: Clinical studies have shown that acetaminophen combined with ibuprofen is more effective than either alone in managing acute post-operative pain.(34, 46-48) The first combination tablet has been approved for the US market by the FDA in March 2020 as *Advil Dual Action (GlaxoSmithKline)*. While the OTC *Advil Dual Action* contains 250mg of ibuprofen and 125mg of acetaminophen and is approved for temporary relief of minor aches and pains, the NON-OPIOID protocol calls for acetaminophen 500 mg (simulating 1 extra strength Tylenol tablet) and ibuprofen 400mg (simulating 2 Advil tablets) which will provide anti-inflammatory effects.

2.2 Rationale

Opioid-related deaths are rising alarmingly in the United States.49) Opioid overdose deaths were five times higher in 2016 than in 1999, an estimated 40% of opioid overdose deaths involved a prescription opioid, and emergency department visits for opioid overdoses in the U.S. increased by 30%.(50) Many patients do not properly secure or dispose of their unused opioid medications, leaving them accessible to others who may initiate or feed an addiction.(51, 52)

RUTGERS UNIVERSITY Office for Research
APPROVED
IRB ID: Pro2020002299
APPROVAL: 1/8/2025
EXPIRATION: 1/7/2026

An estimated 56 million tablets of 5 mg hydrocodone-containing combinations are prescribed after third molar extractions each year in the United States.[15] High school students who receive an opioid prescription are 33% more likely than those who do not receive a prescription to eventually misuse opioids[53] contributing to an upsurge in deaths among 18 to 25-year olds.[54]

Dentists often prescribe opioids to their patients to manage acute post-surgical pain, with hydrocodone/acetaminophen being the most commonly prescribed opioid combination. With an estimated 56 million tablets of hydrocodone prescribed to 3.5 million high school and young adults each year after third molar extractions, it is vitally important to develop the best evidence for acute pain management following dental procedures. This study will compare pain relief and patient satisfaction of an opioid-containing analgesic (hydrocodone with acetaminophen) and a combination of two over-the-counter analgesics (ibuprofen and acetaminophen).

We hypothesize patients will find the ***NON-OPIOID*** to be a non-inferior analgesic for managing acute post-operative pain, and patients will be more satisfied with non-opioids because pain will be properly managed with fewer adverse effects and a better ability to sleep and perform their normal daily functions.

2.3 Potential Risks and Benefits

2.3.1 Potential Risks

This research protocol involves use of medication that have the potential for side effects. Study participants will be informed of the potential short term side effects including, but not limited to:

- upset stomach, nausea, vomiting, loss of appetite, stomach pain;
- bloating, gas, diarrhea, constipation;
- light-headedness, drowsiness, dizziness, feeling tired;
- headache, nervousness;
- mild heartburn;
- mild itching, rash;
- ringing in your ears.
- fainting, weakness
- trouble passing urine, change in the amount of urine.
- sweating;
- bruising, bleeding.
- yellowing of the skin or whites of your eyes.
- tremors, muscle spasms, back pain;
- cold symptoms such as stuffy nose, sneezing, sore throat;
- swelling in your hands or feet.

All adverse events will be recorded as a study outcome. In addition, as part of the informed consent process conducted by the site research coordinator, study participants will be made aware that opioids can be addictive and any unused medications maintained in their household could potentially be misused by other members of their family.

To minimize these risks, all study medications will be provided in small quantities, and subjects will be instructed to take the minimal amount required to control their pain. Subjects will be asked to return any unused pills and an addiction counseling session will be available for anyone obtaining a opioid prescription within the 6 months following surgery. In addition, the SMRxT bottle will electronically monitor the date/time and number of capsules removed.

RUTGERS UNIVERSITY Office for Research
APPROVED
IRB ID: Pro2020002299
APPROVAL: 1/8/2025
EXPIRATION: 1/7/2026

There is a risk of loss of confidentiality for all study participants. Precautions will be in place to minimize this risk, such as collecting only minimal identifying information, using unique study codes for participants, collecting data using encrypted computers, and maintaining electronic data files on a password-protected computer drive, and storing data on encrypted computers or in locked cabinets (located in locked offices). Individual identifier numbers that are linked to participant contact information will be stored separately from the data. Compliance with all IRB regulations concerning data collection, data analysis, data storage, and data destruction will be strictly observed.

For Women: The drugs under study are known to cause birth defects in some animals. It is likely that it may also cause birth defects in people. For this reason, pregnant individuals cannot participate in this study, and potential female participants will be required to take a pregnancy test prior to participation. Women of childbearing age who are sexually active will be asked to use one of the following methods of contraception while taking the study drug:

- contraceptive pill
- intra-uterine device
- condoms
- abstinence

2.3.2 Potential Benefits

Participants are likely to receive an effective pain relieving medication at no cost to them.

RUTGERS UNIVERSITY
Office for Research
APPROVED
IRB ID: Pro2020002299
APPROVAL: 1/8/2025
EXPIRATION: 1/7/2026

3 OBJECTIVES AND OUTCOME MEASURES

3.1 Primary

Objective	Brief Description/Justification of Outcome Measure	Outcome Measured By	Time Frame
**Objective 1 - *Pain management and patient satisfaction:*** To test the hypothesis that a combination of acetaminophen and ibuprofen (NON-OPIOID) is non-inferior to the most commonly prescribed opioid analgesic, hydrocodone and acetaminophen (OPIOID) with respect to pain management and patient satisfaction for approximately seven days following 3rd molar extraction surgery.<br><br>**References**<br><br>***Pain***<br>BPI<br><br>NIH Tool Box: NRS<br>Cook, Dunn 2013<br><br>***Satisfaction***<br>PTSS<br>Evans, Trudeau 2004	***Pain:*** For pain level, questions from the Brief Pain Inventory (BPI) using the Numerical Rating Scale (NRS) was chosen because:<br>• BPI with NRS is widely used and accepted<br>• Reliability and validity for BPI and NRS have been established<br>• BPI with NRS is clinically relevant (patients want to minimize pain experienced after surgery)<br>• BPI with NRS Is a sensitive measure<br>• BPI with NRS will allow for direct comparisons across studies<br><br>***Satisfaction:*** For satisfaction, overall satisfaction questions from the Pain Treatment Satisfaction Scale (PTSS) were chosen because:<br>• While patients want to minimize pain, patients are willing to tradeoff some pain relief to minimize side effects, maintain their ability to sleep, maintain their ability to engage in normal activities and minimize exposure to opioids<br>• PTSS has been shown to be valid and reliable	***Pain:*** NRS will be used on a scale of 0 to 10 where 0=no pain and 10 = worst imaginable pain for average pain level, pain at its worst, pain at its least, pain experiencing now..<br><br>***Satisfaction*** (questions from PTSS):<br>• How satisfied are you with the TIME that it takes your pain medication to work? (scale: 1=very satisfied, 2=satisfied, 3=neither satisfied or dissatisfied, 4=dissatisfied, 5=very dissatisfied)<br>• How satisfied are you with the LEVEL OF AMOUNT of pain relief provided by your pain medication? (scale: 1=very satisfied, 2=satisfied, 3=neither satisfied or dissatisfied, 4=dissatisfied, 5=very dissatisfied)<br>• How satisfied are you with the DURATION of pain relief provided by your pain medication? (scale: 1=very satisfied, 2=satisfied, 3=neither satisfied or dissatisfied, 4=dissatisfied, 5=very dissatisfied)<br>• **Overall**, how satisfied are you with your pain medication? (scale: 1=very satisfied, 2=satisfied, 3=neither satisfied or dissatisfied, 4=dissatisfied, 5=very dissatisfied)<br>• Overall, how does your level of pain relief meet you expectations of pain relief? (scale: 1=greatly exceeds my expectations, 2=somewhat exceeds my expectations, 3=meets my expectations, 4=does not quite meet my expectations, 5=does not meet my expectations at all)<br>• Do you think that your pain medication could be more effective in relieving your pain? (scale: 1=yes, definitely, 2=probably yes, 3=I don't know, 4=probably not, 5=definitely not)<br><br>Participants will report their pain experience in a daily electronic diary and their overall satisfaction on the post-operative survey using a REDCap application developed for electronic phones and tablets.	***Pain:***<br>• Visit 1 (in the last 24 hours)<br>• Each morning days 2 to 8<br>• Each evening days 1 to 7<br>• Visit 2 (in the last 24 hours)<br><br>Missing eDiary entries and survey responses will not be considered protocol deviations. For analysis of outcomes, analysis will include subjects for which a complete data set is available. For example, if a subject did not complete their second morning entry, the subject would be included in the 24 hour analysis but not the 48 or 72 hour analysis<br><br>***Satisfaction:***<br>Satisfaction recorded during post-op visit (Visit 2)

RUTGERS UNIVERSITY
Office for Research
APPROVED
IRB ID: Pro2020002299
APPROVAL: 1/8/2025
EXPIRATION: 1/7/2026

3.2 Secondary

Objective	Brief Description/ Justification of Outcome Measure	Outcome Measured By	Time Frame
**Objective 2 - *Adverse events, pain interference (daily function), and opioid-seeking behavior:*** To test the hypothesis that participants receiving NON-OPIOID will experience fewer and less severe adverse events, experience superior sleep and daily function, and exhibit less opioid seeking behavior than participants receiving OPIOID.<br><br>To test the hypothesis that participants receiving 5 days of opioid-containing analgesics will have tablets/capsules remaining after their acute pain episode has been resolved.<br><br>**References:**<br><br>***Sleep***<br>PSQ-3<br>Ayearst Harsanyi 2012<br><br>***Pain Interference***<br>PEG-3<br>Krebs, Lorenz 2009<br><br>PROMIS Short 6b, BPI and PEG<br>Kean, Monahan 2016<br><br>***Side Effects***<br>PTSS List + 4 others<br>Evans, Trudeau 2004	***Adverse Events:***<br>As medications have side effects, a list of possible adverse events (side effects) related to the intervention has been developed, and participants will be asked if they are experiencing any of them. (This is separate from serious adverse events which will be captured. Serious adverse events will result in a participant being exited from the study. An analysis of serious adverse events will be included in the study analysis.)<br><br>***Sleep Quality:***<br>• Questions from the Pain and Sleep Questionnaire 3 item index (PSQ-3) was selected because it is a validated measure and because of its ease of use for the eDiary on a smart phone.<br>• A question from the PTSS was selected because it provides an overall rating of quality of sleep.<br><br>***Pain Interference (Daily Function):***<br>The PROMIS Short 6b was selected because it is a standard NIH measure of pain interference and can be recorded during the post-operative visit.<br><br>***Future Opioid Seeking Behavior:***<br>The PDMP will be accessed; six months was selected because it is the maximum follow-up time which could be completed within the study time-frame.<br><br>***Potential Diversion:***<br>Participants will be instructed to bring the pill bottle and unused capsules to the follow-up appointment.	***Adverse Events:***<br>• Adverse events include: excessive fatigue or drowsiness, inability to concentrate, nausea, diarrhea, dizziness, constipation, skin rashes, stomach aches, heartburn, vomiting, euphoria, headache, urinary retention and unintentional weight gain with a binary yes/no scale. Self-reported binary response (yes/no) will be ascertained. If yes - was the adverse event bothersome to a minor or major extent?<br>○ For Visit 1, participants will be asked how much were you bothered by … over the last 24 hours.<br>○ At the time of getting up in the morning participants will be asked how much were you bothered with …. during the night<br>○ Right before going to sleep at night, participants will be asked how much they were bothered by … during the day.<br>○ For Visit 2, participants will be asked how much were you bothered by … over the last 24 hours.<br><br>***Sleep Ability:***<br>• From the PSQ-3: a. Last night did you have trouble falling asleep? b. Last night were you awakened by pain during the night? c. Were you awakened by pain this morning? {Binary yes/no scale during the post-operative period; NRS scale where 0=never, 10=always at Visit 1 and 2)<br>• From PTSS: Rating the overall quality of last night's sleep {NRS where 0=excellent and10=very poor}<br>• From Actigraph: Sleep quality will be monitored and data collected<br><br>***Pain Interference:***<br>• PROMIS: During the post-operative period, how much did pain interfere with your day to day activities, work around the home, ability to participate in social activities, enjoyment of life, the things you usually do for fun, enjoyment of social activities, household chores, family life, your ability to concentrate, enjoyment of recreational activities, tasks away from home {scale: 1=not at all, 2=a little bit, 3=somewhat, 4=quite a bit, 5=very much} and How often did pain keep you from socializing with others? {scale=1=never, 2=rarely, 3=sometimes, 4=often, 5=always}<br><br>***Future Opioid Seeking Behavior:***<br>• # of new opioid prescriptions recorded in the Prescription Monitoring Database Program at approximately 6-months following the surgical procedure<br><br>***Potential Diversion:***<br>• # returned capsules at Visit 2 determined by counting the returned capsules or via electronic monitoring device<br>• # unaccounted for capsules at Visit 2 (not recorded as used and not returned)<br><br>Participants will report whether or not they have experienced each adverse event and will rate their sleep ability and pain interference in a daily electronic diary using a REDCap application developed for electronic phones and tablets.	***Adverse Events:***<br>• Visit 1<br>• Each night for days 2-8<br>• Each day for days 1-7<br>• Visit 2<br><br>***Ability to sleep:***<br>• Visit 1<br>• Each morning on days 2 thru 8 and<br>• Visit 2 (in the last 24 hours)<br><br>***Pain Interference:***)<br>• Visit 1,<br>• Each evenings on days 1 through 7<br>• Visit 2<br><br>***Future Drug Seeking Behavior:***<br>• Opioid prescriptions filled within 6 months after visit 2<br><br>***Potential Diversion:***<br>• Visit 2<br><br>*Missing eDiary entries and survey responses will not be considered protocol deviations. Data analysis will be performed on an intent-to-treat basis, using likelihood based statistical methods (e.g., mixed model analysis). To statistically treat missing data, we will perform sensitivity analyses using the multiple imputation procedures[90-92] and explore methods to model the missingness mechanism and apply the methods of selection models[93] or use the pattern-mixture models such as the control-based pattern imputation approach, or the tipping-point approach to handle missing data.[94-96]*

RUTGERS UNIVERSITY Office for Research
APPROVED
IRB ID: Pro2020002299
APPROVAL: 1/8/2025
EXPIRATION: 1/7/2026

3.3 Tertiary/Exploratory

Objective	Brief Description/Justification of Outcome Measure	Outcome Measured By	Time Frame
**Objective 3 (exploratory) - Clinical Protocol/Decision Support Tool:** Develop a model that recommends which combination analgesic to prescribe along with the number of tablets to maximize a patient's overall satisfaction.	***Number and Frequency of Medication Intake:***<br>*Understanding the pattern of medication usage will impact recommendations.*<br><br>All other measures collected for this exploratory objective are collected as part of the primary and secondary objectives. See above.<br><br>Dependent variables include type, number and frequency of medication intake, patient satisfaction.<br><br>Independent variables include number and location of extractions (maxillary vs. mandibular), level of surgical difficulty, surgical time, education level, gender, race and ethnicity, age, BMI and smoking history.	**Number and Frequency of Medication Intake**<br>*A SMRxT medication bottle will be utilized to electronically monitor the number of capsules removed at a given time over the study period*<br><br>For all other measures see above.	**Number and Frequency of Medication Intake**<br>Between the time the first dose is taken post-surgery until Visit 2<br><br>For all other measure see above.

RUTGERS UNIVERSITY
Office for Research
APPROVED
IRB ID: Pro2020002299
APPROVAL: 1/8/2025
EXPIRATION: 1/7/2026

4 STUDY DESIGN

Description: A double blind, prospective, stratified, randomized quasi-pragmatic clinical trial comparing a combination analgesic of hydrocodone 5mg/acetaminophen 300 mg(55-61) (***OPIOID*** -- the most commonly prescribed opioid by dentists)(7, 62-67) to the combination of ibuprofen 400mg/acetaminophen 500mg (***NON-OPIOID*** – the most common over-the-counter analgesics(15-17, 20, 46, 68-75), readily available today in any supermarket or drug store) will be conducted.

The planned dosages were selected because both study product dosages can be safely increased should adequate pain relief not be obtained. Participants will be stratified based upon gender due to differences in prescribing patterns and pain tolerance,(39-42) and will be randomized at a 1:1 ratio. Up to 2400 subjects will be consented. Each arm will provide IP to 450 randomly assigned participants for a total of 1800 participants. The study is designed to evaluate the non-inferiority of ***NON-OPIOID*** in real life practice conditions providing results which can be generalized and applied to routine practice settings described below.

Study Population: The study population will comprise individuals 18 years and older requiring extraction of one or more partial or fully impacted mandibular 3rd molars. Most participants are likely to be young adults, as there is a high prevalence of young adults requiring 3rd molar extractions.

Real Interventions:

Study Product	# of capsules per bottle	Capsule Size	Color
OPIOID			
Hydrocodone 5mg/ Acetaminophen 300 mg	20	AA	Brown
Placebo	20	00	White
NON-OPIOID			
Ibuprofen 400mg	20	AA	Brown
Acetaminophen 500mg	20	00	White

Medication will be used the way it is prescribed in practice today. Study product will be in capsule form. Capsules are to be taken orally, 1 dose (1 brown capsule from bottle 1 and 1 white capsule from bottle 2) at a time. Participants are to take 1 dose immediately after surgery and then 1 dose every 4-6 hours as needed for pain not to exceed 6 doses within a 24 hour period. In the event pain management is insufficient, they must call the on-call surgeon who may instruct the participant to take up to 2 additional doses.

Unless the study participant is unaccompanied, they will take their first analgesic dosage before leaving the dental office. By taking this first dosage before local anesthesia wears off, the patient can “get ahead of the pain,” which makes pain management easier through the entire post-operative period.

Important Outcomes: Our primary study outcome is pain experience and satisfaction with managing pain. We will also examine several secondary outcomes (adverse events, ability to sleep, pain interference - ability to conduct daily activities), and the number of capsules which can be diverted.

Single or Multicenter: This is a multi-center trial involving 5 sites: Rutgers University, University of Illinois at Chicago, University of Maryland, University of Michigan, and University of Rochester. Potential participants will be recruited from the dental school clinics at each of these clinical sites.

Phase of Trial: **3**

Number of Study Groups/Arms: Four (4) - MALE/OPIOID, MALE/NON-OPIOID, FEMALE/OPIOID, and FEMALE/NON-OPIOID

RUTGERS UNIVERSITY
Office for Research
APPROVED
IRB ID: Pro2020002299
APPROVAL: 1/8/2025
EXPIRATION: 1/7/2026

Expected Duration of Subject Participation: Participants will participate in the study for the time between their impacted 3rd molar extraction procedure until their post-operative visit which normally occurs approximately 1 week later (9 days +/- 5 days). Following state laws and regulations, the PDMP will be retrospectively queried 6 months post-surgery to ascertain information about any opioid containing prescriptions that may have been filled during this post-surgery time period.

Sequence of Procedures and Duration of Study Period: The Research Coordinator will obtain consent with the potential participant followed by a detailed review of inclusion and exclusion criteria. The 3rd molar surgery appointment will occur within 3 months (93) days after obtaining consent, and randomization will occur prior to the 3rd molar extraction procedure. Randomization will be performed within each site, stratified by gender to ensure that randomization procedures will apply equally to men and women. Subgroup analyses will be performed.

The intervention will be administered following the surgery, and participants will be followed for 9 (-/+5) days post-surgery. This represents the time between surgery and the post-operative visit, covering the entire acute pain phase which is normally 7-10 days long. At Visit 1 (Surgery Visit), participants will complete a PreOp Subject Questionnaire addressing pain intensity, pain interference, sleep, and overall satisfaction. During the post-operative period, participants will be asked to complete a diary with morning and evening entries. When the participant returns for his/her post-operative visit, generally 7 to 10 days after surgery, he/she will return the pill bottle with unused medication, the actigraph, and a survey will be administered, addressing pain intensity, pain interference, sleep, and overall satisfaction. If permitted by the state PDMP, a PDMP query will be made 6 months later to ascertain information about any opioid prescriptions filled during this post-surgical time period.

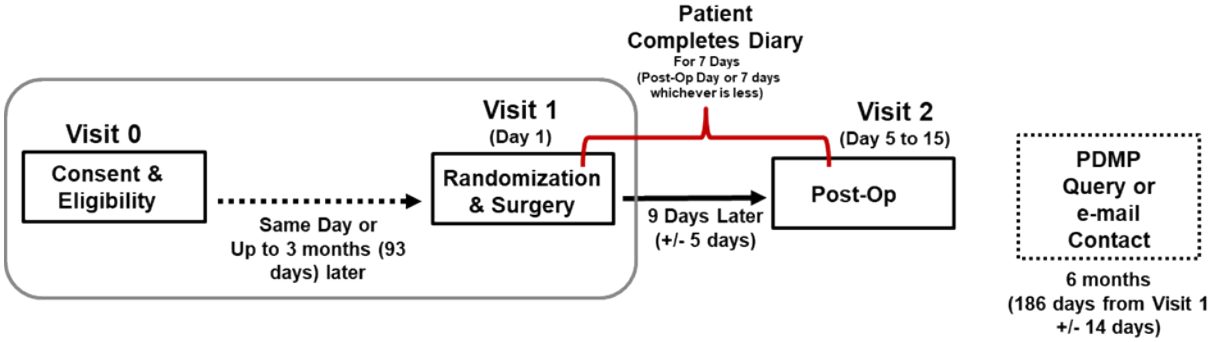

Planned Variation in Intervention Dose or Schedule: Participants will take a single dose of study medication after surgery and then will be provided instructions to take an additional dose every 4-6 hours as needed for pain. If a dosage is not effective within an hour, a participant can take an additional dose, up to 6 total doses per day. In the event pain management remains insufficient, the subject will be instructed to call the on-call surgeon. The on-call surgeon will asses the situation and may instruct the participant to take up to 2 additional doses or determine if rescue medication or an emergency visit is required. If hospitalization is required, the participant will be withdrawn from the study. If study product is ineffective and rescue medication is prescribed, the study intervention will be discontinued, and the participant will continue with data collection procedures.

Methods for Data Collection

- REDCap forms will be administered by study personnel using an iPad tablet or desktop computer. Data collected include eligibility criteria; demographics; pre-operative patient-reported data (i.e. pre-operative pain, pre-operative sleep ability, and pre-operative pain interference), surgical procedure data (i.e. Surgical Treatment Case Report, including: teeth extraction, type of impaction, extraction difficulty, length of surgery, pharmaceuticals used) and post-operative patient-reported data (i.e.

RUTGERS UNIVERSITY Office for Research
APPROVED
IRB ID: Pro2020002299
APPROVAL: 1/8/2025
EXPIRATION: 1/7/2026

post-operative pain, post-operative sleep ability, post-operative pain interference, complications, overall satisfaction).

- REDCap surveys for eDiary entries. For these entries, a text message or email will be sent to a participant's cell phone in the morning and evening during the post-operative period with the link to the REDCap surveys. Participants will click on the link to access the surveys and make their eDiary entries directly through their cell phones.
- SMRxT Electronic Medication Bottles will record the date and time each capsule is removed from the bottle. This will provide the number of capsules removed each day and provide the total number of capsules removed enabling the determination of number of capsules available for diversion at the end of the post-operative period.
- Actigraph Sleep/Activity Monitor will record active calories, total sleep minutes and deep sleep minutes.

RUTGERS UNIVERSITY
Office for Research
APPROVED
IRB ID: Pro2020002299
APPROVAL: 1/8/2025
EXPIRATION: 1/7/2026

5 STUDY POPULATION

5.1 Participant Inclusion Criteria

An individual must meet all of the following criteria to be eligible to participate in the study:

- Be able to understand the informed consent.
- Provide signed and dated informed consent form
- Be able to understand all directions for data gathering instruments in English
- Be willing and able to comply with all study procedures, including having a smart phone, and be available for the duration of the study
- Planning to undergo extraction of one or more partial or fully impacted mandibular 3rd molars
- Be 18 years or older
- Be in good general health as evidenced by medical history
- Women must agree to use one of the following methods of contraception while participating in this study:
    - contraceptive pill
    - intra-uterine device
    - condoms
    - abstinence

5.2 Participant Exclusion Criteria

Participants who self-report the following history will be excluded from participating:

- History of gastrointestinal bleeding and/or peptic ulcer
- History of renal disease (excluding kidney stones)
- History of hepatic disease
- History of cardiovascular disease (MI or stroke with the past 6 months)
- History of bleeding disorder
- History of respiratory depression
- Any prior respiratory effect of an opioid or other anesthetic drugs that required respiratory support postoperatively
- Active or untreated asthma
- History of known allegoric reaction to ibuprofen, acetaminophen, hydrocodone, and/or anesthesia
- Currently taking any of the following medications:
    - CYP3A4 inhibitor, such as macrolide antibiotics (e.g., erythromycin), azole-antifungal agents (e.g., ketoconazole), and protease inhibitors (e.g., ritonavir), which may increase plasma concentrations of hydrocodone bitartrate and acetaminophen and prolong opioid adverse reactions, and which may cause potentially fatal respiratory depression
    - CNS depressants (including Benzodiazepines).
- Consumes 3 or more alcoholic drinks every day and/or has a history of alcoholism
- History of drug or alcohol abuse (excludes marijuana use)
- Family history of drug or alcohol abuse in a first degree relative
- Has had no more than one opioid prescription filled within the past 12 months
- Currently pregnant or lactating

Participants would also be excluded due to any additional criteria that would place the individual at increased risk or preclude the individual's full compliance with or completion of the study which includes:

- Prior participation in this study
- Inability or refusal to provide informed consent

5.3 Strategies for Recruitment and Retention

Target Sample Size: 2400 subjects will be consented to provide IP to 1800 total subjects: 900 Female, 900 Male

Target Sample Size by Gender, Race, Ethnicity, and Age: Study population will be drawn from outpatient adult patients seeking extraction of impacted 3rd molars in the following communities: Ann Arbor, MI;

RUTGERS UNIVERSITY
Office for Research
APPROVED
IRB ID: Pro2020002299
APPROVAL: 1/8/2025
EXPIRATION: 1/7/2026

Baltimore, MD; Chicago, IL; Newark, NJ and Rochester, NY. While most participants seeking third molar extract will be young adults, adults of any age can be enrolled.

The population will represent the typical clinical population with respect to gender, race and ethnicity. For example, the patient population at Rutgers School of Dental Medicine is diverse as the dental school clinics provide care for a large proportion of underserved minorities and economically disadvantaged citizens from varied cultural backgrounds, many of whom are non-English speakers with varying levels of education. In AY2018-2019, 54.0% of patients were female, 13.6% were children (0-12), 17.6% were geriatric (65+), 51.2% utilized Medicaid, 14.8% were Black/African American and 13.4% were Hispanic/Latino. 50.6% did not provide a race/ethnicity.

Study Population: The study population will be drawn from outpatient dental clinics located at the five university sites – Rutgers University, University of Illinois, University of Michigan, University of Maryland and University of Rochester. Each site sees over 350 patients requiring extraction of impacted 3rd molars a year. Target enrollment will vary by site, with a goal of providing IP to 1800 participants across the five clinical sites.

Inclusion of Women and Minorities and Individuals of All Ages:

Women: A stratified random sample design with stratification on gender will be used to ensure that one half of enrolled participants will be female (n=900) and one half will be male (n=900).

Minorities: Sites selected are all located in diverse communities and serve a diverse patient population. It is anticipated that at least 32% will be African American and 17% will be Hispanic or Latino.

Recruitment Strategies: Each clinical site will recruit study participants as they report to the oral and maxillofacial surgery clinic for 3rd molar extraction consultation or surgery appointments. Each site will have study staff who will review the clinic schedule at least each week for potential study candidates. When patients report to the clinic for a 3rd molar extraction consult or procedure, study staff will ascertain interest in study participation if at least one partial bony mandibular 3rd molar extraction is planned. Sites may advertise for the study by word of mouth and/or advertising through flyers, social media, e-mail and/or ads in local/college newspapers.

Projected Enrollment and Completion Per Year

Site	# Patients with Impactions Seen in 2017	Estimated Enrollment per Year (assuming 40% eligibility)*	Estimated Completion per Year (assuming 80% completion)	Estimated Completion Over 3 Year Period
Illinois	1781	350	280	840
Maryland	428	171	137	411
Michigan	415	166	133	398
Rochester	432	173	138	415
Rutgers	377	151	121	362
**TOTAL**	**3433**	**1011**	**809**	**2426**

*=capped at 350 to ensure diversity of sites

Should a site not reach its randomization target of at least 125 over each 12 month period, consideration will be given to discontinuing enrollment at that site and adding another in its place.

Retention: In order to minimize loss of study participants and/or incomplete data collection, the following activities will take place:

- An electronic message will be sent to the participant each morning and each evening with a link to the e-diary and reminder to complete their e-diary entry.
- If the eDiary entry is not made within 2 hours, an automatic text message reminder is sent up to two times

Compensation and Scheduled Payments: Participants will receive a $125 payment card (credit, debit, or gift card(s)) at the end of their post-operative visit for participating if they report for their post-operative visit

RUTGERS UNIVERSITY Office for Research
APPROVED
IRB ID: Pro2020002299
APPROVAL: 1/8/2025
EXPIRATION: 1/7/2026

within 2 weeks (by day 15) of their impacted third molar extraction, return all study materials (medication bottle with remaining tablets and activity tracker) eDiary entries, and complete the pre-operative survey and the post-operative survey.

Additional Plans to Minimize Loss of Follow-up and Missing Data: The following will minimize loss of follow-up and/or missing data:

- REDCap survey data fields set to "required". Surveys will not be able to be saved without responses provided to all questions.
- REDCAP validation rules on data fields to limit responses to valid responses
- Cellular connection to electronic medication bottle enabling real time capture of capsule removal from the bottle and number of capsules removed
- Electronic capture of sleep and daily activity data from an activity and sleep monitor

5.4 Treatment Assignment Procedures

Randomization of assigned analgesic (OPIOID vs. NON-OPIOID) will be stratified by gender at each site. The Statistics and Data Management Core (SDMC) at Rutgers will generate 10 randomization lists, two for each site, with one list being for females and a second list generated for males. The randomization schema will result in sequential assignment to a treatment group (OPIOID or NON-OPIOID) for each consented participant presenting for surgery. For example, if the generated randomization list for females at Rutgers starts with O, N, N O, then the first female reporting for surgery at Rutgers would be assigned to the OPIOID group, the second female would be assigned to the NON-OPIOID group, the third female would be assigned to the NON-OPIOID group fourth female assigned to the OPIOID group.

The Clinical Protocol Coordinating Core at Rutgers School of Dental Medicine will be responsible for putting together a study package of materials for each participant at each site. The packages will be assembled by the Clinical Protocol Core Research Coordinator. Study product will be transferred one-to-one from manufacturer bottles labeled with NDC codes to new SMRxT bottles and placed into the participant packet bag. Each participant packet bag will be labeled with a study participant ID number only. The label will not contain the group assignment nor the name of the specific enclosed analgesic. These study participant packages will be organized in the randomization order generated by the SDMC and shipped to the sites, ensuring that site personnel are blinded.

Participant packages will be provided to enrolled participants in the order that they present for surgery.

Analgesic group assignment will be traceable by the statistician and members of the Clinical Protocol Coordinating Core, but no site personnel will know group assignment unless unblinding for a specific participant is required due to an emergency.

5.4.1 Randomization Procedures

Participants will be randomized to either the opioid or non-opioid analgesic group at the 1:1 ratio, stratified by gender at each site. Site-specific pre-determined random number sequences, with a block of 4 containing 2 opioid and 2 non-opioid assignments in a random order, will be generated by staff at Data Management Statistical Analysis Core (DMSA) using REDCap (or R software). The randomization code will be generated and labels will be created during the preparation phase when the treatment packets are prepared so that each packet will be prepared and labeled with the packet identification number according to the randomization sequence. Complete randomization code for each site will be stored in REDCap and only the DMSA and CPCC staff have access to it. The CPC Core will monitor the number of male and female packets distributed to participants per site per month to monitor enrollment/ randomization per group. The

RUTGERS UNIVERSITY
Office for Research
APPROVED
IRB ID: Pro2020002299
APPROVAL: 1/8/2025
EXPIRATION: 1/7/2026

randomization code will be broken and merged with other measurement data, including the primary and secondary outcome data collected by the e-questionnaires/e-diaries, only at the final data analysis stage.

5.4.2 Masking Procedures

Study analgesics will be provided in 2 different size/color capsules. Existing FDA approved caplets will be placed into a capsules and then filled with powder fill. Capsule 1 for OPIOID and NON-OPIOID will manufactured with a similar weight to the extent possible. Similarly, capsule 2 of OPIOID and NON-OPIOID will be manufactured with a similar weight to the extent possible.

Capsule Number	OPIOID Content	NON-OPIOID Content	Quantity for a Dose	Total Dispensed	Capsule Size	Color
**1**	Hydrocodone 5mg / Acetaminophen 300 mg	Ibuprofen 400 mg	1	20	AA	Brown
**2**	Placebo	Acetaminophen 500 mg	1	20	00	White

The study Instructions for the taking the analgesic will be the same for both the opioid and non-opioid cohorts, ensuring the patient, surgeon and site nurse coordinator will be blinded.

5.5 Participant Withdrawal or Discontinuation from Study Procedures/Intervention

5.5.1 Reasons for Participant Withdrawal or Discontinuation from Study Procedures/Intervention

Participants are free to withdraw from participation in the study at any time upon request.

An investigator may discontinue an individual’s participation in an intervention or withdraw an individual from the study if:

- Exparel is used on the participant during the extraction procedure
- The participant has a serious adverse event requiring hospitalization.
- Any clinical adverse event (AE), laboratory abnormality, or other medical condition or situation occurs such that continued participation in the study would not be in the best interest of the participant.
- The participant meets an exclusion criterion (either newly developed or not previously recognized) that precludes further study participation.

5.5.2 Handling of Participant Withdrawals from Study or Participant Discontinuation of Study Intervention

Participants may withdraw participation consent at any time, either verbally or in writing. Participants who further withdraw their consent for the use of data already collected must do so in writing. The Research Coordinator will interview the participant and document the withdrawal of consent using the Consent Withdrawn Received Form in the REDCap system. Documentation includes the date, the reason for participant withdrawal, as well as, the upload of any written withdrawal request. Upon withdrawal, all study interventions would cease, however, the participant would be offered continued post-operative care, including return for his/her post-operative visit, as part of the normal standard of care.

The surgeon may discontinue intervention for a participant due to an adverse event or unanticipated problem. After consultation with the surgeon, the Site Research Coordinator will complete the appropriate REDCap documentation (Adverse Event Form, Serious Adverse Event Form, or Unanticipated Problems Form) based on the situation. Appropriate reporting would follow. For the participant, despite the discontinuation of the intervention, he/she would continue with data collection procedures, including return for

RUTGERS UNIVERSITY
Office for Research
APPROVED
IRB ID: Pro2020002299
APPROVAL: 1/8/2025
EXPIRATION: 1/7/2026

his/her post-operative visit (study Visit 2), and would be offered continued post-operative care as part of the normal standard of care.

Up to 2400 subjects will be consented to reach the target of 1800 participants receiving IP. This assumes a 25% attrition rate between consent and date of surgery, and an 18% participant attrition rate which is defined as participants who do not complete Visit 2. If the attrition rate is less than 18%, no additional participants will be recruited. If the attrition rate is equal to or greater than18%, additional participants will be recruited to attain 1480 participants who have completed the protocol.

5.6 Premature Termination or Suspension of Study

This study may be suspended or prematurely terminated if there is sufficient reasonable cause. Written notification, documenting the reason for study suspension or termination, will be provided by the suspending or terminating party to Dr. Cecile Feldman and the funding agency (NIDCR). The principal investigator will also promptly inform the IRB and NIDCR and will provide the reason(s) for the termination or suspension.

Circumstances that may warrant termination include, but are not limited to:

- Determination of unexpected, significant, or unacceptable risk to participants.
- Insufficient adherence to protocol requirements.
- Data that are not sufficiently complete and/or evaluable.
- Determination of futility.

RUTGERS UNIVERSITY
Office for Research
APPROVED
IRB ID: Pro2020002299
APPROVAL: 1/8/2025
EXPIRATION: 1/7/2026

6 STUDY INTERVENTION

6.1 Study Product Description

Two combination analgesics will be used: Hydrocodone 5 mg/acetaminophen 300 mg and ibuprofen 400 mg/acetaminophen 500 mg. Hydrocodone 5 mg/acetaminophen 300 mg is available as a tablet in the US. Ibuprofen 400 mg/acetaminophen 500 mg is not available in the US as a combination analgesic at that dosage. Thus, FDA-approved hydrocodone 5mg/acetaminophen 300 mg, ibuprofen 400 mg and acetaminophen 500 mg caplets will each be obtained and then over-encapsulated. These FDA approved analgesics will be used according to label and capsules used for over encapsulation will be bovine spongiform encephalopathy/transmissible spongiform encephalopathy (BSE/TSE) free. Appendix B contains the package inserts for hydrocodone 5mg/acetaminophen 300 mg, ibuprofen 400 mg and acetaminophen 500mg. The specific caplet forms have been selected because of their ability to be over-encapsulated.

6.1.1 Acquisition

The study product will be acquired through University of Maryland Pharmacy, a pharmaceutical manufacturer which fabricates medications for clinical trials. Existing FDA approved hydrocodone 5mg/ acetaminophen 300mg, ibuprofen 400 mg and acetaminophen 500 mg will be acquired.

6.1.2 Formulation, Packaging, and Labeling

Preparation of Study Product by the Manufacturer
The manufacturer will prepare study product by over-encapsulation of each of the three existing FDA approved medications: hydrocodone 5mg/acetaminophen 300mg, ibuprofen 400 mg and acetaminophen 500 mg. Capsules used for over encapsulation will be bovine spongiform encephalopathy/transmissible spongiform encephalopathy (BSE/TSE) free. These FDA approved analgesics will be used according to label.

Study Product	# of capsules per bottle	Capsule Size	Color
OPIOID			
Hydrocodone 5mg/ Acetaminophen 300 mg	20	AA	Brown
Placebo	20	00	White
NON-OPIOID			
Ibuprofen 400mg	20	AA	Brown
Acetaminophen 500mg	20	00	White

Study product will be shipped to the Rutgers Clinical Protocol Coordinating Core from the manufacturer in bottles of 20 capsules. Each bottle will be clearly marked with a unique bottle number, the contents (drug), quantity, batch/lot number, manufacture date, and expiration or retest date. Individual bottles will be electronically logged into REDCap upon receipt.

Preparation of Study Packets by the Clinical Core
When preparing a packet of materials for a participant, there will be a one to one medication transfer from the manufacturer's bottle to the SMRxT bottles. Specifically, for OPIOID participant, twenty (20) capsules of hydrocodone/acetaminophen will be transferred to a SMRxT bottle and twenty (20) capsules of the placebo will be transferred to a second SMRxT bottle. For a NON-OPIOID participant, twenty (20) capsules of acetaminophen will be transferred to a SMRxT bottle and twenty (20) of ibuprofen will be transferred to a second SMRxT bottle.

RUTGERS UNIVERSITY
Office for Research
APPROVED
IRB ID: Pro2020002299
APPROVAL: 1/8/2025
EXPIRATION: 1/7/2026

SMRxT bottles are child proof, light protected bottles which weigh the capsules contained within the bottle. Each time a pill is removed or replaced and the bottle placed on a flat service, the number of capsules removed is transmitted via cellular service to the SMRxT secure cloud. This enables the research staff to track usage of capsules in real time.

A prescription label will be affixed to each SMRxT bottles which includes instructions to be followed by the participant.

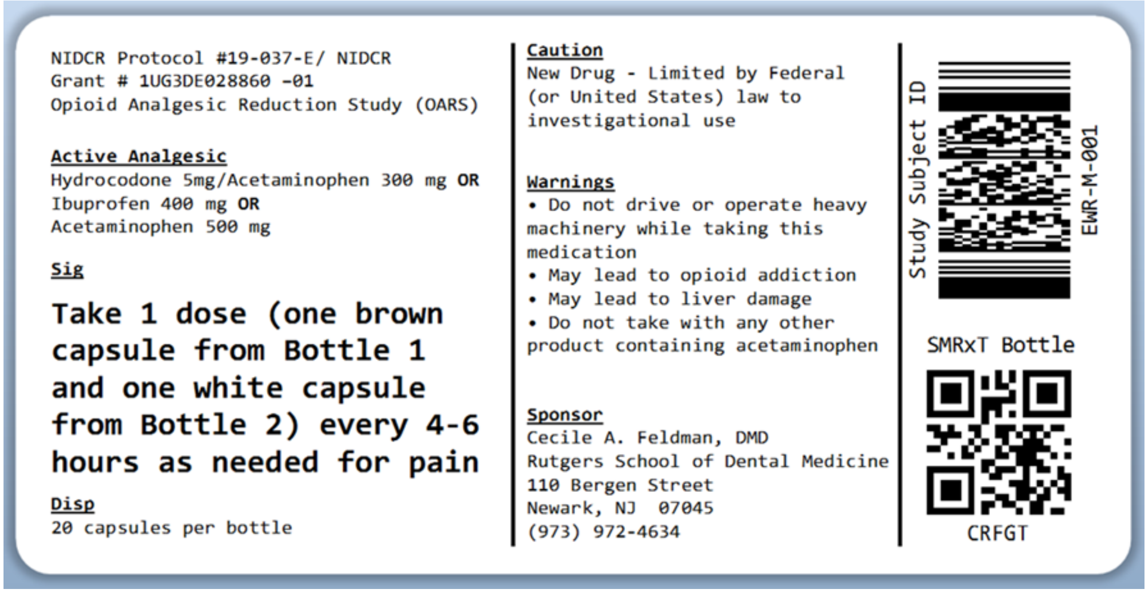

NIDCR Protocol #19-037-E/ NIDCR
Grant # 1UG3DE028860 -01
Opioid Analgesic Reduction Study (OARS)

Active Analgesic
Hydrocodone 5mg/Acetaminophen 300 mg **OR**
Ibuprofen 400 mg **OR**
Acetaminophen 500 mg

Sig

Take 1 dose (one brown capsule from Bottle 1 and one white capsule from Bottle 2) every 4-6 hours as needed for pain

Disp
20 capsules per bottle

Caution
New Drug - Limited by Federal (or United States) law to investigational use

Warnings
- Do not drive or operate heavy machinery while taking this medication
- May lead to opioid addiction
- May lead to liver damage
- Do not take with any other product containing acetaminophen

Sponsor
Cecile A. Feldman, DMD
Rutgers School of Dental Medicine
110 Bergen Street
Newark, NJ 07045
(973) 972-4634

Study Subject ID
EWR-M-001

SMRxT Bottle

CRFGT

6.1.3 Product Storage and Stability

Study product can be stored under normal temperature and humidity conditions. Study product does not need to be refrigerated.

At the CPC Core, all non-opioid study product will be stored in locked cabinets with the opioids stored in a pharmaceutical safe until ready for shipment to the clinical sites. At the clinical sites, all study product, which is now blinded, will be stored in locked pharmaceutical safes until being made ready for distribution to participants. The night before distribution to a participant, the SMRxT bottles need to be charged. Charging stations have been assembled in locked ventilated metal cabinets. Each SMRxT bottle will have a label with a bar code which will be used to track study product.

6.2 Dosage, Preparation and Administration of Study Product

Study product will be in capsule form. Capsules are to be taken orally, 1 dose (1 brown capsule from bottle 1 and 1 white capsule from bottle 2) at a time. Participants are to take 1 dose immediately after surgery and then 1 dose every 4-6 hours as needed for pain.

Two capsules will be required for each dosage.

- For OPIOID GROUP:
  - One capsule will contain hydrocodone 5mg/acetaminophen 300 mg encapsulated in a brown AA capsule.
  - One capsule will contain the placebo formed into a white 00 capsule.
- For NON-OPIOID GROUP:
  - One capsule will contain ibuprofen 400 mg encapsulated into a brown AA capsule.
  - One capsule will contain acetaminophen 500 mg encapsulated into a white 00 capsule.

RUTGERS UNIVERSITY Office for Research
APPROVED
IRB ID: Pro2020002299
APPROVAL: 1/8/2025
EXPIRATION: 1/7/2026

6.3 Modification of Study Product Administration for a Participant

If insufficient pain relief is obtained, participants can take up to 6 total doses within a 24 hour period. Should adverse events be too bothersome or if participant experiences any serious adverse events, participants will be instructed to call the provided emergency number. If pain relief is inadequate, the surgeon will advise an 2 additional doses of study medication to manage pain. If there is still inadequate pain relief, patients will be prescribed rescue medication, 5mg oxycodone q6h prn pain, while the subject continues to take study drug (max 12 tabs). If rescue is inadequate, a second rescue would be initiated, and additional medication would be prescribed at the clinician's discretion.

OPIOID

	Hydrocodone	Acetaminophen	OTC Ibuprofen
1 Study Dose + 5 OTC	5 mg	300 mg	2000 mg
2 Study Doses + 4 OTC	10 mg	600 mg	1600 mg
3 Study Doses + 3 OTC	15 mg	900 mg	1200 mg
4 Study Doses + 2 OTC	20 mg	1200 mg	800 mg
5 Study Doses + 1 OTC	25 mg	1500 mg	400 mg
6 Study Doses + 0 OTC	30 mg	1800 mg	0 mg
**7 Study Doses + 0 OTC**	**35 mg**	**2100 mg**	**0 mg**
**8 Study Doses + 0 OTC**	**40 mg**	**2400 mg**	**0 mg**

NON-OPIOID

	Acetaminophen	Ibuprofen	OTC Ibuprofen	Total Ibuprofen
1 Study Dose + 5 OTC	500 mg	400 mg	2000 mg	2400 mg
2 Study Doses + 4 OTC	1000 mg	800 mg	1600 mg	2400 mg
3 Study Doses + 3 OTC	1500 mg	1200 mg	1200 mg	2400 mg
4 Study Doses + 2 OTC	2000 mg	1600 mg	800 mg	2400 mg
5 Study Doses + 1 OTC	2500 mg	2000 mg	400 mg	2400 mg
6 Study Doses + 0 OTC	3000 mg	2400 mg	0 mg	2400 mg
**7 Study Doses + 0 OTC**	**3500 mg**	**2800 mg**	**0 mg**	**2800 mg**
**8 Study Doses + 0 OTC**	**4000 mg**	**3200 mg**	**0 mg**	**3200 mg**

*Study doses 7 and/or 8 would occur only after a call to the surgeon and if the surgeon recommends taking the extra dosages.

Study participants will be informed that there are risks associated with each of the study medications, including the fact that the study medication contains acetaminophen and that there are risks associated with taking acetaminophen in large quantities (over 4,000mg per day). A number of over-the-counter (OTC) medications contain acetaminophen and participants will be made aware of the additive effect of taking other acetaminophen-containing medications along with the study medication. Patients will also be allowed to use OTC Ibuprofen 400 mg in place of Study Analgesic.

If the participant utilizes all of the study product provided and still requires pain intervention, additional medication recommendations would be at the clinician's discretion.

6.4 Accountability Procedures for the Study Product

Study Product Distribution: Hydrocodone/Acetaminophen, acetaminophen, and ibuprofen will be shipped from the University of Maryland, School of Pharmacy, Applied Pharmaceuticals Lab to the Rutgers School of Dental Medicine Core in bottles of 20 capsules. Bottles will be clearly labeled with its contents. As a means to monitor varying expiration and retest dates of the medication, study product will be manufactured and shipped to Rutgers School of Dental Medicine in up to four campaigns. Expiration and retest dates will be tracked in REDCap..

Each bottle of study product, each SMRxT bottle and each Actigraph will contain bar code labels with unique identifying codes on them. REDCap will be used to electronically track the location of the study medication in the SMRxT bottles and Actigraph throughout the study. All materials for a study participant will be packaged at the Rutgers CPC Core which will also contain a bar code label with a unique package ID. Materials will be

RUTGERS UNIVERSITY
Office for Research
APPROVED
IRB ID: Pro2020002299
APPROVAL: 1/8/2025
EXPIRATION: 1/7/2026

scanned out of the CPC Core, scanned into clinical sites, and scanned when provided to and returned from participants.

Clinical sites will place an order when subject packages are needed Subject packages will be shipped to each site in denominations of 8. When packaging a shipment, the study packet being packed will be scanned into REDCap to register the package being sent to a clinical site. Shipping in denominations of 8 as the randomization process ensures that 4 of the 8 packages must contain OPIOID. It is anticipated that shipments of 8 packets will be sent two to three times a month to each site.

Shipments will be made using UPS which enables the tracking of shipments and signing of packages to acknowledge receipt.

Each site will receive the study material, scan the package ID label and all of the contents of each subject package into REDCap, and store in the site pharmaceutical safe until needed. This scanning informs the REDCap system that the subject package has been received by the site.

Further details can be found in the Manual of Procedures, section 5.

Frequency of Product Distribution: It is anticipated that every two weeks, study participant packages will be shipped to the clinical sites from the Rutgers CPC Core.

Amount of Product Shipped: Product will be shipped to ensure that each site sufficient OPIOID and sufficient NON-OPIOID packets on hand at any point in time.

Product and Device Tracking Procedures: To ensure all product and devices are tracked, every item used will contain a bar code label. As the product and devices are shipped form the Rutgers CPC Core to the site and then back to the Rutgers CPC Core, bar code readers will be used to record items being shipped and record items being received.

Plans for Return of Unused Product: Each participant will return their unused medication, SMRxT bottles and Actigraph during their post-operative visit (Visit 2). The Site Clinical Research Coordinator will store the participant materials in their pharmaceutical safe until the site is ready to ship the materials back to the Rutgers CPC. It is anticipated that used materials will be shipped to the Rutgers CPC Core twice a month in quantities of eight.* When packing and unpacking the materials, each item will be scanned into REDCap, thus enabling the tracking of materials as they are being transferred. Unused study product will be destroyed by Rutgers University at least twice a year according the University and DEA protocols.

*At the conclusion of enrollment and after the last subject kit is collected at the post-operative exam, site personnel will ship all remaining used and unused study product to the CPC Core. This final shipment will be in a quantity of eight or less.

6.5 Assessment of Participant Compliance with Study Product Administration

Other than the initial dose, study participants are not required to take any study medication. The number of capsules taken along with the date and time the capsules are taken will be recorded by Nomi, the software associated with the SMRxT medication bottle.

Participant use of the study product will be determined in 3 ways:

- The SMRxT with Nomi software will record the time/date study product is removed from the SMRxT bottle.
- Morning and evening eDiary entries will ask study participants how many study product capsules they took during the night and during the day.

RUTGERS UNIVERSITY
Office for Research
APPROVED
IRB ID: Pro2020002299
APPROVAL: 1/8/2025
EXPIRATION: 1/7/2026

- The number of capsules returned to the site research coordinator during the participant's post-operative visit.

6.6 Concomitant Medications/Treatments

Concomitant Medications: Participants taking CYP3A4 inhibitors or CNS depressants are not eligible to enroll. This information will be collected at the time of eligibility. Participants can be enrolled if taking analgesics or antibiotics that are not CYP3A4 inhibitors or CNS depressants. Surgeons are permitted to use their normal clinical protocols which may include general anesthesia, conscious sedation, local anesthesia (except Exparel and other long lasting anesthetics), antibiotics, and anti-inflammatory agents. The Surgical Case Report will record the types of pharmaceuticals used.

Rescue Medication: Participants will be told that if they are not getting sufficient pain relief taking 4 doses per day, they can take an additional 2 doses up to a maximum of 6 doses per day. Should this still not be sufficient, the participant is to call the on-call number to be put into contact with the on-call surgeon. If pain relief is inadequate, the surgeon may advise an 2 additional doses of study medication to manage pain. If there is still inadequate pain relief, patients will be prescribed rescue medication, 5mg oxycodone q6h prn pain (max 12 tabs), while the subject continues to take study medication. If rescue is inadequate, a second rescue would be initiated and additional medication would be prescribed at the clinician's discretion.

If a study participant takes rescue medication, he/she may continue with data collection procedures, including study Visit 2.

6.7 Administration of Intervention

Participants will be given their assigned analgesic (study product) after surgery in their study material package. Each study package will contain 20 doses distributed in two bottles. Participants will be asked to take the first dose before leaving the office as long as they are accompanied by another individual. If they are not accompanied, participants will be asked to take their first dose when they get home.

6.8 Procedures for Training Interventionists and Monitoring Intervention Fidelity

Study analgesic will be self-administered. The Site Research Coordinators will be trained to provide consistent instructions across sites and participants pertaining to administration of study analgesic, utilization of study materials, and acquisition of study data. A Participant Instructions flip chart will be used to ensure consistency of participant instructions. All training of research coordinators will be conducted by either the CPC Core Research Coordinator or the CPC Core Chief. Training will take place either in-person or via WebEx or similar videoconferencing technology.

6.9 Assessment of Participant Compliance with Study Intervention

As this is a quasi-pragmatic clinical trial, participants are able to determine when and how much of the study analgesic to use. Assessment of participant compliance is described in Section 6.5.

6.10 Procedures for Training of Clinicians on Procedural Intervention

As the study intervention is study product, the 3rd molar surgical procedures that will occur are not the study intervention and will not be standardized with the exception of avoiding the use of Exparel, a long lasting local anesthetic. Use of Exparel or other long lasting anesthetics will cause a participant to be withdrawn from the study. While the surgeons are not study investigators and will not be collecting or recording data for the study, they will receive general training so they know the purpose of the trial and will be told that Exparel should not be used on enrolled participants. (None of the clinical sites use Exparel in their surgical protocols.).

RUTGERS UNIVERSITY
Office for Research
APPROVED
IRB ID: Pro2020002299
APPROVAL: 1/8/2025
EXPIRATION: 1/7/2026

6.11 Assessment of Clinician and/or Participant Compliance with Study Procedural Intervention

Clinician Compliance: The REDCap Surgical Case Report records pharmaceuticals used during the surgical procedure. On a weekly basis, the Quality Control Analyst will review all surgical case report forms for the week to ensure compliance with not using Exparel or other long lasting anesthetics. Should a clinician be found using Exparel, the site director will be informed so the clinician can be counseled

Patient Compliance: REDCap will send electronic messages to study participants each morning and each evening reminding the study participant to make their REDCap eDiary entries. Each [business] day, the Site Research Coordinators will be able to view patient compliance with making eDiary entries via the REDCap dashboard. In addition, the Site Directors and the Site Research Coordinators will be alerted via email by Nomi if more than 8 capsules be taken from a subject's SMRxT medication bottles within 24 hours. The Site Clinical Research Coordinator will then contact the participant via phone to determine if the participant should be referred to an on-call surgeon.

RUTGERS UNIVERSITY
Office for Research

APPROVED
IRB ID: Pro2020002299
APPROVAL: 1/8/2025
EXPIRATION: 1/7/2026

7 STUDY SCHEDULE

7.1 Screening: Eligibility/Consent Visit. (Visit 0)

Visit 0: Study personnel will approach potential participants who present to the participating clinics for either consultation or treatment for a full or partial bony 3rd molar extraction procedure of at least one mandibular third molar. If the potential participant is interested in participating in the study, consent will be obtained. Study personnel will then review inclusion and exclusion criteria via the Participant Eligibility Form. At the end of this visit, the participant will be scheduled for surgery.

In the event that Visit 0 is incomplete (i.e. consent complete and eligibility incomplete or not started), the participant will be reconsented.

Eligibility & Consent Visit Time Period	Purpose	What will Occur	Standard of Care or Study Procedure	Responsible Parties	Time Window for Study Visits
**Visit 0**	Obtain informed consent and review eligibility criteria	Obtain informed consent	Study Procedure	In-Person meeting and discussion between Site Research Coordinator and potential participant	Day 0
		Consult to review medical history and determine type of extraction	Standard of Care	In-person contact with patient as part of Standard of care	
		Complete eligibility form	Study Procedure	In-Person meeting and discussion between Site Research Coordinator and potential participant	

7.2 Enrollment/Surgery: Visit 1, Day 1 (0-93 days after visit 0)

Visit 1: At the start of the enrollment/surgery visit, eligibility is reconfirmed. The state's PDMP will be queried to ascertain whether the subject has had one or more opioid prescriptions within the past six months. One or more opioid prescriptions within the past six months will render the participant ineligible. In addition, any female participant will be required to take a urine pregnancy test to ensure eligibility at this time. A positive pregnancy test will render the female participant ineligible. Therefore, if all inclusion criteria have been met and no exclusion criteria present, the participant will be enrolled in the study.

Enrollment is noted by a participant ID being assigned and recorded in the REDCAP ID Link *and SMS Test* form. (Note: Visit 0 and Visit 1 can take place the same day.) Since study packages will be available at each site in randomized order, assignment of a participant ID number is consistent with randomization.

Prior to surgery, the pre-operative questionnaire will be administered by the Site Clinical Research Coordinator. Participants will be taught how to complete their eDiary entries, shown the SMRxT bottle and instruction will be provided on proper use of the study medication and bottle. Participants will also be

RUTGERS UNIVERSITY Office for Research
APPROVED
IRB ID: Pro2020002299
APPROVAL: 1/8/2025
EXPIRATION: 1/7/2026

provided the Actigraph activity monitor. They will be shown how to use the monitor and instructed on wearing the monitor for 72 hours.

Participants will then be given their SMRxT bottles with their assigned study analgesic and asked to demonstrate the use of the bottles. Immediately after surgery, participants will take their first dose (1 brown capsule and 1 white capsule) in the dental office if accompanied by an escort. Otherwise, if unaccompanied, the participant will be instructed to take the first dose of study medication upon arriving home. Following surgery, the Site Clinical Research Coordinator will complete the Surgical Case Report based upon information provided by the oral and maxillofacial surgeon who performed the surgery.

Incomplete Visit 1

If surgery is postponed after randomization, the subject kit would be retrieved and stored in the medication safe at the clinical site until the new surgery date/time.

- If consent is still valid (within 93 days), the following forms would be required on the new day of surgery using the existing Subject ID in REDCap:
    - Gender and Pregnancy form
    - Pre-Op Subject Questionnaire
    - Surgical Case Report
- If the consent is invalid (> 93 days after initial consent), the subject kit would be returned to the CPC core. A returning patient would be required to complete Visit 0 and Visit 1 in its entirety with a new REDCap Subject ID.

Enrollment and Surgical Visit Time Period	Purpose	What will Occur	Standard of Care or Study Procedure	Contacts with Participants	Time Window for Study Visits
**Visit 1 (Day 1)**<br>Up to 93 days after Screening Visit	To collect patient-reported pre-operative information, educate patient on pain medication (intervention) administration and study procedures, perform surgery and record surgery information, and instruct patient to take initial analgesic dosage	Participant will be assigned a ID number (randomized)	Study Procedure	N/A	Up to 93 days after consent is obtained
		Pre-operative survey will be administered	Study Procedure	In-person meeting between Site Research Coordinator and participant	
		Patient will receive study package and education on pain medication, study protocol, wearing of activity monitor and how to complete the	Study Procedure	In-person meeting between Site Research Coordinator and	

RUTGERS UNIVERSITY
Office for Research
APPROVED
IRB ID: Pro2020002299
APPROVAL: 1/8/2025
EXPIRATION: 1/7/2026

		eDiary		participant	
		3rd molar extractions performed<br><br>After surgery, patient will take 1st dosage if accompanied or will be instructed to take 1st dosage at home if unaccompanied	Standard of Care	Clinical appointment with the surgeon	
		Surgical Case Report completed	Study Procedure	Research Coordinator interviews surgeon	

7.3 Intermediate Contacts: Days 1-10 +/- 5 Days

During this period, participants will take study analgesic as directed, as needed for pain. Participants will receive electronic message and/or phone call notifications, reminding them to complete the eDiary. Specifically, participants will receive messages via text or email each morning and evening containing the link for their eDiary entry and serving as a reminder to complete these entries.

Intermediate Time Period	Purpose	What will Occur	Standard of Care or Study Procedure	Contacts with Participants	Time Window for Study Visits
**Post-Operative Period**<br>Between Visit 1 and Visit 2	To collect information from patient on pain, sleep ability, pain interference, and adverse effects	Morning Text/ Email	Study Procedure	Automated message from REDCap	Day 1 to Day 10 +/- 5 days
		Resending of original morning text/email up to two times as a reminder	Study Procedure	Automated message from REDCap	
		Morning eDiary Entry	Study Procedure	Self-Completed Survey	
		Evening Text/ Email	Study Procedure	Automated message from REDCap	
		Resending of original evening text/email up to two	Study Procedure	Automated message from	

RUTGERS UNIVERSITY
Office for Research
APPROVED
IRB ID: Pro2020002299
APPROVAL: 1/8/2025
EXPIRATION: 1/7/2026

		times as a reminder		REDCap	
		Evening eDiary Entry	Study Procedure	Self-Completed Survey	
		Run *Nomi Pill Count* report in REDCap<br>Look for both bottles to have recorded activity	Study Procedure	Phone call by site research coordinator to patient as needed	
	To ensure both SMRxT bottles are appropriately tracking medication				Day 2

7.4 Final Study Visit – Post-Operative Visit: (Visit 2, Day 10 +/- 5 Days)

<u>**Visit 2:**</u> After the post-operative examination, complications will be recorded by the site research coordinator by interviewing the surgeon person the Post-Operative Case Report Questionnaire Form. The site research coordinator will collect all study materials from the participant and administer the Post-Operative Questionnaire. The questionnaire will be completed to document pain, pain interference, sleep quality, adverse events, and satisfaction over the last 24 hour period. If the participant returns all study materials (SMRxT bottles, unused medication, and activity monitor), the participant will be paid $125. Participants will acknowledge receipt of the payment will be made by the participant affixing their signature at the end of the Post-Operative Questionnaire.

Final Visit (Post-Operative Visit) Time Period	Purpose	What will Occur	Standard of Care or Study Procedure	Contacts with Participants	Time Window for Study Visits
**Visit 2**<br>Day 10 +/- 5 days	To perform post-operative exam and collect post-operative data	Post-operative survey will be administered	Study Procedure	In-person meeting between Site Research Coordinator and patient	Day 10 +/- 5 days
		Post-Op Exam will be performed	Standard of Care	Clinical appointment with the surgeon	
		Site Research Coordinator will interview surgeon to complete Post-Operative Case Report	Study Procedure	N/A	
		Patient returns all	Study	In-person meeting with	

RUTGERS UNIVERSITY
Office for Research
APPROVED
IRB ID: Pro2020002299
APPROVAL: 1/8/2025
EXPIRATION: 1/7/2026

		study materials	Procedure	Research Coordinator	
		Participant is provided payment card	Study Procedure	In-person meeting with Research Coordinator	
		Record of payment entry made into REDCap Post-Operative Survey	Study Procedure	In-person meeting with Research Coordinator	

7.5 Withdrawal Visit or Withdrawal Notification

The study protocol does not include a withdrawal visit. No additional information will be collected if a participant withdraws consent or is terminated from the study by an investigator.

7.6 Unscheduled Visit

Unscheduled visits may occur if the subject exhibits a complication as a result of the extraction procedure or study intervention. Should an unscheduled visit occur, an Adverse Event Form will be filed. Data to be collected include: date of visit, reason for visit, treatment rendered at the visit and any prescription medication prescribed, including rescue analgesics. If the surgeon determines that the subject should be withdrawn from the study due to complications, the subject will be withdrawn from the study and an entry will be made in REDCap. Data to be entered include date and reason for termination. If the surgeon determines that study drug should be discontinued, an entry will be made in in the *Adverse Event* form. An unscheduled visit or discontinuation of study drug will not automatically result in the subject being withdrawn from the study. For example, if the participant requires rescue medication, the participant will continue with data collection procedures.

Intermediate Visit Time Period	Purpose	What will Occur	Standard of Care or Study Procedure	Contacts with Subjects	Time Window for Study Visits
**Post-Operative Period**<br>Between Visit 1 and Visit 2	Address any adverse events experienced by the subject	Surgeon will examine patient and perform any necessary treatment	Standard of Care	Surgeon	Day 1 to Day 10 +/- 5 days
		Entry made in Adverse Event Form by the Site Research Coordinator	Study Procedure	N/A	
		If patient is terminated from study, entry made in the *Site Director Subject Close-out Sign-off* form by the Site Director	Study Procedure	N/A	
		If adverse events causes the study	Study Procedure	Surgeon	

RUTGERS UNIVERSITY
Office for Research
APPROVED
IRB ID: Pro2020002299
APPROVAL: 1/8/2025
EXPIRATION: 1/7/2026

		intervention to be discontinued, entry made on the Adverse Event Form			

7.7 PDMP Query & Follow-up

Each state regulates PDMP access for its providers. In accordance with state laws and regulations, a PDMP inquiry will be conducted approximately 6 months after Visit 1 (if permitted by state PDMP). The PDMP inquiry will be made to ascertain information about opioid prescriptions obtained and filled by the subject during the 6-month post-surgical period. Within 2 weeks of receiving information that an opioid prescription was obtained during the 6 month period, the site research coordinator will attempt to contact the subject up to 3 times within a 2 week period to offer consultation with an addiction counselor. Should the site coordinator not be able to reach the subject by phone after three attempts, an e-mail letter will be sent to the subject with the addiction counselor contact information and a recommendation to pursue counseling. As the counseling session is confidential, no information regarding the session will be obtained from the counselor and no information will be recorded. If state regulations prohibit a post-surgery PDMP query at six months, an email will be send to all subjects from that locale 6 months after surgery to remind them of the availability of an addiction counseling session if needed.

PDMP Inquiry and Follow-up Time Period	Purpose	What will Occur	Standard of Care or Study Procedure	Contacts with Subjects	Time Window for
**~6 months after Visit 1**<br>**2-week period**	To ascertain whether subject obtained and filled an opioid prescription after post-operative period	PDMP will be queried by site research coordinator (if permitted by state PDMP)	Study Procedure	N/A	~186 days after Visit 1 +/- 2 weeks
		If opioid prescription is found to have been filled, 3 attempts will be made to contact patient by phone to refer to offer an addiction counseling session and if unsuccessful an e-mail letter will be sent	Study Procedure	Site Research Coordinator	Within 2 weeks of a positive PDMP Inquiry
		If state regulations prohibit a post surgery PDMP query at six months, an email will be send to all subjects from that locale to remind them of the availability of an addiction counselor referral if needed.	Study Procedure	Site Research Coordinator	~186 days after Visit 1 +/- 2 weeks

RUTGERS UNIVERSITY
Office for Research
APPROVED
IRB ID: Pro2020002299
APPROVAL: 1/8/2025
EXPIRATION: 1/7/2026

8 STUDY PROCEDURES/EVALUATIONS

8.1 Study Procedures/Evaluations

The study protocol calls for the following procedures to be completed. Detailed procedures can be found in the Manual of Procedures, Section 6.

Procedure and Evaluations	Purpose	Procedure Completed as Part of Study	How Completed
• **Complete Consent**	Complete informed consent	Yes	**Visit 0:** Study investigators meet with subject
**Eligibility Determination**<br>• Medical History<br>• Contraindications<br>Concomitant medications	Determine whether subject meets criteria to participate	Yes	**Visit 0:** Research personnel recording inclusion/exclusion criteria based upon subject interview
**PDMP Query**	Ascertain subject's PDMP history	Yes	**Visit 1:** Research personnel look up subject's PDMP history in the state PDMP data base
**Pregnancy Verification**	Ensure females are not pregnant	Yes	**Visit 1:** Site Research Coordinator enters results of urine pregnancy test into REDCap form
**Subject Reported Base-line Measures**<br>• Pain<br>• Pain Interference<br>• Sleep<br>• Adverse Events	Collect outcome measures	Yes	**Visit 1:** Data entered into REDCap via form by Site Research Coordinator who interviews subject before survey (Visit 1)
**Surgical, Case Reports***	Collect information on surgical visit	Yes<br>(Surgical procedure is part of standard of care though completing case report via surgeon interview is part of study procedure)	**Visit 1:** Site Research Coordinator interview of surgeon
**Self-Reported Outcome Data Collection**<br>• Pain<br>• Pain Interference<br>• Sleep<br>• Adverse Events	Collect outcome measures	Yes	**Time Between Visit 1 and Visit 2:** Data entered into REDCap directly by subject via REDCap surveys which comprise the eDiary
**Post Operative Case Reports**	Collect information on post-operative visit	Yes<br>(Post-Operative visit is part of standard of care though completing case report via surgeon interview is part of study procedure)	**Visit 2:** Site Research Coordinator interview of surgeon
**Subject Reported Outcome Measure**<br>• Pain<br>• Pain Interference<br>• Sleep<br>• Adverse Events<br>• Satisfaction	Collect outcome measures	Yes	**Visit 2:** Data entered into REDCap via forms by Site Research Coordinator who interviews subject after post-operative visit (Visit 2)
**Study Product Usage Data Capture**	Collect information on the date/time capsules are removed from the bottles (proxy for when study	Yes	Time between Visit 1 and Visit 2: Date/time/dosage recorded into Nomi website via cellular connection and then downloaded into REDCap after Visit 2

RUTGERS UNIVERSITY
Office for Research
APPROVED
IRB ID: Pro2020002299
APPROVAL: 1/8/2025
EXPIRATION: 1/7/2026

	product is taken)		
**Activity/Sleep Data Capture**	Collect information on a subjects activity and sleep	Yes	After Visit 2: Data downloaded from Actigraph into REDcap
Adverse Event form	Collect information on intermediate office visits or ED visits	Yes<br>(Intermediate visit is part of standard of care though completing case report via interview of surgeon is part of study procedure)	Time between Visit 1 and Visit 2: Site Research Coordinator interview of surgeon
**PDMP Query**	Provide counseling referral if subject as additional opioid prescriptions filled	Yes<br>(PDMP query is being performed to offer patient additional services, though recording of PDMP query is part of study procedure)	**186 Days +/- 14 days after Visit 1:** Site Research Coordinator query of subject PDM according to state PDMP guidelines

* Use of Exparel, Marcaine and other long lasting local anesthetics will be collected via the REDCap Surgical Case Report Questionnaire. Use of Exparel, Marcaine and other long lasting local anesthetics will be documented via the Site Research Coordinator or the Site Quality Control Analyst in REDCap. Data collected on any subject receiving Exparel will be removed before the database is locked.

RUTGERS UNIVERSITY Office for Research
APPROVED
IRB ID: Pro2020002299
APPROVAL: 1/8/2025
EXPIRATION: 1/7/2026

9 ASSESSMENT OF SAFETY

9.1 Specification of Safety Parameters

Safety data will be collected through solicitation of AEs captured through daily eDiaries, safety events spontaneously reported by the participant or observed by the investigators, and safety events recorded at or between the study visits when there is communication between study staff and the research participant (see figure in Section 9.2).

Event	Definition	Form	Who completes REDCap form	Time Frame For Reporting to IRB	Additional Reports to
**Adverse Events**	Any untoward or unfavorable medical occurrence in a human subject, including any abnormal sign (for example, abnormal physical exam or laboratory finding), symptom, or disease, temporally associated with the subject's participation in the research, whether or not considered related to the subject's participation in the research	Adverse Events Form	If clinic or ED visit Is not required - subjects self-report via eDiary entries;<br><br>If visit/ED visit is required - reported via Adverse Event form by clinical research coordinator	5 business days from the date of discovery	**FDA** (within 15 days after reporting determination from 1) serious, unexpected suspected adverse reactions, or (2) a clinically important increase in the rate of a serious suspected adverse reaction)
**Serious Adverse Events**	Event which requires hospitalization and/or causes mortality	Serious Adverse Event Form	Site Director or Clinical Research Coordinator	24 hours for hospitalization<br><br>24 hours for fatalities	**NIDCR via Rho Product Safety** (Simultaneously with IRB)<br><br>FDA (if associated with IP within 7 days after discovery)
**Unanticipated Problems**	Any problem or event which in the opinion of the local investigator was unanticipated, reflects new or increased risk to the subjects and was possibly related to the research procedures.	Unanticipated Problem Form	Site Director or Clinical Research Coordinator	5 business days from the date of discovery	**NIDCR via Rho Product Safety** (Simultaneously with IRB)<br><br>**FDA** (within 15 days if findings from other clinical, animal, or in-vitro studies that suggest significant human risk)

RUTGERS UNIVERSITY
Office for Research
APPROVED
IRB ID: Pro2020002299
APPROVAL: 1/8/2025
EXPIRATION: 1/7/2026

**Protocol Deviations**	Any change, divergence, or departure from the study design or procedures of a research protocol that is under the investigator's control and that has not been approved by the IRB.	Protocol Deviation/ Violation Form	Site Director or Clinical Research Coordinator	5 business days from the date of discovery	**Other study sites**<br><br>**Summary to DSMB in routine report** ***NIDCR_Reports @rhoworld.com***
**Protocol Violation**	Any deviation from the IRB approved protocol that may affect the subject's rights, safety, or well-being and/or the completeness, accuracy and reliability of the study data	Protocol Deviation/ Violation Form	Site Director or Clinical Research Coordinator	5 business days from the date of discovery	**Other study sites**<br><br>**Summary to DSMB in routine report** ***NIDCR_Reports @rhoworld.com***

All serious adverse events (SAEs) and all events determined to be UPs will be promptly reported to NIDCR, for assessment by the NIDCR Medical Monitor. Determining whether a particular adverse event is unexpected by virtue of an unexpectedly higher frequency can only be done through an analysis of appropriate data on all subjects enrolled in the research. If the site directors, PI and CCP Core Chief determine that an adverse event is not an unanticipated problem, but the NIDCR Medical Monitor subsequently determines that the adverse event does represent an unanticipated problem (for example, due to an unexpectedly higher frequency of the event), the NIDCR Medical Monitor will report this determination to the PI, and such reports must be promptly submitted by the investigator to the IRB.

SAEs, Unanticipated Problems (UPs) and study-related AEs will be collected and entered into the study REDCap system for reporting purposes. AEs that are related to the 3rd molar extraction surgical procedures and are not relevant to study outcomes will not be reported. AE relatedness will be assessed in a collaborative manner between the study PI, CCP chief and site director. While this trial will report study-related AEs, those AEs that are possibly, probably, or definitely study-related will be recorded in source documents. This will allow the study PI, CCPC chief and site directors to assess relatedness, expectedness, and severity.

Expected related adverse events are described in Section 9.3.2 below.

9.1.1 Unanticipated Problems

The Office for Human Research Protections (OHRP) considers unanticipated problems involving risks to subjects or others to include, in general, any incident, experience, or outcome that meets **all** of the following criteria:

- unexpected in terms of nature, severity, or frequency given (a) the research procedures that are described in the protocol-related documents, such as the IRB-approved research protocol and informed consent document; and (b) the characteristics of the subject population being studied;
- related or possibly related to participation in the research (“possibly related” means there is a reasonable possibility that the incident, experience, or outcome may have been caused by the procedures involved in the research); and
- suggests that the research places subjects or others at a greater risk of harm (including physical, psychological, economic, or social harm) than was previously known or recognized.

RUTGERS UNIVERSITY
Office for Research
APPROVED
IRB ID: Pro2020002299
APPROVAL: 1/8/2025
EXPIRATION: 1/7/2026

9.1.2 Adverse Events

An adverse event is any untoward or unfavorable medical occurrence in a human subject, including any abnormal sign (for example, abnormal physical exam or laboratory finding), symptom, or disease, temporally associated with the subject's participation in the research, whether or not considered related to the subject's participation in the research.

Hydrocodone, ibuprofen and acetaminophen all are known to cause adverse events. During study participation, subjects will be asked to record any adverse events on a daily basis through their eDiary entries. If the AE(s) become too bothersome or is severe, the subject will be instructed to contact the on-call surgeon who may advise the subject to stop taking their assigned analgesic. In addition, if the subject still requires analgesics, the on-call surgeon will determine whether the subject should be provided a rescue medication.

9.1.3 Serious Adverse Events

A serious adverse event (SAE) is one that meets one or more of the following criteria:

- Results in death
- Is life-threatening (places the subject at immediate risk of death from the event as it occurred)
- Results in inpatient hospitalization or prolongation of existing hospitalization
- Results in a persistent or significant disability or incapacity
- Results in a congenital anomaly or birth defect
- An important medical event that may not result in death, be life threatening, or require hospitalization may be considered an SAE when, based upon appropriate medical judgment, the event may jeopardize the subject and may require medical or surgical intervention to prevent one of the outcomes listed in this definition.

If a SAE becomes known, the Site Research Coordinator will immediately notify the study PI and Site Director (site PI). The Site Research Coordinator will record the event in the REDCap Serious Adverse Event Form based upon information obtained from the subject or surgeon-on call.

9.2 Time Period and Frequency for Event Assessment and Follow-Up

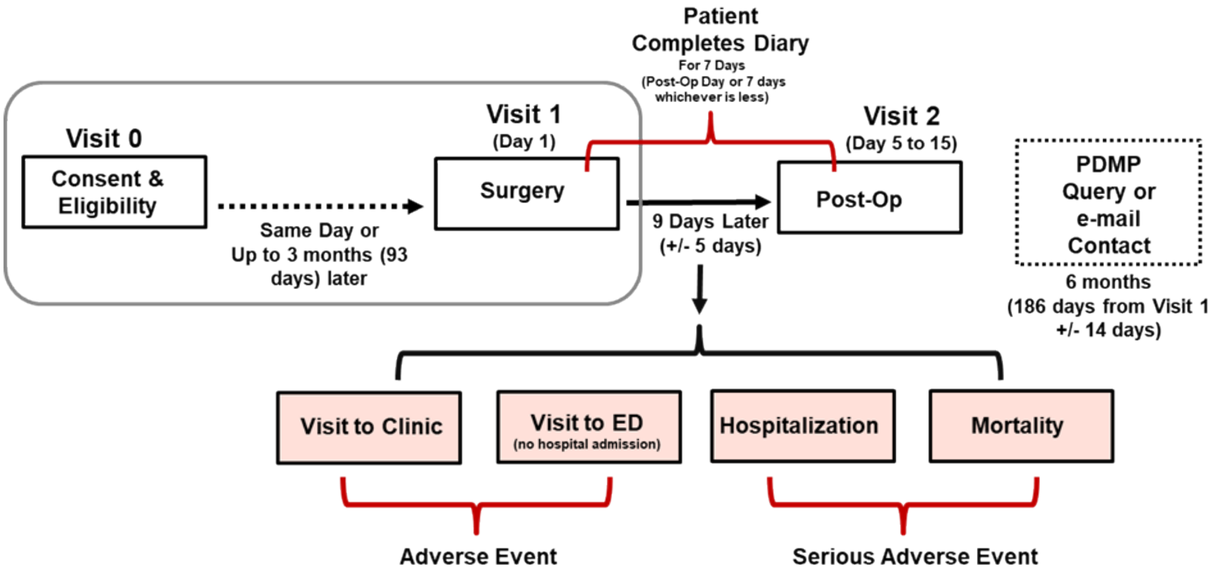

Documentation

RUTGERS UNIVERSITY
Office for Research
APPROVED
IRB ID: Pro2020002299
APPROVAL: 1/8/2025
EXPIRATION: 1/7/2026

In the event an after-hours emergency call was received, the Site Research Coordinator will request that a site surgeon contact the patient. During this follow-up call or if the subject calls the clinical site during normal business hours to report an adverse event, the severity of event will be assessed by the surgeon:

- If the symptom(s) are expected and no follow-up care is required, the subject will be counseled and directed by the surgeon to record the adverse event in their eDiary entry.
- If clinical follow-up care is required, the patient will be instructed by the surgeon to present to the dental clinic for evaluation.

In the event a subject visits either the dental clinic or the ED (without subsequent admission) for an adverse event, details will be documented on the Adverse Event Form by the Site Clinical Research Coordinator after consultation with the surgeon. The surgeon will determine whether the adverse event is related to the study intervention and this will be noted on the Adverse Event Form using the following criteria: Definite, Probable, Possible, Unlikely, and Not Related .

In the event a subject is hospitalized (with admission) and or experiences death during the study period, the event will be considered a SAE and details will be documented on the Serios Adverse Event Form by the Site Clinical Research Coordinator after consultation with the surgeon. All Serious Adverse Events forms will be reviewed by the PI and reported to the IRB, NIDCR through rho and the FDA within the required time frames.

The site PI will record all events with start dates occurring any time after surgery is obtained until 7 (for non-serious study-related AEs) or 30 days (for SAEs) after Visit 2. At each study visit, the investigator will inquire about the occurrence of AE/SAEs since the last visit. Events will be followed for outcome information until resolution or stabilization.

9.3 Characteristics of an Adverse Event

Each event will be recorded on an appropriate case report form that includes assessment of the characteristics defined below. These characteristics, along with the frequency of an event's occurrence, will be considered in determining if the event is a UP.

9.3.1 Relationship to Study Intervention

To assess relationship of an event to study intervention the following guidelines are used:

1. Related (Possible, Probable, Definite)
   a. The event is known to occur with the study intervention, and/or
   b. There is a temporal relationship between the intervention and event onset and/or
   c. The event abates when the intervention is discontinued, and/or
   d. The event reappears upon a re-challenge with the intervention.
2. Not Related (Unlikely, Not Related)
   a. There is no temporal relationship between the intervention and event onset, and/or
   b. An alternate etiology has been established.

RELATIONSHIP	ATTRIBUTION	DESCRIPTION
Unrelated to investigational agent/intervention[1]	Unrelated	The AE *is clearly **NOT** related* to the intervention
	Unlikely	The AE *is* ***doubtfully related*** to the intervention
Related to investigational agent/intervention[1]	Possible	The AE ***may be related*** to the intervention
	Probable	The AE ***is likely related*** to the intervention
	Definite	The AE ***is clearly related*** to the intervention

RUTGERS UNIVERSITY Office for Research
APPROVED
IRB ID: Pro2020002299
APPROVAL: 1/8/2025
EXPIRATION: 1/7/2026

9.3.2 *Expectedness*

The Study PI and/or study-appointed, clinically/medically responsible individual will determine whether an AE is expected or unexpected. The following are considered expected adverse events:

a. Pain
b. Bleeding
c. Excessive fatigue or drowsiness
d. Inability to concentrate
e. Dizziness
f. Euphoria (intense feeling of well-being & happiness)
g. Headache
h. Nausea
i. Vomiting
j. Diarrhea
k. Constipation
l. stomach aches
m. heartburn
n. itching
o. skin rashes
p. urinary retention
q. unintentional weight gain
r. other

Other Adverse Events will be considered unexpected if the nature, severity, or frequency of the event is not consistent with the risk information previously described for the intervention.

9.3.3 *Severity of Event*

The severity of the adverse events will be graded by the extent to which the subject is bothered by the event. Subject will determine whether they were bothered by the event using a scale: mild, moderate, or severe scale.:

- Mild- no impact or minimal impact on activities of daily living and no intervention required
- Moderate- moderate impact on activities of daily living which may require the subject seeks medical attention; affects instrumental ADLs
- Severe- significant impact on activities of daily living which require the subject to stop taking the study analgesic or subject seeks medical attention; affects self-care ADLs

Serious adverse events will be considered severe in extent as the symptoms are significant requiring hospitalization and invasive intervention.

9.4 Reporting Procedures

9.4.1 *Unanticipated Problem (UP) Reporting*

Incidents or events that meet the Office of Human Research Protection (OHRP) criteria for UPs require the creation and completion of an *Unanticipated Problem* form.

OHRP recommends that investigators include the following information when reporting an adverse event, or any other incident, experience, or outcome as an unanticipated problem to the IRB:

- appropriate identifying information for the research protocol, such as the title, investigator’s name, and the IRB project number;
- a detailed description of the adverse event, incident, experience, or outcome;

RUTGERS UNIVERSITY
Office for Research
APPROVED
IRB ID: Pro2020002299
APPROVAL: 1/8/2025
EXPIRATION: 1/7/2026

- an explanation of the basis for determining that the adverse event, incident, experience, or outcome represents an unanticipated problem;
- a description of any changes to the protocol or other corrective actions that have been taken or are proposed in response to the unanticipated problem.

To satisfy the requirement for prompt reporting, UPs that are not SAEs will be reported using the following timeline:

- Unanticipated problems will be reported to the IRB as soon as possible, and no later than within 5 business days of the investigator becoming aware of the event.

All unanticipated problems will be reported to appropriate institutional officials (as required by an institution's written reporting procedures), the supporting agency head (or designee), and OHRP within one month of the IRB's receipt of the report of the problem from the investigator.

Concurrently with reporting to the IRB, all unanticipated problems will be reported to the NIDCR via Rho Product Safety:

- Product Safety Email: rho_productsafety@rhoworld.com
- Product Safety Fax Line (US): 1-888-746-3293
- Product Safety Fax Line (International): 919-287-3998

9.4.2 Serious Adverse Event Reporting

Any AE meeting the specified Serious Adverse Event criteria, whether related or unrelated, will be submitted on an SAE form to NIDCR's centralized safety system via Rho Product Safety. This report may be sent by email or fax. Once submitted, Rho Product Safety will send a confirmation email to the investigator within 1 business day. The investigator should contact Rho Product Safety if this confirmation is not received. This process applies to both initial and follow-up SAE reports.

SAE Reporting Contact Information:

- Product Safety Email: rho_productsafety@rhoworld.com
- Product Safety Fax Line (US): 1-888-746-3293
- Product Safety Fax Line (International): 919-287-3998

General questions about UP or SAE reporting can be directed to the Rho Product Safety Help Line (available 8:00AM – 5:00PM Eastern Time):

- US: 1-888-746-7231
- International: 919-595-6486

The study's clinically responsible individual will provide an initial report of the SAE to the IRB and NIDCR as soon as possible, but no later than within 24 hours in the event of a study related events and within 5 business for other SAEs of the study team becoming aware of the event. If all descriptive information regarding the event is not available at the time of reporting, a follow-up report will be submitted as soon as additional information is available.

All SAEs will be followed until resolution or stabilization.

9.4.3 Reporting of Safety Events to FDA

As sponsor of IND 148217, the PI is required to comply with mandatory reporting of safety events to the Food and Drug Administration (FDA).

RUTGERS UNIVERSITY
Office for Research
APPROVED
IRB ID: Pro2020002299
APPROVAL: 1/8/2025
EXPIRATION: 1/7/2026

As per FDA regulations (*https://www.fda.gov/drugs/investigational-new-drug-ind-application/ind-application-reporting-safety-reports*), IND application sponsors are required to notify FDA in a written safety report of:

- any adverse experience associated with the use of the drug that is both serious and unexpected or
- any findings from tests in laboratory animals that suggest a significant risk for human subjects including reports of mutagenicity, teratogenicity, and carcinogenicity.

Initial reporting: IND application sponsor must report any suspected adverse reaction or adverse reaction to study treatment that is both serious and unexpected.
Unexpected serious suspected adverse reactions and observations from animal studies suggesting significant risk to human subjects must be reported to FDA as soon as possible but no later than within 15 calendar days following the sponsor's initial receipt of the information.

Unexpected fatal or life-threatening suspected adverse reactions represent especially important safety information and must be reported to FDA as soon as possible but no later than 7 calendar days following the sponsor's initial receipt of the information.

Follow-up reporting: Any relevant additional information obtained by the sponsor that pertains to a previously submitted IND safety report must be submitted as a Follow-up IND Safety Report. Such report should be submitted without delay, as soon as the information is available but no later than 15 calendar days after the sponsor receives the information.

All IND safety reports must be submitted on Form 3500A.

As sponsor of IND 148217, the PI is responsible to comply with the FDCA (21 U.S.C. §§ 301 et. seq.) as well as the implementing regulations [Title 21 of the Code of Federal Regulations (CFR)]. Responsibilities include:

- Reporting any unexpected fatal or life-threatening suspected adverse reactions to this Division no later than 7 calendar days after initial receipt of the information[21 CFR 312.32(c)(2)].
- Reporting any (1) serious, unexpected suspected adverse reactions, (2) findings from other clinical, animal, or in-vitro studies that suggest significant human risk, and (3) a clinically important increase in the rate of a serious suspected adverse reaction to this Division and to all investigators no later than 15 calendar days after determining that the information qualifies for reporting [21 CFR 312.32(c)(1)].
- Submitting annual progress reports within 60 days of the anniversary of the date that the IND became active (the date clinical studies were permitted to begin) [21 CFR 312.33].

NIDCR will be copied on expedited safety reporting to FDA.

For details, see timetable for reporting specified in Section 9.1 of the protocol.

9.4.4 Reporting of Pregnancy

Should a participant become pregnant, the subject will immediately be instructed to discontinue any study analgesics. An Unanticipated Problem Form will be completed. The participant will remain in the study, and data collection procedures will occur.

9.5 Halting Rules

Should there be a fatality due to the study analgesic or should there be 2 hospital admissions for the same serious adverse event, the study will be halted for a safety review.

RUTGERS UNIVERSITY
Office for Research
APPROVED
IRB ID: Pro2020002299
APPROVAL: 1/8/2025
EXPIRATION: 1/7/2026

Other circumstances that may warrant termination include, but are not limited to:

- Determination of unexpected, significant, or unacceptable risk to subjects. on
- Insufficient adherence to protocol requirements.
- Data that are not sufficiently complete and/or evaluable.
- Determination of futility.

RUTGERS UNIVERSITY
Office for Research

APPROVED
IRB ID: Pro2020002299
APPROVAL: 1/8/2025
EXPIRATION: 1/7/2026

10 STUDY OVERSIGHT

In addition to the PI's responsibility for oversight, study oversight will be under the direction of a Data and Safety Monitoring Board (DSMB) composed of members with expertise in the appropriate pain control, addiction, clinical trial methodology, and statistical disciplines. The DSMB will meet at least once per year to assess safety and efficacy data (if applicable), study progress, and data integrity for the study. If safety concerns arise, more frequent meetings may be held. The DSMB will operate under the rules of an NIDCR-approved charter that will be approved at the organizational meeting of the DSMB. At this time, most data elements that the DSMB needs to assess will be clearly defined. The DSMB will provide recommendations to the NIDCR.

RUTGERS UNIVERSITY
Office for Research
APPROVED
IRB ID: Pro2020002299
APPROVAL: 1/8/2025
EXPIRATION: 1/7/2026

11 CLINICAL SITE MONITORING

Clinical site monitoring is conducted to ensure that the rights of human subjects are protected, that the study is implemented in accordance with the protocol and/or other operating procedures, and that the quality and integrity of study data and data collection methods are maintained. Monitoring for this study will be performed by NIDCR's CROMS contractor. The monitor will evaluate study processes and documentation based on the International Council for Harmonisation (ICH), E6: Good Clinical Practice guidelines (GCP).

Details of clinical site monitoring will be documented in a Clinical Monitoring Plan (CMP). The CMP will specify

- The frequency of monitoring
- Monitoring procedures
- The level of clinical site monitoring activities (e.g., the percentage of participant data to be reviewed)
- Distribution of monitoring reports,

Some monitoring activities may be performed remotely, while others will take place at the study site(s). Staff from CROMS will conduct monitoring activities and provide reports of the findings and associated action items in accordance with the details described in the CMP. Documentation of monitoring activities and findings will be provided to the site study team, the study PIs, NIDCR-OCTOM and NIDCR program staff.

RUTGERS UNIVERSITY
Office for Research
APPROVED
IRB ID: Pro2020002299
APPROVAL: 1/8/2025
EXPIRATION: 1/7/2026

12 STATISTICAL CONSIDERATIONS

12.1 Study Hypotheses

The objectives and study hypotheses are stated below:

Objective 1 - *Pain experience and Patient satisfaction:*

Hypothesis 1.1 *NON-OPIOID is at least as or more effective (non-inferior to) than OPIOID for pain management.*

Hypothesis 1.2 *NON-OPIOID patient satisfaction will be greater than OPIOID patient satisfaction.*

Objective 2 – *Adverse Effects, Daily Function and Sleep Quality, and Opioid Seeking Behavior*

Hypothesis 2.1 Participants receiving NON-OPIOID will experience fewer and less severe adverse events, experience superior sleep and daily function, and exhibit less opioid seeking behavior than participants receiving OPIOID.

Hypothesis 2.2 Participants receiving 5 days of opioid-containing analgesics will have tablets/capsules remaining after their acute pain episode has been resolved.

Objective 3 – Development of Clinical Protocol/Decision Support Tool. This is an exploratory objective that will be hypothesis-generating.

To address the possible heterogeneity in pain management and side events in men and women due to sex differences and differences in pain tolerance, we will perform subgroup analysis in men and women separately to ensure the hypothesized differences between the analgesic groups still remain.

12.2 Sample Size Considerations

The following address the sample size and power for the entire sample analysis and subgroup analysis.

Sample Size and Power: To address Objective 1 and test if the non-opioid analgesics are non-inferior to opioid analgesics for pain management (**Hypothesis 1.1**), we estimated the sample size based on data of Chang et al.[20] where NRS was used to measure pain intensity. Based on non-inferiority margin d=1.0, and assuming that the maximum standard deviation (SD) of the daily pain intensity in our study is 3.6 (similar to the SD=3.6 for pain intensity reduction in patients receiving hydrocodone 5mg/acetaminophen 325mg in Chang et al.), our study needs at least **370** participants/analgesic group to test non-inferiority of non-opioid analgesics with 90% power and alpha=0.625% (one-sided, after Bonferroni correction for 4 tests of pain experience) [76] (See Sample Size Calculation Table). To account for 15-20% loss of follow-up and missing data, and other factors not included in the sample size estimation, we will assign IP to **1800 participants** with **450 participants** in each group (2 analgesic groups x 2 gender subgroups (men and women) to have >90% power to test non-inferiority of non-opioid analgesics in the entire sample. **Power for subgroup analysis:** With n=370/group (after attrition/missing data, etc.), non-inferiority margin d=1.0 and alpha=0.3125% (one-sided), we have 85% power to test non-inferiority of non-opioid analgesics in men and women separately.

RUTGERS UNIVERSITY
Office for Research
APPROVED
IRB ID: Pro2020002299
APPROVAL: 1/8/2025
EXPIRATION: 1/7/2026

To compare patient satisfaction (**Hypothesis 1.2**), we estimated the minimal detectable difference in proportions of positive rating between NON-OPIOID vs. OPIOID for n=740/group (entire sample) and n=370/group (subgroup) analyses. Based on data in Daniels et al.,(21) the proportion of positive rating (good, very good, excellent) for NON-OPIOID (1 ibuprofen 400mg/paracetamol 500mg) was 81.6%.(21) Assuming the proportion of positive rating (extremely satisfied and satisfied) for NON-OPIOID in our study (p1) is similar, say 82%, then our study has 90% power to test a minimal difference of 7% (82% vs. 75%) for n=740/group (entire sample, 2-sided alpha=5%) and 11% (82% vs. 71%) for n=370/group (subgroup analysis, 2-sided alpha=2.5%), in comparing proportion of positive rating of patient satisfaction. For continuous measures (e.g., mean normal-daily-function ratings and the sleep quality scores (**Aim 2**)), due to the lack of prior data in the literature that showed effect size in similar studies, we estimated the minimal detectable difference, in terms of Cohen's d, based on the sample size of n=740/group (entire sample) and n=370/group (subgroup analysis). Cohen described d~0.2 for small, ~0.5 for medium and ~0.8 for large effect size. Our study has 90% power to test a small effect size, Cohen's d of 0.20, for the entire sample (2-sided alpha=1.25%, after Bonferroni corrections for 4 tests) and Cohen's d=0.30 for the subgroup analysis (2-sided alpha=0.625%).

Consideration of within-site correlation: In the sample size and minimal effect size estimations, we tentatively assumed all individuals are independent. However, in our study, the randomization will be performed *within each site.* When the correlation between responses among participants within the same site is positive, randomizing participants within site can increase power: Consider a site of 2m participants and each patient is randomized to one of two treatments at a 1:1 ratio. Assume that the within-site correlation is r>0 and denote the standard deviation of each individual's response by σ. The variance of the mean difference in the responses between two treatments is $2\sigma^2/m*(1-r)$. It is smaller than $2\sigma^2/m$, the variance of the same difference when individuals are mutually independent. The smaller variance implies greater efficiency/power. Therefore, we expect our estimated sample size and minimal effect size to be more conservative than those in the actual trial. Therefore, the estimated sample size n=740/group for the entire sample and n=370/group for subgroup analysis would be sufficient.

12.3 Planned Interim Analyses (if applicable)

We will not perform interim analyses for Aims 1-3.

12.3.1 Safety Review

We will not perform interim analyses to investigate safety.

12.3.2 Efficacy Review

We will not perform interim analyses to investigate the efficacy of the outcomes.

12.4 Final Analysis Plan

\

The following Statistical Analysis Plan provides the statistical analysis plan for the full scope of the OARS study. However, this paper only included the data analyses that address Aim 1 and Aim 2.

Statistical Analysis Plan (Including Management of Missing Data)

All statistical analyses will be performed on an intent-to-treat (ITT) basis. For each Aim, summary statistics and histograms will be calculated and plotted to explore the differences in the distributions of data, cross-sectionally and longitudinally, between treatment conditions (NON-OPIOID vs. OPIOID).

RUTGERS UNIVERSITY
Office for Research
APPROVED
IRB ID: Pro2020002299
APPROVAL: 1/8/2025
EXPIRATION: 1/7/2026

Although we do not anticipate baseline differences in the distribution of measured factors between treatment conditions, we will assess the statistical differences using appropriate parametric and non-parametric tests. Variables that show a baseline difference ($p<0.1$) will be controlled as potential confounders in statistical models, when appropriate. Some variable transformation (e.g., logarithm) might be applied to meet regression model assumptions.

To account for the repeated measures design and the correlations within study site (due to participants sharing same facility, patient care management, etc.), generalized linear mixed model (GLMM) analysis,[77] including mixed model and random effects logistic regression analyses, will be used to analyze the primary and secondary outcomes. In these analyses, participants and sites will be treated as random effects, where appropriate.

For each test of an outcome, we define the statistical significance by $p<0.05$. Bonferroni correction will be applied for multiple testing, where appropriate.

12.4.1 Aim1 - *Pain experience and Patient satisfaction*

<u>Hypothesis 1.1</u> NON-OPIOID is at least as or more effective (non-inferior to) than OPIOID for pain management.

To test Hypothesis 1.1, mixed model analysis will be used to model the NRS pain ratings as a function of analgesic groups (non-opioid vs. opioid), day (e.g., day of treatment, day 2, day 3 upto day 8 post surgery) and day x analgesic group interactions as fixed effects. Measurements obtained from morning and evening e-diaries will be treated as clustered data within each day. Participants and sites will be adjusted using nested random effects to account for the possibly stronger within-patient than within-site correlations in the data. Baseline variables that show a statistical difference ($p<0.1$) will be controlled as covariates in the statistical model, where appropriate. Linear contrasts will be constructed to calculate and compare the mean differences in NRS pain ratings between NON-OPIOID and OPIOID ($\mu_{NonOpioid,t} - \mu_{Opioid,t}$) as the averages of first Day and Night (t=1), second Day and Night (t=2), third Day and Night (t=3), and the entire post-op period (t=4). To test the non-inferiority of the non-opioid analgesics H_0: ($\mu_{NonOpioid,t} - \mu_{Opioid,t}$)≥d vs. H_1: ($\mu_{NonOpioid,t} - \mu_{Opioid,t}$)<d, for t=1,2, 3, and 4, we chose a small and clinically nonsignificant difference d=1.0 as the non-inferiority margin.[78] NRS is a numerical rating scale (0-10 for no to worst possible pain) with a high correlation of 0.86-0.95 with VAS (0- 100mm for no to worst possible pain.)[79,80] Because there is limited literature suggesting the cutoff for clinically meaningful pain improvement, we chose the non-inferiority margin d of 1.0 in NRS, about 10mm in VAS, following the recommendation that a change in pain severity by VAS of 13mm (about equivalent to NRS of 1.3) as considered clinically meaningful.[80,81] The non-inferiority will be assessed using the one-sided 99.375% [82] or two-sided 98.75%, (after Bonferroni correction for 4 tests of pain experience) confidence interval (CI) of $\mu_{NonOpioid,t} - \mu_{Opioid,t}$, for t=1,2,3 and 4 defined above. If this CI completely lies –below d, we then conclude the non-inferiority of the non-opioid analgesics; if it completely lies below 0, we then conclude the (statistical) superiority of the non-opioid analgesics at the 1.25% level (two-sided).[82,83]

A quasi-per-protocol analysis, defined as a Pure IP analysis that only include patients who take at least one full dose of IP on the first Day and Night, second Day and Night, and no OTC medications in those 48 hours post-surgery, will be performed as a sensitivity analysis.

Summary statistics of numbers of pills that participants have taken during the entire period from the day of treatment until post-op will also be reported.

Percentage of participants taking a rescue medication will be compared between NON-OPIOID and OPIOID groups using the random effects logistic regression analysis. Participant need of rescue medication (yes/no during the entire study period) will be the dependent variable and the analgesic groups (NON-OPIOID vs.

RUTGERS UNIVERSITY
Office for Research
APPROVED
IRB ID: Pro2020002299
APPROVAL: 1/8/2025
EXPIRATION: 1/7/2026

OPOID) will be the independent variable as fixed effects in the statistical model. Site will be controlled as a random effect to account for the correlation between observations within a site.

Hypothesis 1.2 NON-OPIOID patient satisfaction will be greater than OPIOID patient satisfaction.

To test Hypothesis 1.2, we will calculate the proportion of each satisfaction category (e.g., very satisfied, satisfied, neither satisfied nor dissatisfied, dissatisfied and extremely dissatisfied) and apply the chi-square test to compare between NONOPIOID vs. OPIOID. Random effect logistic regression analysis will be used to compare the distribution of satisfaction (very satisfied and satisfied combined vs. neither satisfied nor dissatisfied, dissatisfied and very dissatisfied combined) between NON-OPIOID vs. OPIOID, controlling for covariates. Patient satisfaction is a one-time measurement (asked on the day of post-op visit). Only site will be adjusted as a random effect in the statistical model.

12.4.2 Aim 2 – *Adverse-Effects, Daily Function and Sleep Quality, and Opioid Seeking Behavior*

Hypothesis 2.1 Participants receiving NON-OPIOID will experience fewer and less severe adverse events, experience superior sleep and daily function, and exhibit less opioid seeking behavior than participants receiving OPIOID.

Hypothesis 2.2 Participants receiving 5 days of opioid-containing analgesics will have tablets/capsules remaining after their acute pain episode has been resolved.

Adverse Events. Percentage of participants who have at least one adverse event will be compared using the random effects logistic regression mode. with he analgesic group (NON-OPIOID vs. OPIOID) as the independent variable (fixed effects). Site will be adjusted as a random effect in the statistical models.

Daily Function. Mean normal-daily-function ratings will be compared between NON-OPIOID vs. OPIOID using the same method of mixed model analysis described in Hypothesis 1.1, with each of the normal-daily-function ratings and the sleep quality scores as the dependent variable.

Linear contrasts will be constructed to compare the changes/improvements in normal daily function and sleep quality in the conventional way (not as in testing non-inferiority) across the entire study period between NON-OPIOID vs. OPIOID.

Sleep Quality. The same method that compares daily function will be applied to sleep quality scores.

Future Opioid Seeking Behavior. We hypothesize that participants receiving opioid prescriptions to help manage acute pain are more likely to receive at least one additional opioid prescription within 6 months. This outcome will be measured through a PDMP check at the 6 month point. Summary statistics will be calculated to provide the number and percentage of participants filling opioid prescriptions within 6 months post-surgery for each group.

***Drug diversion*.** We anticipate that participants receiving 5 days of opioid containing analgesics will have capsules remaining after their acute pain episode has been resolved. Descriptive statistics will be calculated and percent of participants who have capsules remaining and the number of capsules remaining will be reported.

12.4.3 Aim 3 - Clinical Protocol/Decision Support Tool.

RUTGERS UNIVERSITY Office for Research
APPROVED
IRB ID: Pro2020002299
APPROVAL: 1/8/2025
EXPIRATION: 1/7/2026

To address Aim 3, we will develop an optimal[84-86] rule (model), as a function of patient characteristics, to recommend which analgesic to prescribe to maximize a patient's overall satisfaction.

We will first divide our sample into a training set and a testing set at 1:1 ratio. In the training set, we will use the method in section 2 of Xu et al [84] to develop the optimal rule[85,86](a statistical model) defined as $D^*(X) = \underset{D}{argmin} E\left[\frac{I(T \neq D(X))}{T\pi + (1-T)/2} Y\right]$, which can be turned into an optimization problem that finds $\beta^*(X) = \underset{\beta \in R^P}{argmin} E \left[\frac{\varphi(TX^T\beta)}{T\pi + \frac{1-T}{2}} Y\right]$ such that the optimal rule of treatment would be *$D^*(X)=sign(X^T\beta^*)$,* where D(X) denotes the treatment rule depending on covariates *X* (e.g., patient characteristics), T denotes the treatment assignment with T=1 for non-opioid and T= -1 for opioid medications, π denotes the proportion that patients receive non-opioid (i.e., π=P(T=1)), Y denotes patient satisfaction, and φ (.) is a convex and continuous loss function. Per Xu et al.[84], $\beta^*(X)$ can be determined by solving

$$\hat{\beta} = \underset{\beta \in R^P}{argmin} \left\{ \frac{1}{n} \sum_{i=1}^{n} \frac{\varphi(T_i X_i^T \beta)}{T_i \pi + (1 - T_i)/2} Y_i + \lambda_{1n} \|\beta\|_1 + \lambda_{2n} \eta(\beta) \right\},$$

where $\|\beta\|_1$ denotes the L_1 norm of β, $\eta(\beta)$ denotes the fuse Lasso penalty[87], with the tuning parameters λ_1 and λ_{2n} determined by the 10-fold cross-validation approach, and n denotes the sample size in the training set. To determine the optimal rule for the overall patient satisfaction, we will choose the logit loss function for φ (.) and X will be determined from patient characteristics such as patient's pain sensitivity, expectations, number and location (maxillary vs. mandibular extractions), level of surgical difficulty, surgical time, education level, gender, race and ethnicity, BMI and age, and possible interaction terms among these variables, and the empirical optimal treatment decision rule would be $\hat{D} = sign(X^T\hat{\beta})$.

In the testing set, we will use the method in Section 3 of Xu et al.[84], to evaluate the improvement of patient overall satisfaction, defined by $d = E[Y|sign(X^T\hat{\beta}) = T] - E[Y|sign(X^T\hat{\beta}) \neq T]$, and use the m-out-of-n bootstrap method[88,89], when assigning patients to appropriate treatments according to the optimal rule developed in the training set.

The methods of Xu et al.[84] to develop and evaluate the optimal rule will be implemented using the R code provided as *supplements* at *https://onlinelibrary.wiley.com/doi/epdf/10.1111/biom.12322*.

Heterogeneity and Subgroup Analyses
To address the possible heterogeneity in pain management and side events in men and women due to sex differences and differences in pain tolerance, we will perform subgroup analysis in men and women separately to ensure the hypothesized differences between the analgesic groups still remain. All the statistical analyses described above will be performed on the entire sample, and repeated for men and women respectively as subgroup analyses.

For Aim 3, we will develop the men- and women-specific rules (models) using the method of Xu et al.[84] If these rules (models) differ by the set of selected patient characteristics and/or their corresponding effect sizes (coefficient estimates in the model) between men and women, we will report the men- and women-specific rules to recommend analgesic prescriptions. Otherwise, we will report the optimal rule derived from the entire sample with gender as a candidate patient characteristic in the variable selection process.

Findings form Aim 1 and Aim 2 main and subgroup analyses will also be used to facilitate the model interpretation.

***Sensitivity analyses* - *Site effect*.** Sensitivity analysis will be performed to assess if the differences in NON-OPIOID and OPIOID varies by site. This will be assessed by either adding interaction terms with site (i.e., site x analgesic groups, site x day and site x day x analgesic groups) to the statistical models, or using

RUTGERS UNIVERSITY Office for Research
APPROVED
IRB ID: Pro2020002299
APPROVAL: 1/8/2025
EXPIRATION: 1/7/2026

stratified analysis (stratified by site) and applying the Wald test by constructing linear contrasts to compare site specific NON-OPIOID-vs-OPIOID differences across all sites.

Statistical Treatment for Missing Data. To assess the impact of missing data when we compare the outcomes (e.g., pain and patient satisfaction) between OPIOID and NON-OPIOID, we will use multiple imputation and last value carried forward analyses as sensitivity analyses to check whether the results of ITT analysis are robust. For the multiple imputation analysis, we will assume missing at random (MAR) and generate ten imputed datasets by the sequential regression approach[90] (also called Chained Equations or Flexible Conditional Specifications) using the software package IVEware. Data analysis will be performed on each imputed dataset with results combined using the Rubin's Rule via PROC MIAnalyze in SAS. The multiple imputation analyses will be applied to the outcomes of pain and patient satisfaction. Because patient satisfaction will be a one-time outcome measure obtained at the post-operative visit, only multiple imputation analysis will be applied.

RUTGERS UNIVERSITY
Office for Research
APPROVED
IRB ID: Pro2020002299
APPROVAL: 1/8/2025
EXPIRATION: 1/7/2026

13 SOURCE DOCUMENTS AND ACCESS TO SOURCE DATA/DOCUMENTS

All study information will be captured electronically and stored on secure, encrypted Rutgers University servers.

- Eligibility criteria will be recorded directly into REDCap via an electronic Case Report Form (eCRF) and documentation of consent will occur electronically within REDCap.
- Randomization sequence will be generated using REDCap or R software by the SDMC. Labels will be generated based upon the randomization sequence and provided to the Clinical Protocol Coordinating Core Research Coordinator, who will assemble study material kits and will ship to sites in randomized order.
- Pre-operative questionnaire, subject eDiary, and post-operative questionnaire will be captured directly into REDCap via eCRFs.
- Activity/sleep statistics will be captured electronically via Actigraph. Actigraph data will be downloaded into *SDM OARS:Data:Actigraph* (Box drive) with separate files maintained for each subject. Data will be electronically extracted when files are being merged for statistical analysis.
- Study analgesic capsule usage and capsules remaining will be captured electronically via Nomi and SMRxT. Data will be electronically downloaded and data elements stored into the REDCap EDC project.
- Surgical Case Report and Post-Operative Case Report information will be obtained by the Site Research Coordinator interviewing the surgeon and directly entering responses into REDCap via eCRFs.
- PDMP data will be obtained by the clinical site research coordinator via a PDMP Query and entered directly into REDCap via the PDMP data CRF.

Study staff will maintain appropriate research records for this study, in compliance with ICH E6, Section 4.9 and regulatory and institutional requirements for the protection of confidentiality of participants. Study staff will permit authorized representatives of NIDCR, IRB and regulatory agencies to examine (and when required by applicable law, to copy) research records for the purposes of quality assurance reviews, audits, and evaluation of the study safety, progress, and data validity.

RUTGERS UNIVERSITY
Office for Research
APPROVED
IRB ID: Pro2020002299
APPROVAL: 1/8/2025
EXPIRATION: 1/7/2026

14 QUALITY CONTROL AND QUALITY ASSURANCE

Quality Management (QM) measures will occur throughout this study to ensure adherence to protocol and collection of high quality data. QM activities include those measures done as part of daily standard operating procedures by staff at each site and by the data management controls built into the OARS REDCap system to ensure adherence to the protocol and collection of complete and accurate data. QM activities will include activities undertaken at defined intervals to check that QM activities are taking place and study-related documents are up-to-date.

Detailed procedures can be found in the Clinical Quality Management Plan.

14.1 Staff Training

Training of all staff will be conducted and recorded in training logs. Core Directors and Site Directors (PIs) are responsible for ensuring all of their staff have completed the required modules before being assigned any subject interfacing activities. Each staff member has been identified by role along with required training for each staff role. Records of completed training will be maintained in each study member's study personnel file kept on the SDM OARS box drive, Personnel Qualifications folder. Notification of completed training will be sent to the Project Manager who will maintain investigator training records in REDCap via the On Boarding Checklist and Annual Checklist forms in the REDCap OARS Personnel Project.

Training Modules	Who Will be Trained	Record Storage
**General Clinical Research Training** (Human Subjects, Good Clinical Practice, HIPAA)	• All Study Personnel	SDM OARS Personnel Folder
**General Overall** (Study Purpose, Goals and Protocol)	• All Study Personnel<br>• Advisory Committee<br>• Monitoring Entities	REDCap Personnel Project
**General IT System Training** (REDCap)	• Steering Committee<br>• All Core Personnel<br>• All Site Personnel	REDCap Personnel Project
**Site Clinical Procedures**	• All Site Personnel	REDCap Personnel Project
**Core Study Materials Management**	• All Clinical Core Personnel	REDCap Personnel Project
**Clinical Quality Management Plan**	• Steering Committee	REDCap Personnel Project

14.2 Daily Operating Procedures

Quality management measures have been built into Clinical Protocol Core and site procedures.

Real-Time Subject Record Review: The REDCap dashboard will be utilized to monitor consents, forms, and questionnaires through a red/yellow/green indicator system. Upon completing a REDCap form or questionnaire, the Site Research Coordinator will be mark the document as "unverified", indicated by a yellow light on the REDCap dashboard. Through the review process, the Site Data Quality Analyst will evaluate all applicable documents to ensure proper form usage and the accuracy and completeness in the all required fields. If a record is complete and accurate, the Site Data Quality Analyst will change the record to "Complete", as indicated by a green indicator on the REDCap dashboard. The Site Data Quality Analyst will be responsible to report deficiencies to appropriate authorities and follow-up reporting as more information is obtained If a form requires correction, the Site Research Coordinator will be asked, if possible, to correct the deficiency or communicate with the subject to rectify. Any deficiencies that cannot be corrected will be recorded for quality improvement, and deficiencies will be reviewed monthly amongst study staff for quality improvement.

Pre-programmed quality checks

RUTGERS UNIVERSITY Office for Research
APPROVED
IRB ID: Pro2020002299
APPROVAL: 1/8/2025
EXPIRATION: 1/7/2026

Structured responses are required for the CRFs so only valid responses can be recorded. In addition, REDCap has been programmed with all responses being required at time of completion. Validation rules are employed where possible.

To summarize, these checks will be performed on the following forms/surveys:

Visit	Forms	Comments
**Visit 0**	• Consent to Participate in a Research Study<br>• Eligibility Determination	In addition to review by the Site Data Quality Analyst, there are programmatic functions within REDCap system to serve as quality checks.
**Visit 1**	• Gender and Pregnancy Determination<br>• Pre-Op Questionnaire<br>• ID Link and SMS Test<br>• SMS Test and Training<br>• Surgical Case Report	
**Visit 2**	• Post-Op Case Report<br>• Post-Op Questionnaire<br>• Payment Acknowledgment	
**Intermediate Visit/Call**	• Adverse Event form	This review will be completed when there is an emergency call or intermediate or emergency visit to the OMFS clinic, hospital emergency department of hospital admission

Managing of Study Materials: Manual of Procedures, Section 5.1.4.2, describes the process to be followed when preparing a subject packet of materials by the CPC Core Research Coordinator, when receiving participant packets at each clinical site, when materials are issued to a subject and when study materials are returned by the subject. As part of this process, bar codes will be scanned to enable tracking of all materials.

14.3 Subject Completion Review

Within 14 days (or as soon as possible) after a subject completes Visit 2, a payment card is issued, or a subject termination is recorded in the Adverse Event form, the Site Data Quality Analyst will review the entire subject's research record to determine if all forms/questionnaires have been completed and signatures present. The REDCap Subject Completion Checklist will be used for this review, and any discrepancies will be noted via a Query using the REDCap Query system which requires an entry describing the issue with a request to the Site Research Coordinator or Site Director to correct the deficiency. A response by the Site Research Coordinator or Site Director is required, documenting that the deficiency has been corrected, or if it is not possible to correct the deficiency, documenting the reason why it can not be rectified.

Forms/Surveys to be reviewed for completeness and signatures include:

- Consent
- Eligibility Form
- Pregnancy
- Training/SMS
- Pre-Operative Questionnaire
- Surgical Case Report
- Post-Operative Questionnaire
- Intermediate Visit/Call
- Site Director Subject Close-Out

RUTGERS UNIVERSITY
Office for Research
APPROVED
IRB ID: Pro2020002299
APPROVAL: 1/8/2025
EXPIRATION: 1/7/2026

14.4 Data Management Controls

REDCap is a rich system which enables privileging, required data element entry and data validation to be embedded into daily operations. These activities, summarized below, have been detailed in the Data Management Plan (DMP).

System	Control
**REDCap**	**Privileging:** Every study staff member will be assigned a role which carries specific read/write/edit/delete privileges. Staff member will be assigned a role providing minimal rights sufficient to perform his/her responsibilities.<br><br>**Structured Data:** Whenever possible, data will be collected via structured data responses rather than free-text. Structured data requires a respondent to check a valid response.<br><br>**Data Validation:** Data fields will be established limiting responses to those that are reasonable. For example, year of birth would have a range of 2002 (corresponding to age 18 – the youngest eligible) to 2060 (corresponding to age 80 – more than sufficient to cover the age range of individuals having impacted 3rd molars extracted.)<br><br>**Required Fields:** Whenever appropriate, fields have been made required so as to ensure completed forms and questionnaires.<br><br>**Go Rules:** Surveys and forms have been developed in a way which will not a user to proceed to the next survey or form without a predecessor form being completed with all required fields.

14.5 Quality Management Review Summary

The quality management review activities are detailed in the Clinical Quality Management Plan (CQMP) and summarized below:

Component	Purpose	Frequency	Responsibility	Core or Site Level	Tool
Essential Document Review	• Ensure all essential documents are being maintained	Annual (January)	Chief – Clinical Protocol Core	Core	OARS Annual Essential Document Review Tool
MOP and CQMP Review	• Ensure MOP is still appropriate and CQMP is being followed	Annual (January)	Chief – Clinical Protocol Core	Core	OARS Annual MOP and CQMP Review Tool
Personnel and MOP Compliance Review	• Ensure all personnel have active credentials and have completed training<br>• Ensure compliance with MOP<br>• Study product accountability	Quarterly (January, April, July, October)	Chief – Statistics and Data Quality Core	Cores & Sites	OARS MOP Compliance Tool (REDCap Form)
Study Participant Record Review	• Review at least 10 records with Site Director sign-off at each site to determine completeness of participant records	Quarterly (January, April, July, October)	Chief – Statistics and Data Quality Core	Sites	OARS Participant Record Review Tool (REDCap Form)

RUTGERS UNIVERSITY
Office for Research
APPROVED
IRB ID: Pro2020002299
APPROVAL: 1/8/2025
EXPIRATION: 1/7/2026

15 ETHICS/PROTECTION OF HUMAN SUBJECTS

15.1 Ethical Standard

The investigator will ensure that this study is conducted in full conformity with the principles set forth in The Belmont Report: Ethical Principles and Guidelines for the Protection of Human Subjects of Research, as drafted by the US National Commission for the Protection of Human Subjects of Biomedical and Behavioral Research (April 18, 1979) and codified in 45 CFR Part 46 and/or the ICH E6.

15.2 Institutional Review Board

The protocol, informed consent form, recruitment materials, and all participant materials will be submitted to the sIRB for review and approval and will be submitted to the ceding IRBs as requested for local context review. Approval of both the protocol and the consent form(s) must be obtained before any participant is enrolled. Any amendment to the protocol will require review and approval by the sIRB before the changes are implemented in the study.

15.3 Informed Consent Process

Informed consent is a process that is initiated prior to the individual agreeing to participate in the study and continues throughout study participation. Extensive discussion of risks and possible benefits of study participation will be provided to participants and their families, if applicable. A consent form describing in detail the study procedures and risks will be given to the participant. Consent forms will be IRB-approved, and the participant is required to read and review the document or have the document read to him or her. The investigator or designee will explain the research study to the participant and answer any questions that may arise. The participant will sign the informed consent document prior to any study-related assessments or procedures. The participant may withdraw consent at any time throughout the course of the study. A copy of the executed consent form (either paper or electronic) will be provided to the patient at the time of consent for their records. An electronic version of the signed informed consent documents be maintained in REDCap and an additional copy can be provided to participants at any time upon request.. The rights and welfare of the participants will be protected by emphasizing to them that the quality of their clinical care will not be adversely affected if they decline to participate in this study.

The consent process will be documented in the research record.

As this is a multi-site study, each participating institution will be provided with a model informed consent form. Each institution may revise or add information to comply with institution consent templates, but may not remove procedural or risk content from the model consent form.

15.4 Exclusion of Women, Minorities, and Children (Special Populations)

Women and minorities will be eligible to participate. Children less than 18 years will not be able participate.

15.5 Subject Confidentiality

Subject confidentiality is strictly held in trust by the investigators, study staff, and the study sponsor(s) and their agents.

The study protocol, documentation, data, and all other information generated will be held in strict confidence. No information concerning the study or the data will be released to any unauthorized third party without prior written approval of the study sponsor.

The study monitor or other authorized representatives of NIDCR, IRB representatives and other regulatory representatives, may inspect all study documents and records required to be maintained by the investigator,

RUTGERS UNIVERSITY Office for Research
APPROVED
IRB ID: Pro2020002299
APPROVAL: 1/8/2025
EXPIRATION: 1/7/2026

including but not limited to, study records for the study participants. The clinical study site will permit access to such records.

<u>Certificate of Confidentiality</u>
To further protect the privacy of study participants, the Secretary, Health and Human Services (HHS), has issued a Certificate of Confidentiality (CoC) to all researchers engaged in biomedical, behavioral, clinical, or other human subjects research funded wholly or in part by the federal government. Recipients of NIH funding for human subjects research are required to protect identifiable research information from forced disclosure per the terms of the NIH Policy (*https://humansubjects.nih.gov/coc/index*). As set forth in 45 CFR Part 75.303(a) and NIHGPS Chapter 8.3, recipients conducting NIH-supported research covered by this Policy are required to establish and maintain effective internal controls (e.g., policies and procedures) that provide reasonable assurance that the award is managed in compliance with Federal statutes, regulations, and the terms and conditions of award. It is the NIH policy that investigators and others who have access to research records will not disclose identifying information except when the participant consents or in certain instances when federal, state, or local law or regulation requires disclosure. NIH expects investigators to inform research participants of the protections and the limits to protections provided by a Certificate issued by this Policy.

<u>NIH Data Sharing Policies</u>
As described in section 17, it is NIH policy that the results and accomplishments of the activities that it funds should be made available to the public (see *https://grants.nih.gov/policy/sharing.htm*). PIs and funding recipient institutions will ensure that all mechanisms used to share data include proper plans and safeguards to protect the rights and privacy of individuals who participate in NIH-sponsored research.

15.6 Future Use of Stored Specimens and Other Identifiable Data

No specimens will be collected as part of this study. All Identifiable data will be destroyed 7 years after the study is completed. Genetic testing will not be performed.

RUTGERS UNIVERSITY
Office for Research
APPROVED
IRB ID: Pro2020002299
APPROVAL: 1/8/2025
EXPIRATION: 1/7/2026

16 DATA HANDLING AND RECORD KEEPING

The investigators are responsible for ensuring the accuracy, completeness, legibility, and timeliness of the data reported. All source documents should be completed in a neat, legible manner to ensure accurate interpretation of data. The investigators will maintain adequate case histories of study participants, including accurate case report forms (CRFs), and source documentation.

16.1 Data Management Responsibilities

Data collection and accurate documentation are the responsibility of the study staff under the supervision of the site directors and study PI. The SDMC is responsible for overall review of the data to ensure quality and regular reporting of data to ensure completeness and accuracy of data collected.

16.2 Data Capture Methods

All data for the study be electronically captured. Various devices will be used including:
- Laptop and desktop computers to complete surveys and forms directly into REDCap, the electronic data capture system for the study
- iPads to complete surveys, forms and capture electronic signatures directly into REDCap
- Bar code scanner to capture study product information into REDCap
- SMRxT bottle to capture the date/time when a dosage of study product is removed from the bottle
- Acitgraph to capture sleep and daily activity data
- Participant smart phones for eDiary entries directly into REDCap

All study data will be centrally stored on the Rutgers REDCap data management system, and study-related documentation will be maintained on Rutgers Box servers. REDCap is a secure web application which is password protected and compliant with 21 CFR Part 11. Study data and documentation will be available to the study PI and core personnel in real time, per allowable permissions. Specific authentication, security, firewall, virus protection, backup and disaster recovery details can be found in the Data Management Plan (DMP).

16.3 Schedule and Content of Reports

The following reports/dashboards will be developed:

Report/ Dashboard	Frequency	Purpose	Content	Reviewed by
Enrollment Statistics	Monthly	To track enrollment by site	• # eligibility screenings<br>• # subjects enrolled<br>• # subjects randomized	• Site Directors<br>• Principal Investigator<br>• Site Research Coordinators<br>• NIDCR
Subject Completion Statistics	Monthly	To check quality monitoring by site	• # subjects completed	• Site Directors<br>• Principal Investigator<br>• Site Research Coordinators<br>• NIDCR (As part of enrollment/retention reports)
Missing Data/Signature Statistics	Monthly	To closely monitor missing information and provide an opportunity for quality improvement	• Frequency of missing data elements and signatures	• Site Directors<br>• Principal Investigator<br>• Site Research Coordinators
Protocol	Monthly	To closely monitor	• Listing of protocol	• Site Directors

RUTGERS UNIVERSITY
Office for Research
APPROVED
IRB ID: Pro2020002299
APPROVAL: 1/8/2025
EXPIRATION: 1/7/2026

Deviation Report		protocol deviations and provide an opportunity for quality improvement	deviations<br>• Frequency of types of protocol deviations	• Principal Investigator<br>• Site Research Coordinators<br>• DSMB (at designated meeting frequency)
Serious Adverse Reactions Report	Monthly	To closely track SAE’s	• Listing of SAE’s<br>• Frequency of types of SAE’s	• Site Directors<br>• Principal Investigator<br>• Site Research Coordinators<br>• sIRB<br>• DSMB (at designated meeting frequency)<br>•
Unanticipated Problem Report	Monthly	To closely track unanticipated problems and provide an opportunity for quality improvement	• Listing of unanticipated problem reports<br>• Frequency of types of UP’s	• Site Directors<br>• Principal Investigator<br>• Site Research Coordinators<br>• sIRB<br>• NIDCR via Rho Product Safety (concurrent with IRB reporting)<br>• DSMB (at designated meeting frequency)
MOP Compliance Review	Quarterly	To ensure compliance with Manual of Procedures	• Documents MOP Compliance Review and details findings and corrective actions	• Steering Committee<br>• Monitor<br>• NIDCR
Completed Subject Review	Quarterly	To ensure all data and signatures are present	• Documents Completed Subject Review and details findings and corrective actions	• Steering Committee<br>• Monitor<br>• DSMB- summary in QM section of report (at designated meeting frequency)
Essential Document Review Summary	Annual	To review essential documents enabling the updating of essential documents if necessary	• Documents Essential Document Review and details findings and corrective actions	• Steering Committee<br>• Monitor<br>•
MOP and CQMP Review	Annual	To review MOP and CQMP plan enabling refinements to these plans if necessary	• Documents MOP and CQMP review and details findings and corrective actions	• Steering Committee<br>• Monitor<br>• NIDCR

There will be no interim analysis before the database is locked. After the database is locked, group assignment will be unmasked for data analysis. Data analysis is detailed in Section 12.4.

16.4 Study Records Retention

Per Rutgers Research Regulatory Affairs, research that involves collection of protected health information (PHI) is subject to the HIPAA regulations. Research records including signed consent forms that contain the HIPAA authorization must be retained for 6 years after the date on which the subject signed the consent form or the date when it last was in effect, whichever is later.

RUTGERS UNIVERSITY
Office for Research
APPROVED
IRB ID: Pro2020002299
APPROVAL: 1/8/2025
EXPIRATION: 1/7/2026

16.5 Protocol Deviations

A protocol deviation is any change, divergence, or departure from the study design or procedures of a research protocol that is under the investigator's control and that has not been approved by the IRB. Upon discovery, the Principal Investigator is responsible for reporting protocol deviations to the IRB.

A protocol violation is a deviation from the IRB approved protocol that may affect the subject's rights, safety, or well-being and/or the completeness, accuracy and reliability of the study data. Protocol Violations must be submitted for Full Board IRB review. If the deviation meets any of the following criteria, it is considered a protocol violation.

Protocol deviations and protocol violations can be broken down into the following two categories: Minor deviation or violation OR Major deviation or violation.

- Minor Deviations and/or Minor Violations: A minor deviation or minor violation is viewed by the IRB as an event that does not impact subject safety, compromise the integrity of study data and/or affect a subject's willingness to participate in the study. Minor deviations or violations will be reviewed under expedited procedures by a single reviewer. The reviewer will determine whether the event is accepted as a minor deviation/violation and can recommend a corrective course of action. The deviation will be sent to the fully convened IRB meeting where the board will discuss and determine if any additional actions are required, where applicable and as necessary. If the event meets any of the following criteria, it is considered a minor deviation or minor violation: Examples (Minor)
- Major Deviations and/or Major Violations: A major deviation or violation is viewed by the IRB as an event that may impact subject safety, affect the integrity of study data and/or affect a subject's willingness to participate in the study. Major protocol deviations/violations are treated as noncompliance. These reports will be reviewed at the fully convened IRB meeting where the board will discuss the major deviations and/or major violations and determine of the appropriate course of action. If the event meets any of the following criteria, it is considered a major deviation or major violation: Examples (Major)

All deviations and violations from the protocol will be recorded on the Protocol Deviation/Violation Reporting Form no later than 5 business days after study staff become aware of the deviation and forwarded to the study PI for review and reported promptly to the IRB as necessary.

There are circumstances, which may arise, that are beyond the investigator's control and will not be reported to the IRB and other regulatory bodies as protocol deviations:

- Technology Failures
  - Actigraph not working properly
  - SMRxT bottle not working properly
  - Access to REDCap interrupted
- Patient Related
  - Surgical plan change before or during surgery

In addition, there are some events, which may arise, that necessitate action. These, too, will not be reported to the IRB and other regulatory bodies as protocol deviations since the protocol clearly outlines measures to be taken:

  - Need for rescue medication (Sections 6.3, 6.6)
  - Subjects returning for post-op outside of window
    - If the Post-Op exam is outside the window, the post-operative exam will still be conducted. However, the post-operative data will not be included in the data set
  - Subjects exceeding maximum daily dose

RUTGERS UNIVERSITY
Office for Research
APPROVED
IRB ID: Pro2020002299
APPROVAL: 1/8/2025
EXPIRATION: 1/7/2026

    - Patients will be contacted and re-educated in terms of dosing and use of OTC alternatives
    - An Adverse Event form will be competed if the patient presents to the clinic or to the ED for an intermediate visit
    - A Serious Adverse Event Form will be completed if the subject is hospitalized overnight or there is a fatality
  - Use of Exparel or other long lasting local anesthetic

All protocol deviations and violations will be reviewed monthly during the OARS Steering Committee Meetings.

RUTGERS UNIVERSITY
Office for Research
APPROVED
IRB ID: Pro2020002299
APPROVAL: 1/8/2025
EXPIRATION: 1/7/2026

17 PUBLICATION/DATA SHARING

This study will comply with all applicable NIH Data Sharing Policies. See *https://grants.nih.gov/policy/sharing.htm* for policies and resources.

Dissemination at Scientific Meetings: Presentations at scientific meetings will be delivered to assist in dissemination of results as soon as possible when final results pertaining to the primary variables are available. Meetings at which presentations will be made include, but will not be limited to, American and International Association of Dental Research (AADR and IADR), the American Dental Association (ADA), and International Association for the Study of Pain (IASP) and addiction meetings. NIH grant support will be acknowledged during all presentations.

Publication and Authorship Policies:
Findings will be published in peer-reviewed journals. Journals selected will be indexed in PubMed. Accepted manuscripts will be submitted to PubMed Central as per NIH policy. Examples of journals in which final results may be published include the Journal of Dental Research and the Journal of the American Dental Association. Other medical journals, including journals on pain and addiction will be considered for manuscript publications. Final versions of the peer reviewed manuscripts will be made available to the public, generally within 3 months but no later than 12 months after the official date of publication. NIH grant support will be acknowledged in all publications.

Proposals from study investigators for writing and submitting abstracts and manuscripts for publication will be presented to a committee for approval, comprised of the study PI, chief clinical officer, chief pharmacology officer and chief statistician. Included in the proposal is data needed for the publication, analyses to be performed, proposed authorship and order of authorship, and/or journal in which the manuscript will be published. If a submission for an approved proposal is not completed within 11 months of approval, other authors can submit a similar proposal to the committee for approval.

The following ICMJE guidelines will be adopted and followed in determining authorship:

- Substantial contributions to the conception or design of the work; or the acquisition, analysis, or interpretation of data for the work; AND
- Drafting the work or revising it critically for important intellectual content; AND
- Final approval of the version to be published; AND
- Agreement to be accountable for all aspects of the work in ensuring that questions related to the accuracy or integrity of any part of the work are appropriately investigated and resolved.

NIH Public Access Policy
The NIH *Public Access Policy* requires scientists to submit final peer-reviewed journal manuscripts that arise from NIH funds to *PubMed Central* immediately upon acceptance for publication. This ensures that the public has access to the published results of NIH funded research.

NIH Policy on the Dissemination of NIH-Funded Clinical Trial Information
The study is a clinical trial and will comply with the NIH policy that establishes the expectation that all investigators conducting clinical trials funded in whole or in part by the NIH will ensure that these trials are registered at ClinicalTrials.gov, and that results of these trials are submitted to ClinicalTrials.gov.

This clinical trial, when funded, will be registered on the ClincialTrials.gov by Cecile A. Feldman, DMD, the study principal investigator, no later than 21 days after enrollment of the first subject. Data to be posted at time of initial registration include descriptive information, recruitment information, location and contact information and administrative information. Final results will be posted no later than 12 months after the trial's primary completion date and will include participant flow, demographic and baseline characteristics,

RUTGERS UNIVERSITY
Office for Research
APPROVED
IRB ID: Pro2020002299
APPROVAL: 1/8/2025
EXPIRATION: 1/7/2026

outcomes and statistical analyses, adverse events, the protocol, statistical analysis plan and administrative information.

The PI will share the individual non-identified subject data no later than acceptance for the publication's main findings from the final data analysis or 18 months after completion of the study, whichever is earlier. As NIDCR does not have a data repository at the initiation of this study, data will be posted on the Rutgers designated clinical trials data warehouse. (If NIDCR has a clinical trial data repository at the time of study, completion, data will be posted on the NIDCR data repository.) Data to be posted will include the study protocol, reference to study publication of primary outcome variable, the data set in both SAS and ASCII formats, data dictionary and study specific de-identification notes.

Upon written request to the PI, survey instruments or other materials developed for use during the clinical trial will be made electronically available to other researchers.

<u>Food and Drug Administration Amendments Act of 2007 (FDAAA) and the Final Rule for Clinical Trials Registration and Results Information Submission</u>

This study is an applicable clinical trial and will comply with *U.S. Public Law 110-85* (Food and Drug Administration Amendments Act of 2007 or FDAAA), Title VIII, Section 801 and *42 CFR Part 11* (HHS Final Rule for Clinical Trials Registration and Results Information Submission), which mandate that a "responsible party" (i.e., the sponsor or designated principal investigator) register and report results of "applicable clinical trials."

RUTGERS UNIVERSITY
Office for Research
APPROVED
IRB ID: Pro2020002299
APPROVAL: 1/8/2025
EXPIRATION: 1/7/2026

18 LITERATURE REFERENCES

1. Baker JA, Avorn J, Levin R and Bateman BT. Opioid Prescribing After Surgical Extraction of Teeth in Medicaid Patients, 2000-2010. JAMA. 2016;315(15):1653-4. Epub 2016/03/16. doi: 10.1001/jama.2015.19058. PubMed PMID: 26978601; PMCID: PMC5297370.

2. Rigoni GC. Drug Utilization for Immediate- and Modified Release Opioids in the U.S. Division of Surveillance, Research & Communication Support, Office of Drug Safety, Food and Drug Administration, Silver Spring: MD. 2003 Accessed July 6, 2015. Available from: https://www.fda.gov/ohrms/dockets/ac/03/slides/3978S1_05_Rigoni.ppt.

3. Levy B, Paulozzi L, Mack KA and Jones CM. Trends in Opioid Analgesic-Prescribing Rates by Specialty, U.S., 2007-2012. Am J Prev Med. 2015;49(3):409-13. Epub 2015/04/22. doi: 10.1016/j.amepre.2015.02.020. PubMed PMID: 25896191; PMCID: PMC6034509.

4. Denisco RC, Kenna GA, O'Neil MG, Kulich RJ, Moore PA, Kane WT, Mehta NR, Hersh EV and Katz NP. Prevention of prescription opioid abuse: the role of the dentist. J Am Dent Assoc. 2011;142(7):800- 10. Epub 2011/07/02. PubMed PMID: 21719802.
5. Manchikanti L, Helm S, 2nd, Fellows B, Janata JW, Pampati V, Grider JS and Boswell MV. Opioid epidemic in the United States. Pain Physician. 2012;15(3 Suppl):ES9-38. Epub 2012/07/20. PubMed PMID: 22786464.
6. Somerman MJ and Volkow ND. The role of the oral health community in addressing the opioid overdose epidemic. J Am Dent Assoc. 2018;149(8):663-5. Epub 2018/07/30. doi: 10.1016/j.adaj.2018.06.010. PubMed PMID: 30055662.
7. McCauley JL, Hyer JM, Ramakrishnan VR, Leite R, Melvin CL, Fillingim RB, Frick C and Brady KT. Dental opioid prescribing and multiple opioid prescriptions among dental patients: Administrative data from the South Carolina prescription drug monitoring program. J Am Dent Assoc. 2016;147(7):537-44. Epub 2016/04/09. doi: 10.1016/j.adaj.2016.02.017. PubMed PMID: 27055600; PMCID: PMC4925262.
8. Gabay M. Prescription Drug Monitoring Programs. Hosp Pharm. 2015;50(4):277-8. Epub 2015/10/09. doi: 10.1310/hpj5004-277. PubMed PMID: 26445918; PMCID: PMC4589875.
9. Sirintawat N, Sawang K, Chaiyasamut T and Wongsirichat N. Pain measurement in oral and maxillofacial surgery. J Dent Anesth Pain Med. 2017;17(4):253-63. Epub 2018/01/20. doi: 10.17245/jdapm.2017.17.4.253. PubMed PMID: 29349347; PMCID: PMC5766084.

10. Meechan JG and Seymour RA. The use of third molar surgery in clinical pharmacology. Br, J Oral Maxillofac Surg. 1993;31(6):360-65. doi: https://doi.org/10.1016/0266-4356(93)90191-X.

11. Barden J, Edwards JE, McQuay HJ and Andrew Moore R. Pain and analgesic response after third molar extraction and other postsurgical pain. Pain. 2004;107(1):86-90. PubMed PMID: 14715393.

12. Cooper SA and Desjardins PJ. The value of the dental impaction pain model in drug development. Methods Mol Biol. 2010;617:175-90. Epub 2010/03/26. doi: 10.1007/978-1-60327-323-7_15. PubMed PMID: 20336423.

13. Singla NK, Desjardins PJ and Chang PD. A comparison of the clinical and experimental characteristics of four acute surgical pain models: dental extraction, bunionectomy, joint replacement, and soft tissue surgery. Pain. 2014;155(3):441-56. Epub 2013/09/10. doi: 10.1016/j.pain.2013.09.002. PubMed PMID: 24012952.

14. Moore RA, Derry S Fau - Aldington D, Aldington D Fau - Wiffen PJ and Wiffen PJ. Single dose oral analgesics for acute postoperative pain in adults - an overview of Cochrane reviews(1469-493X (Electronic)).

RUTGERS UNIVERSITY
Office for Research
APPROVED
IRB ID: Pro2020002299
APPROVAL: 1/8/2025
EXPIRATION: 1/7/2026

15. Moore RA, Wiffen PJ, Derry S, Maguire T, Roy YM and Tyrrell L. Non-prescription (OTC) oral analgesics for acute pain - an overview of Cochrane reviews. Cochrane Database Syst Rev. 2015(11):CD010794. Epub 2015/11/07. doi: 10.1002/14651858.CD010794.pub2. PubMed PMID: 26544675.
16. Bailey E, Worthington HV, van Wijk A, Yates JM, Coulthard P and Afzal Z. Ibuprofen and/or paracetamol (acetaminophen) for pain relief after surgical removal of lower wisdom teeth. Cochrane Database Syst Rev. 2013(12):CD004624. Epub 2013/12/18. doi: 10.1002/14651858.CD004624.pub2. PubMed PMID: 24338830.
17. Derry CJ, Derry S and Moore RA. Single dose oral ibuprofen plus paracetamol (acetaminophen) for acute postoperative pain. Cochrane Database Syst Rev. 2013(6):CD010210. Epub 2013/06/25. doi: 10.1002/14651858.CD010210.pub2. PubMed PMID: 23794268.
18. Bailey E, Worthington H and Coulthard P. Ibuprofen and/or paracetamol (acetaminophen) for pain relief after surgical removal of lower wisdom teeth, a Cochrane systematic review. Br Dent J. 2014;216(8):451-5. Epub 2014/04/26. doi: 10.1038/sj.bdj.2014.330. PubMed PMID: 24762895.
19. Hyllested M, Jones S, Pedersen JL and Kehlet H. Comparative effect of paracetamol, NSAIDs or their combination in postoperative pain management: a qualitative review. Br J Anaesth. 2002;88(2):199-214. Epub 2002/03/07. PubMed PMID: 11878654.
20. Chang AK, Bijur PE, Esses D, Barnaby DP and Baer J. Effect of a Single Dose of Oral Opioid and Nonopioid Analgesics on Acute Extremity Pain in the Emergency Department: A Randomized Clinical Trial. JAMA. 2017;318(17):1661-7. Epub 2017/11/09. doi: 10.1001/jama.2017.16190. PubMed PMID: 29114833; PMCID: PMC5818795.
21. Daniels SE, Goulder MA, Aspley S and Reader S. A randomised, five-parallel-group, placebo-controlled trial comparing the efficacy and tolerability of analgesic combinations including a novel single-tablet combination of ibuprofen/paracetamol for postoperative dental pain. Pain. 2011;152(3):632-42. Epub 2011/01/25. doi: 10.1016/j.pain.2010.12.012. PubMed PMID: 21257263.
22. Bijur P. Comparing Five Oral Analgesics for Treatment of Acute Pain in the ED. ClinicalTrials.gov Identifier: NCT03173456. Albert Einstein College of Medicine, Inc; 2017.
23. Barkin RL. Acetaminophen, aspirin, or Ibuprofen in combination analgesic products. Am J Ther. 2001;8(6):433-42. Epub 2001/11/13. PubMed PMID: 11704782.
24. Singla A and Sloan P. Pharmacokinetic evaluation of hydrocodone/acetaminophen for pain management. J Opioid Manag. 2013;9(1):71-80. Epub 2013/05/28. doi: 10.5055/jom.2013.0149. PubMed PMID: 23709306.
25. Shah A, Hayes CJ and Martin BC. Characteristics of Initial Prescription Episodes and Likelihood of Long-Term Opioid Use - United States, 2006-2015. MMWR Morb Mortal Wkly Rep. 2017;66(10):265-9. Epub 2017/03/17. doi: 10.15585/mmwr.mm6610a1. PubMed PMID: 28301454; PMCID: PMC5657867.
26. Deyo RA, Hallvik SE, Hildebran C, Marino M, Dexter E, Irvine JM, O'Kane N, Van Otterloo J, Wright DA, Leichtling G and Millet LM. Association Between Initial Opioid Prescribing Patterns and Subsequent Long-Term Use Among Opioid-Naive Patients: A Statewide Retrospective Cohort Study. J Gen Intern Med. 2017;32(1):21-7. Epub 2016/08/04. doi: 10.1007/s11606-016-3810-3. PubMed PMID: 27484682; PMCID: PMC5215151.
27. Brummett CM, Waljee JF, Goesling J, Moser S, Lin P, Englesbe MJ, Bohnert ASB, Kheterpal S and Nallamothu BK. New Persistent Opioid Use After Minor and Major Surgical Procedures in US Adults. JAMA Surg. 2017;152(6):e170504. Epub 2017/04/14. doi: 10.1001/jamasurg.2017.0504. PubMed PMID: 28403427.
28. Hoppe JA, Kim H and Heard K. Association of emergency department opioid initiation with recurrent

RUTGERS UNIVERSITY Office for Research
APPROVED
IRB ID: Pro2020002299
APPROVAL: 1/8/2025
EXPIRATION: 1/7/2026

opioid use. Ann Emerg Med. 2015;65(5):493-9 e4. Epub 2014/12/24. doi: 10.1016/j.annemergmed.2014.11.015. PubMed PMID: 25534654.

29. Alam A, Gomes T, Zheng H, Mamdani MM, Juurlink DN and Bell CM. Long-term analgesic use after low-risk surgery: a retrospective cohort study. Arch Intern Med. 2012;172(5):425-30. Epub 2012/03/14. doi: 10.1001/archinternmed.2011.1827. PubMed PMID: 22412106.

30. Barnett ML, Olenksi AR and Jena AB. Opioid Prescribing by Emergency Physicians and Risk of Long-Term Use. N Engl J Med. 2017;376(19):1896. Epub 2017/05/11. doi: 10.1056/NEJMc1703338. PubMed PMID: 28489999.

31. O'Neil M. The ADA practical guide to substance use disorders and safe prescribing. Hoboken, NJ: John Wiley & Sons, Inc; 2015.

32. Moore PA and Hersh EV. Combining ibuprofen and acetaminophen for acute pain management after third-molar extractions: translating clinical research to dental practice. J Am Dent Assoc. 2013;144(8):898-908. Epub 2013/08/02. PubMed PMID: 23904576.

33. Au AH, Choi SW, Cheung CW and Leung YY. The Efficacy and Clinical Safety of Various Analgesic Combinations for Post-Operative Pain after Third Molar Surgery: A Systematic Review and Meta-Analysis. PLoS One. 2015;10(6):e0127611. Epub 2015/06/09. doi: 10.1371/journal.pone.0127611. PubMed PMID: 26053953; PMCID: PMC4459961.

34. Moore PA, Ziegler KM, Lipman RD, Aminoshariae A, Carrasco-Labra A and Mariotti A. Benefits and harms associated with analgesic medications used in the management of acute dental pain: An overview of systematic reviews. J Am Dent Assoc. 2018;149(4):256-65 e3. Epub 2018/03/31. doi: 10.1016/j.adaj.2018.02.012. PubMed PMID: 29599019.

35. Moore RA, Derry S, Aldington D and Wiffen PJ. Single dose oral analgesics for acute postoperative pain in adults - an overview of Cochrane reviews. Cochrane Database Syst Rev. 2015(9):CD008659. Epub 2015/09/29. doi: 10.1002/14651858.CD008659.pub3. PubMed PMID: 26414123.

36. Gaskell H, Derry S, Moore RA and McQuay HJ. Single dose oral oxycodone and oxycodone plus paracetamol (acetaminophen) for acute postoperative pain in adults. Cochrane Database Syst Rev. 2009(3):CD002763. Epub 2009/07/10. doi: 10.1002/14651858.CD002763.pub2. PubMed PMID: 19588335; PMCID: PMC4170904.

37. Toms L, Derry S, Moore RA and McQuay HJ. Single dose oral paracetamol (acetaminophen) with codeine for postoperative pain in adults. Cochrane Database Syst Rev. 2009(1):CD001547. Epub 2009/01/23. doi: 10.1002/14651858.CD001547.pub2. PubMed PMID: 19160199; PMCID: PMC4171965.

38. Hadhimane A, Shankariah M and Neswi KV. Pre-Emptive Analgesia with Ketamine for Relief of Postoperative Pain After Surgical Removal of Impacted Mandibular Third Molars. J Maxillofac Oral Surg. 2016;15(2):156-63. Epub 2016/06/15. doi: 10.1007/s12663-015-0813-2. PubMed PMID: 27298538; PMCID: PMC4871823.

39. Janakiram C, Chalmers NI, Fontelo P, Huser V, Lopez Mitnik G, Iafolla TJ, Brow AR and Dye BA. Sex and race or ethnicity disparities in opioid prescriptions for dental diagnoses among patients receiving Medicaid. J Am Dent Assoc. 2018;149(4):246-55. Epub 2018/03/31. doi: 10.1016/j.adaj.2018.02.010. PubMed PMID: 29599018.

40. Bartley EJ and Fillingim RB. Sex differences in pain: a brief review of clinical and experimental findings. Br J Anaesth. 2013;111(1):52-8. Epub 2013/06/26. doi: 10.1093/bja/aet127. PubMed PMID: 23794645; PMCID: PMC3690315.

41. Gear RW, Miaskowski C, Gordon NC, Paul SM, Heller PH and Levine JD. Kappa-opioids produce significantly greater analgesia in women than in men. Nat Med. 1996;2(11):1248-50. Epub

RUTGERS UNIVERSITY
Office for Research
APPROVED
IRB ID: Pro2020002299
APPROVAL: 1/8/2025
EXPIRATION: 1/7/2026

1996/11/01. PubMed PMID: 8898754.

42. Greenspan JD, Craft RM, LeResche L, Arendt-Nielsen L, Berkley KJ, Fillingim RB, Gold MS, Holdcroft A, Lautenbacher S, Mayer EA, Mogil JS, Murphy AZ, Traub RJ, Consensus Working Group of the Sex G and Pain SIGotI. Studying sex and gender differences in pain and analgesia: a consensus report.

43. McCabe SE, West BT and Boyd CJ. Leftover prescription opioids and nonmedical use among high school seniors: a multi-cohort national study. J Adolesc Health. 2013;52(4):480-5. Epub 2013/01/10. doi: 10.1016/j.jadohealth.2012.08.007. PubMed PMID: 23298996; PMCID: PMC3608842.

44. Ashrafioun L, Edwards PC, Bohnert AS and Ilgen MA. Nonmedical use of pain medications in dental patients. Am J Drug Alcohol Abuse. 2014;40(4):312-6. Epub 2014/06/26. doi: 10.3109/00952990.2014.930152. PubMed PMID: 24963730.

45. Maughan BC, Hersh EV, Shofer FS, Wanner KJ, Archer E, Carrasco LR and Rhodes KV. Unused opioid analgesics and drug disposal following outpatient dental surgery: A randomized controlled trial.Drug Alcohol Depend. 2016;168:328-34. Epub 2016/10/30. doi: 10.1016/j.drugalcdep.2016.08.016. PubMed PMID: 27663358.

46. Menhinick KA, Gutmann JL, Regan JD, Taylor SE and Buschang PH. The efficacy of pain control following nonsurgical root canal treatment using ibuprofen or a combination of ibuprofen and acetaminophen in a randomized, double-blind, placebo-controlled study. Int Endod J. 2004;37(8):531-41. Epub 2004/07/03. doi: 10.1111/j.1365-2591.2004.00836.x. PubMed PMID: 15230906.

47. Bartley EJ and Fillingim RB. Sex differences in pain: a brief review of clinical and experimental findings. Br J Anaesth. 2013;111(1):52-8. Epub 2013/06/26. doi: 10.1093/bja/aet127. PubMed PMID: 23794645; PMCID: PMC3690315.

48. Ong CK, Seymour RA, Lirk P and Merry AF. Combining paracetamol (acetaminophen) with nonsteroidal antiinflammatory drugs: a qualitative systematic review of analgesic efficacy for acute postoperative pain. Anesth Analg. 2010;110(4):1170-9. Epub 2010/02/10. doi: 10.1213/ANE.0b013e3181cf9281. PubMed PMID: 20142348.

49. Kochanek KD, Murphy S, Xu J and Arias E. Mortality in the United States, 2016. NCHS Data Brief. 2017(293):1-8. Epub 2018/01/11. PubMed PMID: 29319473.

50. Wide-ranging online data for epidemiologic research (WONDER). Atlanta, GA: CDC, National Center for Health Statistics. 2017.

51. McCauley JL, Leite RS, Melvin CL, Fillingim RB and Brady KT. Dental opioid prescribing practices and risk mitigation strategy implementation: Identification of potential targets for provider-level intervention. Subst Abus. 2016;37(1):9-14. Epub 2015/12/18. doi: 10.1080/08897077.2015.1127870. PubMed PMID: 26675303; PMCID: PMC4816206.

52. Bicket MC, Long JJ, Pronovost PJ, Alexander GC and Wu CL. Prescription Opioid Analgesics Commonly Unused After Surgery: A Systematic Review. JAMA Surg. 2017;152(11):1066-71. Epub 2017/08/03. doi: 10.1001/jamasurg.2017.0831. PubMed PMID: 28768328; PMCID: PMC5701659.

53. Miech R, Johnston L, O'Malley PM, Keyes KM and Heard K. Prescription Opioids in Adolescence and Future Opioid Misuse. Pediatrics. 2015;136(5):e1169-77. Epub 2015/10/28. doi: 10.1542/peds.2015-1364. PubMed PMID: 26504126; PMCID: PMC4834210.

54. Rudd RA, Seth P, David F and Scholl L. Increases in Drug and Opioid-Involved Overdose Deaths - United States, 2010-2015. MMWR Morb Mortal Wkly Rep. 2016;65(5051):1445-52. Epub 2016/12/30. doi: 10.15585/mmwr.mm655051e1. PubMed PMID: 28033313.

55. Fricke JR, Jr., Karim R, Jordan D and Rosenthal N. A double-blind, single-dose comparison of the analgesic efficacy of tramadol/acetaminophen combination tablets, hydrocodone/acetaminophen combination tablets, and placebo after oral surgery. Clin Ther. 2002;24(6):953-68. Epub 2002/07/16.

RUTGERS UNIVERSITY Office for Research
APPROVED
IRB ID: Pro2020002299
APPROVAL: 1/8/2025
EXPIRATION: 1/7/2026

PubMed PMID: 12117085.

56. Niebler G and Dayno J. Effect size comparison of ketorolac nasal spray and commonly prescribed oral combination opioids for pain relief after third molar extraction surgery. Postgrad Med. 2016;128(1):12-7. Epub 2015/12/01. doi: 10.1080/00325481.2016.1126185. PubMed PMID: 26616001.

57. Forbes JA, Bowser MW, Calderazzo JP and Foor VM. An evaluation of the analgesic efficacy of three opioid-analgesic combinations in postoperative oral surgery pain. J Oral Surg. 1981;39(2):108-12. Epub 1981/02/01. PubMed PMID: 6936524.

58. Hewitt DJ, Todd KH, Xiang J, Jordan DM, Rosenthal NR and Investigators C-S. Tramadol/acetaminophen or hydrocodone/acetaminophen for the treatment of ankle sprain: a randomized, placebo-controlled trial. Ann Emerg Med. 2007;49(4):468-80, 80 e1-2. Epub 2006/11/23. doi: 10.1016/j.annemergmed.2006.08.030. PubMed PMID: 17113683.

59. Turturro MA, Paris PM and Larkin GL. Tramadol versus hydrocodone-acetaminophen in acute musculoskeletal pain: a randomized, double-blind clinical trial. Ann Emerg Med. 1998;32(2):139-43. Epub 1998/08/13. PubMed PMID: 9701294.

60. Fricke J, Halladay SC, Bynum L and Francisco CA. Pain relief after dental impaction surgery using ketorolac, hydrocodone plus acetaminophen, or placebo. Clin Ther. 1993;15(3):500-9. Epub 1993/05/01. PubMed PMID: 8364942.

61. Forbes JA, Bates JA, Edquist IA, Burchfield WH, Smith FG, Schwartz MK, Kit V, Hyatt J, Bell WE and Beaver WT. Evaluation of two opioid-acetaminophen combinations and placebo in postoperative oral surgery pain. Pharmacotherapy. 1994;14(2):139-46. Epub 1994/03/01. PubMed PMID: 8197031.

62. Litkowski LJ, Christensen SE, Adamson DN, Van Dyke T, Han SH and Newman KB. Analgesic efficacy and tolerability of oxycodone 5 mg/ibuprofen 400 mg compared with those of oxycodone 5 mg/acetaminophen 325 mg and hydrocodone 7.5 mg/acetaminophen 500 mg in patients with moderate to severe postoperative pain: a randomized, double-blind, placebo-controlled, single-dose, parallel- group study in a dental pain model. Clin Ther. 2005;27(4):418-29. Epub 2005/06/01. doi: 10.1016/j.clinthera.2005.04.010. PubMed PMID: 15922815.

63. Kea B, Fu R, Lowe RA and Sun BC. Interpreting the National Hospital Ambulatory Medical Care Survey: United States Emergency Department Opioid Prescribing, 2006-2010. Acad Emerg Med. 2016;23(2):159-65. Epub 2016/01/24. doi: 10.1111/acem.12862. PubMed PMID: 26802501; PMCID: PMC4946851.

64. Mutlu I, Abubaker AO and Laskin DM. Narcotic prescribing habits and other methods of pain control by oral and maxillofacial surgeons after impacted third molar removal. J Oral Maxillofac Surg. 2013;71(9):1500-3. Epub 2013/08/21. doi: 10.1016/j.joms.2013.04.031. PubMed PMID: 23948362.

65. Weiland BM, Wach AG, Kanar BP, Castele MT, Sosovicka MF, Cooke MR and Moore PA. Use of opioid pain relievers following extraction of third molars. Compend Contin Educ Dent. 2015;36(2):107-11; quiz 12, 14. Epub 2015/03/31. PubMed PMID: 25822637.

66. Moore SA, Nahourali HS and Zovko JG. Dental therapeutic practice patterns in the U.S. II. Analgesics, corticosteroids, and antibiotics. Gen Dent. 2016;54(3):201-7. PubMed PMID: 16776415.

67. Steinmetz CN, Zheng C, Okunseri E, Szabo A and Okunseri C. Opioid Analgesic Prescribing Practices of Dental Professionals in the United States. JDR Clin Trans Res. 2017;2(3):241-8. Epub 2017/09/08. doi: 10.1177/2380084417693826. PubMed PMID: 28879246; PMCID: PMC5576054.

68. Okunseri C, Okunseri E, Xiang Q, Thorpe JM and Szabo A. Prescription of opioid and nonopioid analgesics for dental care in emergency departments: Findings from the National Hospital Ambulatory

RUTGERS UNIVERSITY Office for Research
APPROVED
IRB ID: Pro2020002299
APPROVAL: 1/8/2025
EXPIRATION: 1/7/2026

Medical Care Survey. J Public Health Dent. 2014;74(4):283-92. Epub 2014/05/28. doi: 10.1111/jphd.12055. PubMed PMID: 24863407; PMCID: PMC4245386.

69. Mehlisch DR, Aspley S, Daniels SE and Bandy DP. Comparison of the analgesic efficacy of concurrent ibuprofen and paracetamol with ibuprofen or paracetamol alone in the management of moderate to severe acute postoperative dental pain in adolescents and adults: a randomized, double-blind, placebo- controlled, parallel-group, single-dose, two-center, modified factorial study. Clin Ther. 2010;32(5):882- 95. Epub 2010/08/06. doi: 10.1016/j.clinthera.2010.04.022. PubMed PMID: 20685496.

70. Mehlisch DR, Aspley S, Daniels SE, Southerden KA and Christensen KS. A single-tablet fixed-dose combination of racemic ibuprofen/paracetamol in the management of moderate to severe postoperative dental pain in adult and adolescent patients: a multicenter, two-stage, randomized, double-blind, parallel-group, placebo-controlled, factorial study. Clin Ther. 2010;32(6):1033-49. Epub 2010/07/20. doi: 10.1016/j.clinthera.2010.06.002. PubMed PMID: 20637958.

71. Mitchell A, McCrea P, Inglis K and Porter G. A randomized, controlled trial comparing acetaminophen plus ibuprofen versus acetaminophen plus codeine plus caffeine (Tylenol 3) after outpatient breast surgery. Ann Surg Oncol. 2012;19(12):3792-800. Epub 2012/06/21. doi: 10.1245/s10434-012-2447-7. PubMed PMID: 22713999.

72. Mitchell A, van Zanten SV, Inglis K and Porter G. A randomized controlled trial comparing acetaminophen plus ibuprofen versus acetaminophen plus codeine plus caffeine after outpatient general surgery. J Am Coll Surg. 2008;206(3):472-9. Epub 2008/03/01. doi: 10.1016/j.jamcollsurg.2007.09.006. PubMed PMID: 18308218.

73. Sniezek PJ, Brodland DG and Zitelli JA. A randomized controlled trial comparing acetaminophen, acetaminophen and ibuprofen, and acetaminophen and codeine for postoperative pain relief after Mohs surgery and cutaneous reconstruction. Dermatol Surg. 2011;37(7):1007-13. Epub 2011/05/13. doi: 10.1111/j.1524-4725.2011.02022.x. PubMed PMID: 21561527.

74. Best AD, De Silva RK, Thomson WM, Tong DC, Cameron CM and De Silva HL. Efficacy of Codeine When Added to Paracetamol (Acetaminophen) and Ibuprofen for Relief of Postoperative Pain After Surgical Removal of Impacted Third Molars: A Double-Blinded Randomized Control Trial. J Oral Maxillofac Surg. 2017;75(10):2063-9. Epub 2017/06/07. doi: 10.1016/j.joms.2017.04.045. PubMed PMID: 28586638.

75. Graudins A, Meek R, Parkinson J, Egerton-Warburton D and Meyer A. A randomised controlled trial of paracetamol and ibuprofen with or without codeine or oxycodone as initial analgesia for adults with moderate pain from limb injury. Emerg Med Australas. 2016;28(6):666-72. Epub 2016/09/08. doi: 10.1111/1742-6723.12672. PubMed PMID: 27599896.

76. Julious SA. Sample sizes for clinical trials with normal data. Stat Med. 2004;23(12):1921-86. Epub: 2004/06/15. doi: 10.1002/sim.1783. PubMed PMID: 15195324.

77. Breslow NE and Clayton DG. Approximate Inference in Generalized Linear Mixed Models. Journal ofthe American Statistical Association. 1993;88(421):9-25. doi: 10.2307/2290687.

78. D'Agostino RB, Sr., Massaro JM and Sullivan LM. Non-inferiority trials: design concepts and issues - the encounters of academic consultants in statistics. Stat Med. 2003;22(2):169-86. doi:10.1002/sim.1425. PubMed PMID: 12520555.

79. Hawker GA, Mian S, Kendzerska T and French M. Measures of adult pain: Visual Analog Scale for Pain (VAS Pain), Numeric Rating Scale for Pain (NRS Pain), McGill Pain Questionnaire (MPQ),Short-Form McGill Pain Questionnaire (SF-MPQ), Chronic Pain Grade Scale (CPGS), Short Form-36 Bodily Pain Scale (SF-36 BPS), and Measure of Intermittent and Constant Osteoarthritis Pain (ICOAP). Arthritis Care Res (Hoboken). 2011;63(Supp. 11):S240-52. doi: 10.1002/acr.20543. PubMed PMID:

RUTGERS UNIVERSITY
Office for Research
APPROVED
IRB ID: Pro2020002299
APPROVAL: 1/8/2025
EXPIRATION: 1/7/2026

22588748

80. Todd KH, Funk KG, Funk JP and Bonacci R. Clinical significance of reported changes in pain severity.Ann Emerg Med. 1996;27(4):485-9. Epub 1996/04/01. PubMed PMID: 8604867.

81. Gallagher EJ, Liebman M and Bijur PE. Prospective validation of clinically important changes in pain severity measured on a visual analog scale. Ann Emerg Med. 2001;38(6):633-8. Epub 2001/11/24. doi:10.1067/mem.2001.118863. PubMed PMID: 11719741.

82. Huitfeldt B, Hummel J and European Federation of Statisticians in the Pharmaceutical I. The draft FDA guideline on non-inferiority clinical trials: a critical review from European pharmaceutical industry statisticians. Pharm Stat. 2011;10(5):414-9. Epub 2011/09/21. doi: 10.1002/pst.508. PubMed PMID: 21932294.

83. Lewis JA. Switching between superiority and non-inferiority: an introductory note. Br J Clin Pharmacol.2001;52(3):221. Epub 2001/09/19. PubMed PMID: 11560552; PMCID: PMC2014545.

84. Xu Y, Yu M, Zhao YQ, Li Q, Wang S and Shao J. Ragulaized outcome weighted subgroup identification for differential treatment. Biometrics. 2015;71(3):645-53.

85. Qian M and Murphy SA. Performance Guarantees for Individualized Treatment Rules. Ann Stat.2011;39(2):1180-210. Epub 2011/06/15. doi: 10.1214/10-AOS864. PubMed PMID: 21666835; PMCID: PMC3110016.

86. Zhao Y, Zeng D, Rush AJ and Kosorok MR. Estimating Individualized Treatment Rules Using OutcomeWeighted Learning. J Am Stat Assoc. 2012;107(449):1106-18. Epub 2013/05/01. doi:10.1080/01621459.2012.695674. PubMed PMID: 23630406; PMCID: PMC3636816.

87. Tibshirani R, Saunders M, Rosset S, Zhu J and Knight K. Sparsity and Smoothness via the Fused Lasso. Journal of the Royal Statistical Society Series B (Statistical Methodology). 2005;67(1):91-108.

88. Chakraborty, B., Laber, E. B., and Zhao, Y. (2013). Inference for optimal dynamic treatment regimes using an adaptive mout-of-n bootstrap scheme. Biometrics 69, 714–23.

89. Bickel, P. J., G¨otze, F., and van Zwet, W. R. (1997). Resampling fewer than n observations: Gains, losses, and remedies for losses. Statistica Sinica 7, 1–31.

90. Raghunathan, T. E, Berglund, P., and Solenberger, P. W. (2017). Multiple Imputation in Practice: With Examples Using IVEware. Boca Raton: CRC Press.

RUTGERS UNIVERSITY
Office for Research

APPROVED
IRB ID: Pro2020002299
APPROVAL: 1/8/2025
EXPIRATION: 1/7/2026

APPENDICES

Table of Contents

Appendix	Page
Appendix A: Schedule of Events	78
Appendix B: Study Medication Package Inserts	79

RUTGERS UNIVERSITY
Office for Research
APPROVED
IRB ID: Pro2020002299
APPROVAL: 1/8/2025
EXPIRATION: 1/7/2026

APPENDIX A: SCHEDULE OF EVENTS

			Post-Operative Period							
Procedures	Screening (Visit 0)	Study Visit 1: Surgery (Day 1)	Upon Waking in the Morning (Days 2 to 10 +/-5)	When taking Pain Meds (Days 1to 10 +/-5)	Before Going to Sleep in the Evening (Days 1 to 9 +/-5)	Intermediate Visit Days 1 to 10 (+/- 5)	Post Operative Visit Study Visit 2 (Day 10 +/-5)	Withdrawal or Termination	PDMP Query (Visit 2 plus 186 days +/- 14 days)	Provide Reference to Counseling Session
Signed Consent Form	X									
Assessment of Eligibility Criteria (including review of medical history and concomitant medications)	X									
Study Intervention		X	X	X	X					
Pain Assessment	X	X								
Pain Interference Assessment					X		X			
Sleeping Quality			X				X			
Assessment of Adverse Events			X		X		X	X		
Obtain Satisfaction							X			
Determination of Tablets for Diversion							X			
Premature Exit study Documentation								X		
PDMP Inquiry									X	
PDMP Follow-up										X

RUTGERS UNIVERSITY
Office for Research

APPROVED
IRB ID: Pro2020002299
APPROVAL: 1/8/2025
EXPIRATION: 1/7/2026

Appendix B: Study Medication Label Inserts

Study Medication

DRUG	NDC #	DRUG LABEL- US National Library of Medicine
Hydrocodone 5mg/ acetaminophen 300 mg	00406-0376	https://dailymed.nlm.nih.gov/dailymed/drugInfo.cfm?setid=d621b 526-4d9a-48a9-9a3e-d29d6aea2f31
Ibuprofen 400 mg	67877-319	https://dailymed.nlm.nih.gov/dailymed/lookup.cfm?setid=9ce036 ef-a22d-4fca-9a62-1b10978f7b26
Acetaminophen 500 mg	50580-449<br><br>50580-937-07	https://dailymed.nlm.nih.gov/dailymed/lookup.cfm?setid=597738 93-09a8-47a2-943a-e9ea9da4458a<br>OR<br>https://dailymed.nlm.nih.gov/dailymed/drugInfo.cfm?setid=103d109d-f520-409c-8da2-eb6b0fbec891

RUTGERS UNIVERSITY
Office for Research

APPROVED
IRB ID: Pro2020002299
APPROVAL: 1/8/2025
EXPIRATION: 1/7/2026
