# Supplementary material for: Analgesic Differences in Males and Females After Third Molar Surgery: A Subgroup Analysis of the OARS Randomized Clinical Trial
Source: JAMA Netw Open. 2025 Nov 6;8(11):e2542467. doi: 10.1001/jamanetworkopen.2025.42467 (PMC12593115; doi:10.1001/jamanetworkopen.2025.42467)
Supplement: Supplement 3. — Data Sharing Statement [file jamanetwopen-e2542467-s003.pdf]

Data Sharing Statement

Fredericks-Younger. Analgesic Differences in Males and Females After Third Molar Surgery: OARS Subgroup Analysis. *JAMA Netw Open.* Published online November 6, 2025. doi:10.1001/jamanetworkopen.2025.42467

Data

Additional Information: Clinicaltrials.gov:NCT04452344
Data available: Yes
Data types: Deidentified participant data, Data dictionary
How to access data: Dr. Cecile A. Feldman, DMD, at feldman@rutgers.edu
When available: beginning date: 01-01-2025, end date: 12-30-2030

Supporting Documents

Document types: Informed consent form
How to access documents: Dr. Cecile A. Feldman, DMD, at feldman@rutgers.edu
When available: beginning date: 01-01-2025, end date: 12-30-2030

Additional Information

Who can access the data: Researchers who provide a research analysis plan that aims to enhance patient care and clinical outcomes.
Types of analyses: Researchers who provide a research analysis plan that aims to enhance patient care and clinical outcomes.
Mechanisms of data availability: Available after completion of a data access agreement with Rutgers University.
Any additional restrictions: N/A
